# Supplementary material for: Seroprevalence Assessment and Risk Factor Analysis of Toxoplasma gondii Infection in Goats from Northeastern Algeria
Source: Animals (Basel). 2024 Mar 13;14(6):883. doi: 10.3390/ani14060883 (PMC10967517; doi:10.3390/ani14060883)
Supplement: Supplementary file 1 [file animals-14-00883-s001.zip › Supplementary Material Table S1.pdf]

Table S1. Univariate analysis of explanatory risk factors associated to *T. gondii* infection goat population from Northeastern Algeria, with P-value ≥ 0.05.

Variable	Categories	Negative	Positive (%)	OR (95% CI)	P-value
**Individual related factors**					
Gender	Male	73	85 (53.8)	1.0334 (0.7026-1.52)	0.862
	Female	142	160 (52.98)		
Age	<2 years	89	92 (50.83)	—	0.272
	2-5 years	88	118 (57.28)	0.7709 (0.516- 1.1517)	
	>5 years	38	35 (47.95)	0.6869 (0.402- 1.1737)	
Body conditions	Poor	59	55 (48.25)	—	0.597
	Medium	47	56 (54.37)	0.7824 (0.4586-1.3347)	
	Good	100	120 (54.55)	1.0071 (0.6296-1.6112)	
	Very good	9	14 (60.87)	0.7714 (0.3205-1.857)	
	Non breeder	49	42 (46.15)		
Management system	Extensive	13	11 (45.83)	—	0.102
	Intensive	7	20 (74.07)	0.2962 (0.0913-0.961)	
	Semi-intensive	120	141 (54.02)	0.4113 (0.1681-1.006)	
	Semi-extensive	75	73 (49.32)	1.2072 (0.8061-1.8079)	
**Herd related factors**					
Type of production	Milk	80	97 (54.8)	—	0.804
	Meat	31	37 (54.41)	1.0159 (0.5794-1.7813)	
	Mixed	104	111 (51.63)	0.8942 (0.5174-1.5454)	
Herd size	1-10	106	103 (49.28)	—	0.294
	11-30	101	131 (56.47)	0.7492 (0.5147-1.0904)	
	≥31	8	11 (57.89)	1.0601 (0.4112-2.7329)	
Size of pasture area	10H	80	101 (55.80)	—	0.059
	11-50H	124	122 (49.59)	1.2832 (0.8728-1.8865)	
	≥51H	4	2 (33.33)	0.5082 (0.0914-2.8259)	
Type of pasture area	Plain	77	85 (52.47)	—	0.160
	Hill	121	131 (51.98)	1.0196 (0.6868-1.5138)	
	Mountain	10	9 (47.37)	0.8313 (0.3267-2.1151)	
Common pastures	No	134	140 (51.09)	0.9096 (0.615- 1.3453)	0.631
	Yes	74	85 (53.46)		
Use of concentrate feed	No	76	68	1.423 (0.958-2.114)	0.080
	Yes	139	177		
Watering type	Surface water	129	165 (56.12)	1.375 (0.9386- 2.0142	0.101
	Surface and deep water	86	80 (48.19)		
Water source	Tap water	111	142 (56.13)	—	0.218
	Tap + valley water	30	37 (55.22)	1.0373 (0.6034-1.7831)	
	Tap + well water	74	66 (47.14)	0.7232 (0.403-1.2978)	
Location of the water trough	Internal	107	134 (55.6)	—	0.444
	External	14	11 (44.00)	1.5939 (0.6953-3.6538)	
	Mixed	94	100 (51.55)	1.354 (0.5855-3.1313)	
Species on the farm	Goats	78	104 (57.14)	1.2955 (0.8894-1.8871)	0.177
	Goats+Sheep	137	141 (50.72)		
Presence of other farm animals	No	3	3 (50)	1.1415 (0.228-5.716)	0.872
	Yes	212	242 (53.3)		
Presence of cattle	No	131	147 (52.88)	1.0397 (0.7148-1.5123)	0.841
	Yes	84	98 (53.85)		
Presence of equids	No	199	223 (52.84)	1.227 (0.6268-2.4021)	0.548
	Yes	16	22 (57.89)		
Presence of	No	11	138 (55.42)	0.8276 (0.5729-1.1955)	0.312

poultry	Yes	104	107 (50.71)		
Presence of rodents	No<br>Yes	157<br>58	173 (52.42)<br>72 (55.38)	1.1266 (0.7493-1.6937)	0.565
**Cat-related factors**					
Presence of cats	No<br>Yes	47<br>168	51 (52.04)<br>194 (53.59)	1.0642 (0.6807-1.6638)	0.791
Access of cats to feed	No<br>Yes	155<br>60	160 (50.79)<br>85 (58.62)	1.3724 (0.9222-2.0423)	0.118
Access of cats to water	No<br>Yes	86<br>129	103 (54.5)<br>142 (52.4)	0.9191 (0.6332-1.334)	0.654
Cats for control of rodents	No<br>Yes	168<br>47	174 (50.88)<br>71 (60.17)	0.6856 (0.4481-1.0489)	0.081
Control of cat's population	No<br>Yes	161<br>07	183 (53.2)<br>11 (61.11)	1.3825 (0.5236-3.6507)	0.511
Birth of cats in the farm	No<br>Yes	153<br>62	185 (54.73)<br>60 (49.18)	0.8003 (0.5287-1.2115)	0.292
Cats consume placenta	No<br>Yes	73<br>95	68 (48.23)<br>126 (57.01)	1.4238 (0.9315-2.1764)	0.102
Presence of wild cats	No<br>Yes	189<br>26	217 (53.45)<br>28 (51.85)	0.938 (0.5313-1.6558)	0.823
**Hygiene related factors**					
Quarantine	No<br>Yes	166<br>49	197 (54.27)<br>48 (49.48)	0.8254 (0.5272-1.2925)	0.402
Special place for parturition	No<br>Yes	180<br>35	199 (52.51)<br>46 (56.79)	1.1888 (0.7329-1.9282)	0.483
Exchange of breeder	No<br>Yes	124<br>91	151 (54.91)<br>94 (50.81)	0.8483 (0.5839-1.2324)	0.388
Slaughtering in the farm	No<br>Yes	201<br>14	229 (53.26)<br>16 (53.333)	1.003 (0.478-2.106)	0.993
**Disease and herd health related factors**					
Deworming	No<br>Yes	86<br>129	113 (56.78)<br>132 (50.57)	0.7788 (0.5374-1.1284)	0.185
Use of Antimicrobials	No<br>Yes	133<br>82	155 (53.82)<br>90 (52.33)	0.9418 (0.6451-1.375)	0.751
**Reproduction related factors**					
Reproductive disorders	No<br>Yes	15<br>200	11 (42.31)<br>234 (53.92)	1.5955 (0.7165-3.5529)	0.248
History of abortion	No<br>Yes	92<br>48	88 (48.89)<br>72 (60.00)	1.5682 (0.9823-2.5035)	0.058
Stillbirth	No<br>Yes	56<br>153	65 (53.72)<br>172 (52.92)	0.9685 (0.6373-1.4719)	0.887
New born death	No<br>Yes	138<br>71	141 (50.54)<br>96 (57.49)	1.3233 (0.8993-1.9473)	0.155
Elimination of aborted products	No<br>Yes	146<br>48	167 (53.35)<br>58 (54.72)	1.0564 (0.6788-1.644)	0.806
**Spatio-temporal related factors**					
Province	Mila<br>Constantine<br>Guelma<br>El-Taref	130<br>64<br>18<br>3	140 (51.85)<br>75 (53.96)<br>23 (56.10)<br>7 (70.00)	—<br>0.919 (0.6098-1.385)<br>0.9171 (0.4549-1.8491)<br>0.5476 (0.1238-2.4214)	0.682
Environment	Rural<br>Peri-urban	177<br>38	202 (53.3)<br>43 (53.09)	1.0085 (0.6236- 1.6312)	1
Climate	Humid	108	132 (55.00)	—	0.554

Sub-humid	102	105 (50.72)	1.1873 (0.8179-1.7236)
Semi-arid	5	8 (61.54)	0.6434 (0.2037-2.0322)

Ref.—reference value; OR—odds ratio; CI—confidence interval.
