# Supplementary material for: Seroprevalence Assessment and Risk Factor Analysis of Toxoplasma gondii Infection in Goats from Northeastern Algeria
Source: Animals (Basel). 2024 Mar 13;14(6):883. doi: 10.3390/ani14060883 (PMC10967517; doi:10.3390/ani14060883)
Supplement: Supplementary file 1 [file animals-14-00883-s001.zip › Supplementary Material Table S2.pdf]

Table S2. Sampling information of the 460 goat serum samples collected from Algeria.

N° Sample	N° Herd	Gender	Age	Body Conditions	Physiology status
1	1	M	A	Good	Non breeder
2	1	F	C	Good	Lacting
3	1	M	A	Good	Non breeder
4	1	F	B	Medium	Pregnant
5	1	F	B	Good	Lacting
6	1	M	B	Bad	Non breeder
7	1	F	A	Good	Pregnant
8	1	M	A	Good	Non breeder
9	1	M	C	V.good	Breeder
10	1	F	A	Good	Lacting
11	1	M	B	Good	Non breeder
12	1	F	A	Good	Pregnant
13	2	F	B	Good	Pregnant
14	2	M	B	Good	Breeder
15	2	F	C	Bad	Lacting
16	2	F	B	Good	Lacting
17	2	F	A	Good	Pregnant
18	3	M	A	Good	Non breeder
19	3	M	A	Bad	Breeder
20	3	F	B	Good	Lacting
21	3	F	B	Good	Pregnant
22	3	F	A	Good	Pregnant
23	3	F	A	Bad	Pregnant
24	3	F	B	V.good	Lacting
25	3	F	B	V.good	Lacting
26	4	F	A	Bad	Lacting
27	4	F	A	Good	Lacting
28	4	M	A	Good	Breeder
29	4	F	B	Good	Pregnant
30	4	F	B	Good	Lacting
31	4	F	A	Bad	Pregnant
32	4	F	A	Bad	Lacting
33	5	M	A	Good	Breeder
34	5	F	A	Good	Pregnant
35	5	F	A	Good	Lacting
36	5	F	A	Good	Lacting
37	6	M	A	Bad	Non breeder
38	6	F	B	Good	Lacting
39	6	F	A	Good	Pregnant
40	6	F	A	Bad	Lacting
41	6	M	B	Good	Breeder
42	6	F	A	Good	Lacting
43	6	F	B	Good	Pregnant
44	6	F	B	Bad	Pregnant
45	6	F	C	Good	Lacting
46	7	M	B	Good	Breeder
47	7	F	A	Good	Lacting

48	7	F	A	Bad	Pregnant
49	7	F	B	Good	Lacting
50	7	M	B	Bad	Non breeder
51	7	M	A	Good	Breeder
52	7	M	A	Bad	Non breeder
53	7	F	C	Bad	Lacting
54	7	F	B	Good	Lacting
55	7	F	A	Good	Pregnant
56	8	F	C	Good	Pregnant
57	8	M	B	Bad	Non breeder
58	8	F	B	Good	Lacting
59	8	F	B	Bad	Lacting
60	8	M	C	Bad	Breeder
61	8	M	C	Good	Breeder
62	8	F	B	Good	Pregnant
63	8	M	A	Bad	Non breeder
64	8	F	A	Bad	Pregnant
65	8	F	B	Good	Lacting
66	8	F	B	Good	Lacting
67	9	M	B	Good	Non breeder
68	9	F	A	Good	Lacting
69	9	F	A	Bad	Lacting
70	9	M	B	Bad	Non breeder
71	9	M	C	Good	Breeder
72	10	F	A	Good	Lacting
73	10	F	C	Good	Pregnant
74	10	M	C	Good	Breeder
75	10	F	B	Bad	Lacting
76	10	M	A	Bad	Non breeder
77	10	F	A	Good	Lacting
78	10	F	B	Good	Pregnant
79	11	F	B	Good	Lacting
80	11	F	A	Good	Pregnant
81	11	F	B	Bad	Pregnant
82	11	F	A	Bad	Lacting
83	11	F	B	Good	Lacting
84	11	F	C	Good	Pregnant
85	11	M	A	Good	Breeder
86	11	M	B	Bad	Non breeder
87	12	M	A	Good	Breeder
88	12	M	B	Bad	Non breeder
89	12	F	B	Bad	Lacting
90	12	F	B	Good	Pregnant
91	12	M	A	Good	Non breeder
92	12	M	B	Bad	Non breeder
93	12	F	B	Good	Pregnant
94	12	F	C	Good	Lacting
95	13	F	C	Good	Lacting
96	13	F	C	Good	Lacting
97	13	M	B	Good	Breeder

98	14	M	A	Good	Breeder
99	14	F	B	Good	Lacting
100	14	F	A	Bad	Lacting
101	14	F	C	Good	Pregnant
102	14	M	A	Bad	Non breeder
103	14	M	C	Bad	Non breeder
104	14	F	C	Good	Pregnant
105	14	F	C	Bad	Lacting
106	15	F	A	Good	Lacting
107	15	F	B	Bad	Pregnant
108	15	M	B	Good	Breeder
109	15	M	A	Good	Non breeder
110	15	F	C	Good	Lacting
111	16	F	C	Good	Pregnant
112	16	F	B	Bad	Lacting
113	16	M	C	Bad	Non breeder
114	16	M	B	Good	Breeder
115	16	F	A	Bad	Lacting
116	17	M	B	Good	Breeder
117	17	F	A	Bad	Lacting
118	17	M	B	Good	Non breeder
119	17	F	C	Good	Pregnant
120	17	F	B	Bad	Lacting
121	18	F	B	Good	Lacting
122	18	F	C	Good	Lacting
123	18	M	B	Good	Breeder
124	18	F	B	Bad	Pregnant
125	18	F	A	Good	Lacting
126	18	F	A	Bad	Pregnant
127	19	F	B	Good	Pregnant
128	19	F	C	Bad	Lacting
129	19	M	B	Good	Breeder
130	19	M	B	Good	Non breeder
131	19	M	A	Bad	Non breeder
132	20	M	C	Good	Breeder
133	20	F	B	Good	Lacting
134	20	M	C	Bad	Non breeder
135	20	F	B	Good	Lacting
136	20	F	B	Good	Lacting
137	20	F	A	Bad	Pregnant
140	21	F	B	Good	Pregnant
141	21	F	A	Good	Lacting
142	21	F	B	Bad	Lacting
143	21	F	B	Good	Pregnant
144	21	M	A	Bad	Non breeder
145	22	M	A	Bad	Non breeder
146	22	M	A	Good	Breeder
147	22	F	A	Good	Lacting
148	22	F	B	Good	Lacting
149	22	M	B	Good	Breeder

150	22	F	B	Bad	Lacting
151	22	F	A	Bad	Pregnant
153	23	F	B	Good	Pregnant
154	23	F	A	Bad	Lacting
155	23	M	A	Bad	Non breeder
156	23	M	A	Good	Non breeder
157	24	F	A	Good	Lacting
158	24	F	B	Good	Lacting
159	24	F	A	Good	Pregnant
160	24	M	B	Good	Breeder
161	25	F	C	Good	Lacting
162	25	F	C	Good	Lacting
163	25	F	A	Good	Pregnant
164	25	M	A	Good	Breeder
165	26	M	A	Good	Non breeder
166	26	M	B	Good	Breeder
167	26	F	C	Good	Lacting
168	26	F	B	Bad	Lacting
169	26	F	B	Bad	Pregnant
170	26	M	C	Bad	Non breeder
171	26	M	B	Good	Non breeder
172	26	F	B	Good	Pregnant
173	26	F	A	Bad	Lacting
174	27	F	C	Good	Lacting
175	27	M	C	Good	Breeder
176	27	F	C	Good	Lacting
177	27	F	C	Bad	Pregnant
178	27	M	A	Bad	Non breeder
179	27	F	C	Good	Pregnant
180	28	F	C	Good	Lacting
181	28	F	B	Bad	Lacting
182	28	M	C	Good	Breeder
183	28	M	A	Bad	Non breeder
184	28	F	B	Bad	Pregnant
185	29	F	A	Good	Lacting
186	29	F	C	Good	Pregnant
187	29	F	B	Bad	Lacting
188	29	M	C	Good	Breeder
189	29	F	A	Good	Non pregnant
190	29	M	C	Good	Non breeder
191	30	F	A	Good	Lacting
192	30	F	B	Good	Lacting
193	30	F	B	Medium	Pregnant
194	30	F	B	Bad	Lacting
195	30	M	A	Good	Breeder
196	31	M	C	Good	Breeder
197	31	F	B	V.good	Pregnant
198	31	F	B	Good	Lacting
199	31	M	A	Good	Non breeder
200	32	F	A	Medium	Pregnant

201	32	M	B	Good	Breeder
202	32	F	B	Good	Lacting
203	32	M	C	Bad	Breeder
204	32	M	B	Medium	Non breeder
205	32	F	A	Bad	Lacting
206	32	M	A	Good	Non breeder
207	32	F	A	Bad	Pregnant
208	32	F	B	Medium	Lacting
209	33	F	B	Good	Pregnant
210	33	F	A	Medium	Lacting
211	33	F	A	Bad	Lacting
212	33	M	B	Good	Breeder
213	33	M	B	Medium	Non breeder
214	33	F	A	Bad	Lacting
215	33	F	A	Good	Pregnant
216	34	F	A	Good	Lacting
217	34	F	B	Medium	Pregnant
218	34	M	B	Medium	Breeder
219	34	M	A	Bad	Non breeder
220	34	F	B	Good	Lacting
221	34	M	A	Good	Non breeder
222	35	F	A	Good	Pregnant
223	35	F	B	Medium	Lacting
224	35	F	B	Medium	Lacting
225	35	M	C	Good	Breeder
226	35	F	B	Bad	Lacting
227	35	M	B	Good	Non breeder
228	35	F	B	Medium	Lacting
229	36	F	B	Good	Lacting
230	36	F	A	Medium	Pregnant
231	36	F	A	Medium	Lacting
232	36	M	A	Good	Non breeder
233	36	F	B	Bad	Lacting
234	36	F	B	Medium	Pregnant
235	37	F	A	Good	Lacting
236	37	F	A	Bad	Pregnant
237	37	M	A	Good	Breeder
238	37	F	B	Medium	Non pregnant
239	37	F	B	Medium	Non pregnant
240	37	M	B	Bad	Non breeder
241	38	F	A	Bad	Lacting
242	38	M	B	Good	Breeder
243	38	M	B	Medium	Non breeder
244	38	F	B	Good	Pregnant
245	39	M	A	Good	Breeder
246	39	F	A	Good	Lacting
247	39	F	B	Bad	Pregnant
248	39	F	B	Medium	Lacting
249	39	F	B	Medium	Pregnant
250	40	F	B	Good	Pregnant

251	40	F	A	Bad	Lacting
252	40	F	A	Bad	Pregnant
253	40	M	B	Good	Breeder
254	40	M	B	Medium	Non breeder
255	40	F	B	Medium	Lacting
256	40	F	A	Medium	Lacting
257	41	F	A	Medium	Lacting
258	41	M	B	Medium	Non breeder
259	41	M	C	Good	Breeder
260	41	F	A	Good	Lacting
261	41	F	C	Medium	Pregnant
262	41	M	C	Good	Breeder
263	41	F	B	Bad	Lacting
264	42	F	B	Bad	Lacting
265	42	F	A	Good	Pregnant
266	42	M	A	Medium	Non breeder
267	42	F	B	Medium	Lacting
268	42	F	B	Good	Pregnant
269	42	F	B	Good	Lacting
270	42	M	A	Bad	Non breeder
271	42	M	B	Bad	Breeder
272	42	F	C	Good	Pregnant
273	43	M	A	Good	Non breeder
274	43	F	B	Bad	Lacting
275	43	M	A	Good	Non breeder
276	43	F	B	Bad	Lacting
277	43	F	B	Medium	Lacting
278	43	F	B	Medium	Pregnant
279	43	M	A	Good	Non breeder
280	44	M	B	V.good	Breeder
281	44	F	B	V.good	Pregnant
282	44	F	A	Medium	Lacting
283	44	M	A	Medium	Non breeder
284	44	F	B	Good	Lacting
285	44	F	C	Bad	Pregnant
286	45	F	C	Good	Lacting
287	45	F	B	Medium	Lacting
288	45	M	A	Bad	Non breeder
289	45	M	A	Good	Non breeder
290	45	F	A	Bad	Pregnant
291	45	F	B	Medium	Pregnant
292	45	F	B	V.good	Lacting
293	45	M	B	Good	Breeder
294	45	F	C	Medium	Lacting
295	46	F	B	Bad	Pregnant
296	46	M	B	Good	Breeder
297	46	F	A	Good	Lacting
298	46	F	A	V.good	Lacting
299	46	M	A	Medium	Non breeder
300	46	M	A	Medium	Non breeder

301	47	F	B	Medium	Lacting
302	47	F	B	Good	Lacting
303	47	M	A	Good	Breeder
304	47	F	A	Medium	Pregnant
305	47	F	B	Bad	Pregnant
306	47	F	B	Medium	Lacting
307	48	F	A	Medium	Lacting
308	48	F	B	Good	Lacting
309	48	M	A	Good	Non breeder
310	48	F	B	V.good	Lacting
311	48	F	B	Medium	Lacting
312	48	M	A	Bad	Non breeder
313	48	F	B	Medium	Lacting
314	49	F	A	Bad	Lacting
315	49	F	B	Good	Lacting
316	49	F	B	Medium	Lacting
317	49	F	B	Medium	Pregnant
318	49	M	A	Medium	Non breeder
319	50	F	B	Medium	Lacting
320	50	F	B	Good	Lacting
321	50	M	B	Good	Breeder
322	51	F	C	Bad	Pregnant
323	51	F	B	Good	Pregnant
324	51	M	B	Good	Breeder
325	51	F	A	V.good	Lacting
326	51	F	B	Medium	Lacting
327	51	F	C	Medium	Lacting
328	52	F	B	Good	Lacting
329	52	F	A	Good	Lacting
330	52	F	B	Bad	Lacting
331	52	F	B	Medium	Pregnant
332	52	F	C	Medium	Pregnant
333	52	F	B	Bad	Lacting
334	52	M	B	Good	Breeder
335	53	F	A	Good	Lacting
336	53	F	B	Good	Lacting
337	53	M	B	Medium	Breeder
338	53	F	B	V.good	Pregnant
339	53	F	A	Medium	Pregnant
340	53	M	A	Medium	Non breeder
341	53	M	A	Bad	Non breeder
342	54	F	B	Good	Lacting
343	54	F	A	Medium	Lacting
344	54	F	B	Medium	Lacting
345	54	F	B	Medium	Pregnant
346	55	F	B	Good	Lacting
347	55	F	A	Medium	Pregnant
348	55	F	B	Medium	Lacting
349	55	M	C	Medium	Breeder
350	56	F	C	Good	Lacting

351	56	F	A	Medium	Pregnant
352	56	F	B	Bad	Pregnant
353	56	M	A	Medium	Non breeder
354	56	M	B	Good	Breeder
355	57	F	C	Good	Lacting
356	57	F	B	Bad	Lacting
357	57	F	B	Medium	Pregnant
358	57	F	A	Medium	Pregnant
359	57	F	A	Bad	Lacting
360	57	M	B	Good	Breeder
361	58	F	A	Bad	Non pregnant
362	58	F	A	Good	Non pregnant
363	58	F	C	Medium	Lacting
364	58	M	B	Medium	Non breeder
365	58	M	B	Medium	Breeder
366	58	F	A	Medium	Non pregnant
367	59	F	B	Bad	Pregnant
368	59	F	A	Good	Lacting
369	59	M	A	Good	Non breeder
370	59	M	C	Medium	Breeder
371	59	F	A	V.good	Lacting
372	59	M	B	V.good	Breeder
373	59	F	A	Medium	Pregnant
374	60	F	C	Good	Pregnant
375	60	M	A	Good	Breeder
376	60	F	B	Bad	Lacting
377	60	M	B	Medium	Non breeder
378	60	F	A	V.good	Non pregnant
379	60	M	A	Medium	Non breeder
380	60	F	A	Bad	Non pregnant
381	60	F	B	V.good	Lacting
382	61	M	A	Good	Non breeder
383	61	M	A	Good	Non breeder
384	61	M	A	Medium	Non breeder
385	61	M	A	Medium	Non breeder
386	61	M	A	Medium	Non breeder
387	61	M	A	Medium	Non breeder
388	61	M	A	Medium	Non breeder
389	61	M	A	Medium	Non breeder
390	61	M	C	Bad	Non breeder
391	61	M	A	Bad	Non breeder
392	62	F	C	Bad	Pregnant
393	62	F	B	Good	Lacting
394	62	F	B	Medium	Lacting
395	62	M	C	Medium	Breeder
396	62	F	B	Medium	Lacting
397	62	F	A	Good	Non pregnant
398	62	M	C	Medium	Breeder
399	63	F	C	Good	Lacting
400	63	M	A	Good	Non breeder

401	63	F	A	Bad	Non pregnant
402	63	F	B	V.good	Pregnant
403	63	F	B	Medium	Pregnant
404	63	F	A	Medium	Pregnant
405	63	M	A	Bad	Non breeder
406	63	M	C	Medium	Breeder
407	63	F	A	Good	Non pregnant
408	63	F	B	Bad	Lacting
409	63	M	B	Medium	Non breeder
410	63	F	B	V.good	Lacting
411	63	F	C	Bad	Lacting
570	82	F	B	Medium	Lacting
571	82	F	B	Good	Lacting
572	82	F	B	Bad	Lacting
573	82	M	A	Good	Non breeder
574	82	M	A	Medium	Non breeder
575	82	M	B	Bad	Non breeder
576	83	F	B	Good	Pregnant
577	83	F	B	Good	Lacting
578	83	M	A	Medium	Non breeder
579	83	F	A	Medium	Non pregnant
580	83	F	A	Medium	Non pregnant
581	84	F	A	Medium	Lacting
582	84	F	B	Medium	Pregnant
583	84	F	B	Good	Pregnant
584	84	F	B	Good	Lacting
585	84	F	B	Medium	Lacting
586	85	F	C	Good	Lacting
587	85	M	B	Good	Breeder
588	85	F	A	V.good	Pregnant
589	85	F	B	V.good	Lacting
590	85	F	A	Medium	Non pregnant
591	85	F	B	Good	Lacting
592	86	F	B	Good	Lacting
593	86	M	B	Good	Breeder
594	86	F	B	Medium	Lacting
595	86	F	A	Medium	Pregnant
596	86	F	A	Good	Lacting
597	86	M	A	Good	Non breeder
598	87	M	A	Medium	Non breeder
599	87	F	A	Medium	Non pregnant
600	87	F	A	Medium	Non pregnant
601	87	M	A	Bad	Non breeder
602	88	M	A	Medium	Non breeder
603	88	F	A	Good	Pregnant
604	88	M	A	Good	Non breeder
605	88	F	B	Good	Lacting
606	88	M	C	Medium	Breeder
607	88	F	A	Good	Pregnant
608	89	F	A	Bad	Lacting

609	89	F	B	V.good	Lacting
610	89	M	B	V.good	Breeder
611	89	F	C	Good	Pregnant
612	89	F	C	Bad	Lacting
613	89	F	C	Good	Lacting
614	89	F	B	Good	Pregnant
615	89	F	B	Medium	Lacting
616	90	F	C	V.good	Pregnant
617	90	M	B	V.good	Breeder
618	90	M	A	Medium	Non breeder
619	90	F	B	Good	Lacting
620	90	F	B	Good	Lacting
621	90	F	B	Good	Lacting

M – Male; F – Female; A – Abortion; NA - No Abortion;
A- animals under 2 years of age; B - animals are 2-5 years old
C - animals over 5 years of age
Int – Intensive; Sint – Semi-intensive; Ex – Extensive; SE – Semi-extensiv
V.good; Very good

Type of production	Management system	Herd size	Pasture	Pasture freque
Mixed	S.int	(+31)	Yes	Sporadic
Mixed	S.int	(+31)	Yes	Sporadic
Mixed	S.int	(+31)	Yes	Sporadic
Mixed	S.int	(+31)	Yes	Sporadic
Mixed	S.int	(+31)	Yes	Sporadic
Mixed	S.int	(+31)	Yes	Sporadic
Mixed	S.int	(+31)	Yes	Sporadic
Mixed	S.int	(+31)	Yes	Sporadic
Mixed	S.int	(+31)	Yes	Sporadic
Mixed	S.int	(+31)	Yes	Sporadic
Mixed	S.int	(+31)	Yes	Sporadic
Mixed	S.int	(+31)	Yes	Sporadic
Milk	S.int	(1-10)	Yes	Seasonal
Milk	S.int	(1-10)	Yes	Seasonal
Milk	S.int	(1-10)	Yes	Seasonal
Milk	S.int	(1-10)	Yes	Seasonal
Milk	S.int	(1-10)	Yes	Seasonal
Milk	SE	(11-30)	Yes	Seasonal
Milk	SE	(11-30)	Yes	Seasonal
Milk	SE	(11-30)	Yes	Seasonal
Milk	SE	(11-30)	Yes	Seasonal
Milk	SE	(11-30)	Yes	Seasonal
Milk	SE	(11-30)	Yes	Seasonal
Milk	SE	(11-30)	Yes	Seasonal
Milk	SE	(11-30)	Yes	Seasonal
Mixed	SE	(11-30)	Yes	Seasonal
Mixed	SE	(11-30)	Yes	Seasonal
Mixed	SE	(11-30)	Yes	Seasonal
Mixed	SE	(11-30)	Yes	Seasonal
Mixed	SE	(11-30)	Yes	Seasonal
Mixed	SE	(11-30)	Yes	Seasonal
Mixed	SE	(11-30)	Yes	Seasonal
Meat	Int	(1-10)	No	Never
Meat	Int	(1-10)	No	Never
Meat	Int	(1-10)	No	Never
Meat	Int	(1-10)	No	Never
Milk	S.int	(11-30)	Yes	Seasonal
Milk	S.int	(11-30)	Yes	Seasonal
Milk	S.int	(11-30)	Yes	Seasonal
Milk	S.int	(11-30)	Yes	Seasonal
Milk	S.int	(11-30)	Yes	Seasonal
Milk	S.int	(11-30)	Yes	Seasonal
Milk	S.int	(11-30)	Yes	Seasonal
Milk	S.int	(11-30)	Yes	Seasonal
Milk	S.int	(11-30)	Yes	Seasonal
Mixed	S.int	(11-30)	Yes	Sporadic
Mixed	S.int	(11-30)	Yes	Sporadic

Mixed	S.int	(11-30)	Yes	Sporadic
Mixed	S.int	(11-30)	Yes	Sporadic
Mixed	S.int	(11-30)	Yes	Sporadic
Mixed	S.int	(11-30)	Yes	Sporadic
Mixed	S.int	(11-30)	Yes	Sporadic
Mixed	S.int	(11-30)	Yes	Sporadic
Mixed	S.int	(11-30)	Yes	Sporadic
Mixed	S.int	(11-30)	Yes	Sporadic
Mixed	SE	(11-30)	Yes	Seasonal
Mixed	SE	(11-30)	Yes	Seasonal
Mixed	SE	(11-30)	Yes	Seasonal
Mixed	SE	(11-30)	Yes	Seasonal
Mixed	SE	(11-30)	Yes	Seasonal
Mixed	SE	(11-30)	Yes	Seasonal
Mixed	SE	(11-30)	Yes	Seasonal
Mixed	SE	(11-30)	Yes	Seasonal
Mixed	SE	(11-30)	Yes	Seasonal
Mixed	SE	(11-30)	Yes	Seasonal
Mixed	SE	(11-30)	Yes	Seasonal
Meat	SE	(1-10)	Yes	Seasonal
Meat	SE	(1-10)	Yes	Seasonal
Meat	SE	(1-10)	Yes	Seasonal
Meat	SE	(1-10)	Yes	Seasonal
Meat	SE	(1-10)	Yes	Seasonal
Mixed	S.int	(11-30)	Yes	Sporadic
Mixed	S.int	(11-30)	Yes	Sporadic
Mixed	S.int	(11-30)	Yes	Sporadic
Mixed	S.int	(11-30)	Yes	Sporadic
Mixed	S.int	(11-30)	Yes	Sporadic
Mixed	S.int	(11-30)	Yes	Sporadic
Mixed	S.int	(11-30)	Yes	Sporadic
Milk	S.int	(1-10)	Yes	Sporadic
Milk	S.int	(1-10)	Yes	Sporadic
Milk	S.int	(1-10)	Yes	Sporadic
Milk	S.int	(1-10)	Yes	Sporadic
Milk	S.int	(1-10)	Yes	Sporadic
Milk	S.int	(1-10)	Yes	Sporadic
Milk	S.int	(1-10)	Yes	Sporadic
Milk	S.int	(1-10)	Yes	Sporadic
Meat	SE	(1-10)	Yes	Seasonal
Meat	SE	(1-10)	Yes	Seasonal
Meat	SE	(1-10)	Yes	Seasonal
Meat	SE	(1-10)	Yes	Seasonal
Meat	SE	(1-10)	Yes	Seasonal
Meat	SE	(1-10)	Yes	Seasonal
Meat	SE	(1-10)	Yes	Seasonal
Meat	SE	(1-10)	Yes	Seasonal
Milk	SE	(1-10)	Yes	Seasonal
Milk	SE	(1-10)	Yes	Seasonal
Milk	SE	(1-10)	Yes	Seasonal

Mixed	S.int	(11-30)	Yes	Sporadic
Mixed	S.int	(11-30)	Yes	Sporadic
Mixed	S.int	(11-30)	Yes	Sporadic
Mixed	S.int	(11-30)	Yes	Sporadic
Mixed	S.int	(11-30)	Yes	Sporadic
Mixed	S.int	(11-30)	Yes	Sporadic
Mixed	S.int	(11-30)	Yes	Sporadic
Mixed	S.int	(11-30)	Yes	Sporadic
Milk	S.int	(1-10)	Yes	Sporadic
Milk	S.int	(1-10)	Yes	Sporadic
Milk	S.int	(1-10)	Yes	Sporadic
Milk	S.int	(1-10)	Yes	Sporadic
Milk	S.int	(1-10)	Yes	Sporadic
Mixed	S.int	(1-10)	Yes	Seasonal
Mixed	S.int	(1-10)	Yes	Seasonal
Mixed	S.int	(1-10)	Yes	Seasonal
Mixed	S.int	(1-10)	Yes	Seasonal
Mixed	S.int	(1-10)	Yes	Seasonal
Milk	S.int	(1-10)	Yes	Sporadic
Milk	S.int	(1-10)	Yes	Sporadic
Milk	S.int	(1-10)	Yes	Sporadic
Milk	S.int	(1-10)	Yes	Sporadic
Milk	S.int	(1-10)	Yes	Sporadic
Milk	Int	(1-10)	No	Never
Milk	Int	(1-10)	No	Never
Milk	Int	(1-10)	No	Never
Milk	Int	(1-10)	No	Never
Milk	Int	(1-10)	No	Never
Milk	Int	(1-10)	No	Never
Meat	SE	(1-10)	Yes	Seasonal
Meat	SE	(1-10)	Yes	Seasonal
Meat	SE	(1-10)	Yes	Seasonal
Meat	SE	(1-10)	Yes	Seasonal
Meat	SE	(1-10)	Yes	Seasonal
Milk	S.int	(1-10)	Yes	Sporadic
Milk	S.int	(1-10)	Yes	Sporadic
Milk	S.int	(1-10)	Yes	Sporadic
Milk	S.int	(1-10)	Yes	Sporadic
Milk	S.int	(1-10)	Yes	Sporadic
Milk	S.int	(1-10)	Yes	Sporadic
Meat	S.int	(11-30)	Yes	Sporadic
Meat	S.int	(11-30)	Yes	Sporadic
Meat	S.int	(11-30)	Yes	Sporadic
Meat	S.int	(11-30)	Yes	Sporadic
Meat	S.int	(11-30)	Yes	Sporadic
Milk	S.int	(1-10)	Yes	Seasonal
Milk	S.int	(1-10)	Yes	Seasonal
Milk	S.int	(1-10)	Yes	Seasonal
Milk	S.int	(1-10)	Yes	Seasonal
Milk	S.int	(1-10)	Yes	Seasonal

Milk	S.int	(1-10)	Yes	Seasonal
Milk	S.int	(1-10)	Yes	Seasonal
Meat	SE	(1-10)	Yes	Seasonal
Meat	SE	(1-10)	Yes	Seasonal
Meat	SE	(1-10)	Yes	Seasonal
Meat	SE	(1-10)	Yes	Seasonal
Milk	S.int	(1-10)	Yes	Sporadic
Milk	S.int	(1-10)	Yes	Sporadic
Milk	S.int	(1-10)	Yes	Sporadic
Milk	S.int	(1-10)	Yes	Sporadic
Milk	SE	(1-10)	Yes	Seasonal
Milk	SE	(1-10)	Yes	Seasonal
Milk	SE	(1-10)	Yes	Seasonal
Milk	SE	(1-10)	Yes	Seasonal
Mixed	S.int	(11-30)	Yes	Sporadic
Mixed	S.int	(11-30)	Yes	Sporadic
Mixed	S.int	(11-30)	Yes	Sporadic
Mixed	S.int	(11-30)	Yes	Sporadic
Mixed	S.int	(11-30)	Yes	Sporadic
Mixed	S.int	(11-30)	Yes	Sporadic
Mixed	S.int	(11-30)	Yes	Sporadic
Mixed	S.int	(11-30)	Yes	Sporadic
Mixed	S.int	(11-30)	Yes	Sporadic
Meat	S.int	(11-30)	Yes	Sporadic
Meat	S.int	(11-30)	Yes	Sporadic
Meat	S.int	(11-30)	Yes	Sporadic
Meat	S.int	(11-30)	Yes	Sporadic
Meat	S.int	(11-30)	Yes	Sporadic
Meat	S.int	(11-30)	Yes	Sporadic
Milk	SE	(1-10)	Yes	Seasonal
Milk	SE	(1-10)	Yes	Seasonal
Milk	SE	(1-10)	Yes	Seasonal
Milk	SE	(1-10)	Yes	Seasonal
Milk	SE	(1-10)	Yes	Seasonal
Mixed	SE	(11-30)	Yes	Seasonal
Mixed	SE	(11-30)	Yes	Seasonal
Mixed	SE	(11-30)	Yes	Seasonal
Mixed	SE	(11-30)	Yes	Seasonal
Mixed	SE	(11-30)	Yes	Seasonal
Mixed	SE	(11-30)	Yes	Seasonal
Milk	S.int	(1-10)	Yes	Sporadic
Milk	S.int	(1-10)	Yes	Sporadic
Milk	S.int	(1-10)	Yes	Sporadic
Milk	S.int	(1-10)	Yes	Sporadic
Milk	S.int	(1-10)	Yes	Sporadic
Meat	SE	(11-30)	Yes	Sporadic
Meat	SE	(11-30)	Yes	Sporadic
Meat	SE	(11-30)	Yes	Sporadic
Meat	SE	(11-30)	Yes	Sporadic
Mixed	S.int	(11-30)	Yes	Seasonal

Mixed	S.int	(11-30)	Yes	Seasonal
Mixed	S.int	(11-30)	Yes	Seasonal
Mixed	S.int	(11-30)	Yes	Seasonal
Mixed	S.int	(11-30)	Yes	Seasonal
Mixed	S.int	(11-30)	Yes	Seasonal
Mixed	S.int	(11-30)	Yes	Seasonal
Mixed	S.int	(11-30)	Yes	Seasonal
Mixed	S.int	(11-30)	Yes	Seasonal
Milk	S.int	(11-30)	Yes	Seasonal
Milk	S.int	(11-30)	Yes	Seasonal
Milk	S.int	(11-30)	Yes	Seasonal
Milk	S.int	(11-30)	Yes	Seasonal
Milk	S.int	(11-30)	Yes	Seasonal
Milk	S.int	(11-30)	Yes	Seasonal
Milk	S.int	(11-30)	Yes	Seasonal
Mixed	SE	(1-10)	Yes	Seasonal
Mixed	SE	(1-10)	Yes	Seasonal
Mixed	SE	(1-10)	Yes	Seasonal
Mixed	SE	(1-10)	Yes	Seasonal
Mixed	SE	(1-10)	Yes	Seasonal
Mixed	SE	(1-10)	Yes	Seasonal
Milk	S.int	(1-10)	Yes	Sporadic
Milk	S.int	(1-10)	Yes	Sporadic
Milk	S.int	(1-10)	Yes	Sporadic
Milk	S.int	(1-10)	Yes	Sporadic
Milk	S.int	(1-10)	Yes	Sporadic
Milk	S.int	(1-10)	Yes	Sporadic
Milk	S.int	(1-10)	Yes	Sporadic
Mixed	S.int	(11-30)	Yes	Sporadic
Mixed	S.int	(11-30)	Yes	Sporadic
Mixed	S.int	(11-30)	Yes	Sporadic
Mixed	S.int	(11-30)	Yes	Sporadic
Mixed	S.int	(11-30)	Yes	Sporadic
Mixed	S.int	(11-30)	Yes	Sporadic
Meat	SE	(11-30)	Yes	Seasonal
Meat	SE	(11-30)	Yes	Seasonal
Meat	SE	(11-30)	Yes	Seasonal
Meat	SE	(11-30)	Yes	Seasonal
Meat	SE	(11-30)	Yes	Seasonal
Meat	SE	(11-30)	Yes	Seasonal
Mixed	S.int	(1-10)	Yes	Sporadic
Mixed	S.int	(1-10)	Yes	Sporadic
Mixed	S.int	(1-10)	Yes	Sporadic
Mixed	S.int	(1-10)	Yes	Sporadic
Mixed	SE	(11-30)	Yes	Seasonal
Mixed	SE	(11-30)	Yes	Seasonal
Mixed	SE	(11-30)	Yes	Seasonal
Mixed	SE	(11-30)	Yes	Seasonal
Mixed	SE	(11-30)	Yes	Seasonal
Milk	Int	(11-30)	No	Never

Milk	Int	(11-30)	No	Never
Milk	Int	(11-30)	No	Never
Milk	Int	(11-30)	No	Never
Milk	Int	(11-30)	No	Never
Milk	Int	(11-30)	No	Never
Milk	Int	(11-30)	No	Never
Mixed	S.int	(1-10)	Yes	Seasonal
Mixed	S.int	(1-10)	Yes	Seasonal
Mixed	S.int	(1-10)	Yes	Seasonal
Mixed	S.int	(1-10)	Yes	Seasonal
Mixed	S.int	(1-10)	Yes	Seasonal
Mixed	S.int	(1-10)	Yes	Seasonal
Mixed	S.int	(1-10)	Yes	Seasonal
Mixed	S.int	(11-30)	Yes	Sporadic
Mixed	S.int	(11-30)	Yes	Sporadic
Mixed	S.int	(11-30)	Yes	Sporadic
Mixed	S.int	(11-30)	Yes	Sporadic
Mixed	S.int	(11-30)	Yes	Sporadic
Mixed	S.int	(11-30)	Yes	Sporadic
Mixed	S.int	(11-30)	Yes	Sporadic
Mixed	S.int	(11-30)	Yes	Sporadic
Mixed	S.int	(11-30)	Yes	Sporadic
Mixed	SE	(1-10)	Yes	Frequent
Mixed	SE	(1-10)	Yes	Frequent
Mixed	SE	(1-10)	Yes	Frequent
Mixed	SE	(1-10)	Yes	Frequent
Mixed	SE	(1-10)	Yes	Frequent
Mixed	SE	(1-10)	Yes	Frequent
Mixed	SE	(1-10)	Yes	Frequent
Mixed	SE	(1-10)	Yes	Seasonal
Mixed	SE	(1-10)	Yes	Seasonal
Mixed	SE	(1-10)	Yes	Seasonal
Mixed	SE	(1-10)	Yes	Seasonal
Mixed	SE	(1-10)	Yes	Seasonal
Mixed	SE	(1-10)	Yes	Seasonal
Mixed	S.int	(11-30)	Yes	Sporadic
Mixed	S.int	(11-30)	Yes	Sporadic
Mixed	S.int	(11-30)	Yes	Sporadic
Mixed	S.int	(11-30)	Yes	Sporadic
Mixed	S.int	(11-30)	Yes	Sporadic
Mixed	S.int	(11-30)	Yes	Sporadic
Mixed	S.int	(11-30)	Yes	Sporadic
Mixed	S.int	(11-30)	Yes	Sporadic
Mixed	S.int	(11-30)	Yes	Sporadic
Meat	S.int	(11-30)	Yes	Seasonal
Meat	S.int	(11-30)	Yes	Seasonal
Meat	S.int	(11-30)	Yes	Seasonal
Meat	S.int	(11-30)	Yes	Seasonal
Meat	S.int	(11-30)	Yes	Seasonal
Meat	S.int	(11-30)	Yes	Seasonal

Milk	SE	(1-10)	Yes	Seasonal
Milk	SE	(1-10)	Yes	Seasonal
Milk	SE	(1-10)	Yes	Seasonal
Milk	SE	(1-10)	Yes	Seasonal
Milk	SE	(1-10)	Yes	Seasonal
Milk	SE	(1-10)	Yes	Seasonal
Milk	S.int	(1-10)	Yes	Sporadic
Milk	S.int	(1-10)	Yes	Sporadic
Milk	S.int	(1-10)	Yes	Sporadic
Milk	S.int	(1-10)	Yes	Sporadic
Milk	S.int	(1-10)	Yes	Sporadic
Milk	S.int	(1-10)	Yes	Sporadic
Milk	S.int	(1-10)	Yes	Sporadic
Mixed	S.int	(1-10)	Yes	Seasonal
Mixed	S.int	(1-10)	Yes	Seasonal
Mixed	S.int	(1-10)	Yes	Seasonal
Mixed	S.int	(1-10)	Yes	Seasonal
Mixed	S.int	(1-10)	Yes	Seasonal
Milk	S.int	(1-10)	Yes	Sporadic
Milk	S.int	(1-10)	Yes	Sporadic
Milk	S.int	(1-10)	Yes	Sporadic
Milk	SE	(1-10)	Yes	Seasonal
Milk	SE	(1-10)	Yes	Seasonal
Milk	SE	(1-10)	Yes	Seasonal
Milk	SE	(1-10)	Yes	Seasonal
Milk	SE	(1-10)	Yes	Seasonal
Milk	SE	(1-10)	Yes	Seasonal
Milk	S.int	(11-30)	Yes	Seasonal
Milk	S.int	(11-30)	Yes	Seasonal
Milk	S.int	(11-30)	Yes	Seasonal
Milk	S.int	(11-30)	Yes	Seasonal
Milk	S.int	(11-30)	Yes	Seasonal
Milk	S.int	(11-30)	Yes	Seasonal
Milk	S.int	(11-30)	Yes	Seasonal
Mixed	SE	(11-30)	Yes	Seasonal
Mixed	SE	(11-30)	Yes	Seasonal
Mixed	SE	(11-30)	Yes	Seasonal
Mixed	SE	(11-30)	Yes	Seasonal
Mixed	SE	(11-30)	Yes	Seasonal
Mixed	SE	(11-30)	Yes	Seasonal
Mixed	SE	(11-30)	Yes	Seasonal
Milk	S.int	(1-10)	Yes	Seasonal
Milk	S.int	(1-10)	Yes	Seasonal
Milk	S.int	(1-10)	Yes	Seasonal
Milk	S.int	(1-10)	Yes	Seasonal
Milk	SE	(1-10)	Yes	Frequent
Milk	SE	(1-10)	Yes	Frequent
Milk	SE	(1-10)	Yes	Frequent
Milk	SE	(1-10)	Yes	Frequent
Meat	S.int	(1-10)	Yes	Sporadic

Meat	S.int	(1-10)	Yes	Sporadic
Meat	S.int	(1-10)	Yes	Sporadic
Meat	S.int	(1-10)	Yes	Sporadic
Meat	S.int	(1-10)	Yes	Sporadic
Milk	SE	(11-30)	Yes	Seasonal
Milk	SE	(11-30)	Yes	Seasonal
Milk	SE	(11-30)	Yes	Seasonal
Milk	SE	(11-30)	Yes	Seasonal
Milk	SE	(11-30)	Yes	Seasonal
Milk	SE	(11-30)	Yes	Seasonal
Mixed	S.int	(11-30)	Yes	Sporadic
Mixed	S.int	(11-30)	Yes	Sporadic
Mixed	S.int	(11-30)	Yes	Sporadic
Mixed	S.int	(11-30)	Yes	Sporadic
Mixed	S.int	(11-30)	Yes	Sporadic
Mixed	S.int	(11-30)	Yes	Sporadic
Mixed	S.int	(+31)	Yes	Seasonal
Mixed	S.int	(+31)	Yes	Seasonal
Mixed	S.int	(+31)	Yes	Seasonal
Mixed	S.int	(+31)	Yes	Seasonal
Mixed	S.int	(+31)	Yes	Seasonal
Mixed	S.int	(+31)	Yes	Seasonal
Mixed	S.int	(+31)	Yes	Seasonal
Milk	SE	(11-30)	Yes	Seasonal
Milk	SE	(11-30)	Yes	Seasonal
Milk	SE	(11-30)	Yes	Seasonal
Milk	SE	(11-30)	Yes	Seasonal
Milk	SE	(11-30)	Yes	Seasonal
Milk	SE	(11-30)	Yes	Seasonal
Milk	SE	(11-30)	Yes	Seasonal
Milk	SE	(11-30)	Yes	Seasonal
Meat	Int	(11-30)	No	Never
Meat	Int	(11-30)	No	Never
Meat	Int	(11-30)	No	Never
Meat	Int	(11-30)	No	Never
Meat	Int	(11-30)	No	Never
Meat	Int	(11-30)	No	Never
Meat	Int	(11-30)	No	Never
Meat	Int	(11-30)	No	Never
Meat	Int	(11-30)	No	Never
Meat	Int	(11-30)	No	Never
Mixed	Ex	(11-30)	Yes	Frequent
Mixed	Ex	(11-30)	Yes	Frequent
Mixed	Ex	(1-10)	Yes	Frequent
Mixed	Ex	(1-10)	Yes	Frequent
Mixed	Ex	(1-10)	Yes	Frequent
Mixed	Ex	(1-10)	Yes	Frequent
Mixed	Ex	(11-30)	Yes	Frequent
Mixed	Ex	(11-30)	Yes	Frequent
Mixed	Ex	(11-30)	Yes	Frequent

Mixed	Ex	(11-30)	Yes	Frequent
Mixed	Ex	(11-30)	Yes	Frequent
Mixed	Ex	(11-30)	Yes	Frequent
Mixed	Ex	(11-30)	Yes	Frequent
Mixed	Ex	(11-30)	Yes	Frequent
Mixed	Ex	(11-30)	Yes	Frequent
Mixed	Ex	(11-30)	Yes	Frequent
Mixed	Ex	(11-30)	Yes	Frequent
Mixed	Ex	(11-30)	Yes	Frequent
Mixed	Ex	(11-30)	Yes	Frequent
Mixed	Ex	(11-30)	Yes	Frequent
Mixed	S.int	(1-10)	Yes	Seasonal
Mixed	S.int	(1-10)	Yes	Seasonal
Mixed	S.int	(1-10)	Yes	Seasonal
Mixed	S.int	(1-10)	Yes	Seasonal
Mixed	S.int	(1-10)	Yes	Seasonal
Mixed	S.int	(1-10)	Yes	Seasonal
Mixed	S.int	(1-10)	Yes	Frequent
Mixed	S.int	(1-10)	Yes	Frequent
Mixed	S.int	(1-10)	Yes	Frequent
Mixed	S.int	(1-10)	Yes	Frequent
Mixed	S.int	(1-10)	Yes	Frequent
Milk	SE	(1-10)	Yes	Seasonal
Milk	SE	(1-10)	Yes	Seasonal
Milk	SE	(1-10)	Yes	Seasonal
Milk	SE	(1-10)	Yes	Seasonal
Milk	SE	(1-10)	Yes	Seasonal
Milk	S.int	(11-30)	Yes	Seasonal
Milk	S.int	(11-30)	Yes	Seasonal
Milk	S.int	(11-30)	Yes	Seasonal
Milk	S.int	(11-30)	Yes	Seasonal
Milk	S.int	(11-30)	Yes	Seasonal
Milk	S.int	(11-30)	Yes	Seasonal
Mixed	SE	(11-30)	Yes	Frequent
Mixed	SE	(11-30)	Yes	Frequent
Mixed	SE	(11-30)	Yes	Frequent
Mixed	SE	(11-30)	Yes	Frequent
Mixed	SE	(11-30)	Yes	Frequent
Mixed	SE	(11-30)	Yes	Frequent
Mixed	Ex	(1-10)	Yes	Frequent
Mixed	Ex	(1-10)	Yes	Frequent
Mixed	Ex	(1-10)	Yes	Frequent
Mixed	Ex	(1-10)	Yes	Frequent
Mixed	S.int	(1-10)	Yes	Seasonal
Mixed	S.int	(1-10)	Yes	Seasonal
Mixed	S.int	(1-10)	Yes	Seasonal
Mixed	S.int	(1-10)	Yes	Seasonal
Mixed	S.int	(1-10)	Yes	Seasonal
Mixed	S.int	(1-10)	Yes	Seasonal
Milk	S.int	(1-10)	Yes	Seasonal

Milk	S.int	(1-10)	Yes	Seasonal
Milk	S.int	(1-10)	Yes	Seasonal
Milk	S.int	(1-10)	Yes	Seasonal
Milk	S.int	(1-10)	Yes	Seasonal
Milk	S.int	(1-10)	Yes	Seasonal
Milk	S.int	(1-10)	Yes	Seasonal
Milk	S.int	(1-10)	Yes	Seasonal
Milk	S.int	(11-30)	Yes	Seasonal
Milk	S.int	(11-30)	Yes	Seasonal
Milk	S.int	(11-30)	Yes	Seasonal
Milk	S.int	(11-30)	Yes	Seasonal
Milk	S.int	(11-30)	Yes	Seasonal
Milk	S.int	(11-30)	Yes	Seasonal

Type of pasture area	Size of pasture area	Common pastures	Transhumance
Hill	from11 to 50H	No	No
Hill	from11 to 50H	No	No
Hill	from11 to 50H	No	No
Hill	from11 to 50H	No	No
Hill	from11 to 50H	No	No
Hill	from11 to 50H	No	No
Hill	from11 to 50H	No	No
Hill	from11 to 50H	No	No
Hill	from11 to 50H	No	No
Hill	from11 to 50H	No	No
Hill	from11 to 50H	No	No
Hill	from11 to 50H	No	No
Hill	10H	Yes	No
Hill	10H	Yes	No
Hill	10H	Yes	No
Hill	10H	Yes	No
Hill	10H	Yes	No
Plain	from11 to 50H	Yes	No
Plain	from11 to 50H	Yes	No
Plain	from11 to 50H	Yes	No
Plain	from11 to 50H	Yes	No
Plain	from11 to 50H	Yes	No
Plain	from11 to 50H	Yes	No
Plain	from11 to 50H	Yes	No
Plain	from11 to 50H	Yes	No
Hill	10H	Yes	No
Hill	10H	Yes	No
Hill	10H	Yes	No
Hill	10H	Yes	No
Hill	10H	Yes	No
Hill	10H	Yes	No
Hill	10H	Yes	No
			No
			No
			No
			No
Hill	from11 to 50H	Yes	No
Hill	from11 to 50H	Yes	No
Hill	from11 to 50H	Yes	No
Hill	from11 to 50H	Yes	No
Hill	from11 to 50H	Yes	No
Hill	from11 to 50H	Yes	No
Hill	from11 to 50H	Yes	No
Hill	from11 to 50H	Yes	No
Hill	from11 to 50H	Yes	No
Plain	10H	No	No
Plain	10H	No	No

Plain	10H	No	No
Plain	10H	No	No
Plain	10H	No	No
Plain	10H	No	No
Plain	10H	No	No
Plain	10H	No	No
Plain	10H	No	No
Plain	10H	No	No
Plain	from11 to 50H	Yes	No
Plain	from11 to 50H	Yes	No
Plain	from11 to 50H	Yes	No
Plain	from11 to 50H	Yes	No
Plain	from11 to 50H	Yes	No
Plain	from11 to 50H	Yes	No
Plain	from11 to 50H	Yes	No
Plain	from11 to 50H	Yes	No
Plain	from11 to 50H	Yes	No
Plain	from11 to 50H	Yes	No
Plain	from11 to 50H	Yes	No
Hill	10H	No	No
Hill	10H	No	No
Hill	10H	No	No
Hill	10H	No	No
Hill	10H	No	No
Hill	10H	No	No
Hill	10H	No	No
Hill	10H	No	No
Hill	10H	No	No
Hill	10H	No	No
Hill	10H	No	No
Hill	10H	No	No
Plain	10H	Yes	No
Plain	10H	Yes	No
Plain	10H	Yes	No
Plain	10H	Yes	No
Plain	10H	Yes	No
Plain	10H	Yes	No
Plain	10H	Yes	No
Plain	10H	Yes	No
Hill	from11 to 50H	Yes	No
Hill	from11 to 50H	Yes	No
Hill	from11 to 50H	Yes	No
Hill	from11 to 50H	Yes	No
Hill	from11 to 50H	Yes	No
Hill	from11 to 50H	Yes	No
Hill	from11 to 50H	Yes	No
Hill	from11 to 50H	Yes	No
Plain	10H	No	No
Plain	10H	No	No
Plain	10H	No	No

Plain	10H	No	No
Plain	10H	No	No
Plain	10H	No	No
Plain	10H	No	No
Plain	10H	No	No
Plain	10H	No	No
Plain	10H	No	No
Plain	10H	No	No
Hill	10H	Yes	No
Hill	10H	Yes	No
Hill	10H	Yes	No
Hill	10H	Yes	No
Hill	10H	Yes	No
Plain	from11 to 50H	Yes	No
Plain	from11 to 50H	Yes	No
Plain	from11 to 50H	Yes	No
Plain	from11 to 50H	Yes	No
Plain	from11 to 50H	Yes	No
Hill	10H	No	No
Hill	10H	No	No
Hill	10H	No	No
Hill	10H	No	No
Hill	10H	No	No
			No
			No
			No
			No
			No
			No
Hill	10H	Yes	No
Hill	10H	Yes	No
Hill	10H	Yes	No
Hill	10H	Yes	No
Hill	10H	Yes	No
Plain	10H	Yes	No
Plain	10H	Yes	No
Plain	10H	Yes	No
Plain	10H	Yes	No
Plain	10H	Yes	No
Plain	10H	Yes	No
Plain	from11 to 50H	Yes	No
Plain	from11 to 50H	Yes	No
Plain	from11 to 50H	Yes	No
Plain	from11 to 50H	Yes	No
Plain	from11 to 50H	Yes	No
Hill	10H	No	No
Hill	10H	No	No
Hill	10H	No	No
Hill	10H	No	No
Hill	10H	No	No

Hill	10H	No	No
Hill	10H	No	No
Hill	10H	No	No
Hill	10H	No	No
Hill	10H	No	No
Hill	10H	No	No
Plain	from11 to 50H	Yes	No
Plain	from11 to 50H	Yes	No
Plain	from11 to 50H	Yes	No
Plain	from11 to 50H	Yes	No
Plain	from11 to 50H	No	No
Plain	from11 to 50H	No	No
Plain	from11 to 50H	No	No
Plain	from11 to 50H	No	No
Hill	from11 to 50H	Yes	No
Hill	from11 to 50H	Yes	No
Hill	from11 to 50H	Yes	No
Hill	from11 to 50H	Yes	No
Hill	from11 to 50H	Yes	No
Hill	from11 to 50H	Yes	No
Hill	from11 to 50H	Yes	No
Hill	from11 to 50H	Yes	No
Hill	from11 to 50H	Yes	No
Plain	10H	No	No
Plain	10H	No	No
Plain	10H	No	No
Plain	10H	No	No
Plain	10H	No	No
Plain	10H	No	No
Hill	from11 to 50H	Yes	No
Hill	from11 to 50H	Yes	No
Hill	from11 to 50H	Yes	No
Hill	from11 to 50H	Yes	No
Hill	from11 to 50H	Yes	No
Plain	from11 to 50H	Yes	Yes
Plain	from11 to 50H	Yes	Yes
Plain	from11 to 50H	Yes	Yes
Plain	from11 to 50H	Yes	Yes
Plain	from11 to 50H	Yes	Yes
Plain	from11 to 50H	Yes	Yes
Hill	10H	No	No
Hill	10H	No	No
Hill	10H	No	No
Hill	10H	No	No
Hill	10H	No	No
Hill	from11 to 50H	No	No
Hill	from11 to 50H	No	No
Hill	from11 to 50H	No	No
Hill	from11 to 50H	No	No
Plain	from11 to 50H	Yes	No

Plain	from11 to 50H	Yes	No
Plain	from11 to 50H	Yes	No
Plain	from11 to 50H	Yes	No
Plain	from11 to 50H	Yes	No
Plain	from11 to 50H	Yes	No
Plain	from11 to 50H	Yes	No
Plain	from11 to 50H	Yes	No
Plain	from11 to 50H	Yes	No
Hill	10H	No	No
Hill	10H	No	No
Hill	10H	No	No
Hill	10H	No	No
Hill	10H	No	No
Hill	10H	No	No
Hill	10H	No	No
Mountain	plus50H	Yes	Yes
Mountain	plus50H	Yes	Yes
Mountain	plus50H	Yes	Yes
Mountain	plus50H	Yes	Yes
Mountain	plus50H	Yes	Yes
Mountain	plus50H	Yes	Yes
Hill	from11 to 50H	No	No
Hill	from11 to 50H	No	No
Hill	from11 to 50H	No	No
Hill	from11 to 50H	No	No
Hill	from11 to 50H	No	No
Hill	from11 to 50H	No	No
Hill	from11 to 50H	No	No
Hill	from11 to 50H	Yes	No
Hill	from11 to 50H	Yes	No
Hill	from11 to 50H	Yes	No
Hill	from11 to 50H	Yes	No
Hill	from11 to 50H	Yes	No
Hill	from11 to 50H	Yes	No
Plain	10H	No	No
Plain	10H	No	No
Plain	10H	No	No
Plain	10H	No	No
Plain	10H	No	No
Plain	10H	No	No
Hill	10H	Yes	No
Hill	10H	Yes	No
Hill	10H	Yes	No
Hill	10H	Yes	No
Plain	from11 to 50H	Yes	No
Plain	from11 to 50H	Yes	No
Plain	from11 to 50H	Yes	No
Plain	from11 to 50H	Yes	No
Plain	from11 to 50H	Yes	No
			No

			No
			No
			No
			No
			No
			No
Hill	10H	No	No
Hill	10H	No	No
Hill	10H	No	No
Hill	10H	No	No
Hill	10H	No	No
Hill	10H	No	No
Hill	10H	No	No
Hill	10H	Yes	No
Hill	10H	Yes	No
Hill	10H	Yes	No
Hill	10H	Yes	No
Hill	10H	Yes	No
Hill	10H	Yes	No
Hill	10H	Yes	No
Hill	10H	Yes	No
Hill	10H	Yes	No
Plain	from11 to 50H	Yes	No
Plain	from11 to 50H	Yes	No
Plain	from11 to 50H	Yes	No
Plain	from11 to 50H	Yes	No
Plain	from11 to 50H	Yes	No
Plain	from11 to 50H	Yes	No
Plain	from11 to 50H	Yes	No
Hill	from11 to 50H	Yes	No
Hill	from11 to 50H	Yes	No
Hill	from11 to 50H	Yes	No
Hill	from11 to 50H	Yes	No
Hill	from11 to 50H	Yes	No
Hill	from11 to 50H	Yes	No
Plain	from11 to 50H	No	No
Plain	from11 to 50H	No	No
Plain	from11 to 50H	No	No
Plain	from11 to 50H	No	No
Plain	from11 to 50H	No	No
Plain	from11 to 50H	No	No
Plain	from11 to 50H	No	No
Plain	from11 to 50H	No	No
Plain	from11 to 50H	No	No
Hill	10H	Yes	No
Hill	10H	Yes	No
Hill	10H	Yes	No
Hill	10H	Yes	No
Hill	10H	Yes	No
Hill	10H	Yes	No

Hill	from11 to 50H	Yes	No
Hill	from11 to 50H	Yes	No
Hill	from11 to 50H	Yes	No
Hill	from11 to 50H	Yes	No
Hill	from11 to 50H	Yes	No
Hill	from11 to 50H	Yes	No
Hill	10H	Yes	No
Hill	10H	Yes	No
Hill	10H	Yes	No
Hill	10H	Yes	No
Hill	10H	Yes	No
Hill	10H	Yes	No
Hill	10H	Yes	No
Plain	from11 to 50H	Yes	No
Plain	from11 to 50H	Yes	No
Plain	from11 to 50H	Yes	No
Plain	from11 to 50H	Yes	No
Plain	from11 to 50H	Yes	No
Hill	10H	Yes	No
Hill	10H	Yes	No
Hill	10H	Yes	No
Plain	from11 to 50H	Yes	No
Plain	from11 to 50H	Yes	No
Plain	from11 to 50H	Yes	No
Plain	from11 to 50H	Yes	No
Plain	from11 to 50H	Yes	No
Plain	from11 to 50H	Yes	No
Hill	from11 to 50H	Yes	No
Hill	from11 to 50H	Yes	No
Hill	from11 to 50H	Yes	No
Hill	from11 to 50H	Yes	No
Hill	from11 to 50H	Yes	No
Hill	from11 to 50H	Yes	No
Hill	from11 to 50H	Yes	No
Plain	10H	No	No
Plain	10H	No	No
Plain	10H	No	No
Plain	10H	No	No
Plain	10H	No	No
Plain	10H	No	No
Plain	10H	No	No
Hill	10H	No	No
Hill	10H	No	No
Hill	10H	No	No
Hill	10H	No	No
Plain	from11 to 50H	Yes	No
Plain	from11 to 50H	Yes	No
Plain	from11 to 50H	Yes	No
Plain	from11 to 50H	Yes	No
Hill	10H	No	No

Hill	10H	No	No
Hill	10H	No	No
Hill	10H	No	No
Hill	10H	No	No
Hill	from11 to 50H	No	No
Hill	from11 to 50H	No	No
Hill	from11 to 50H	No	No
Hill	from11 to 50H	No	No
Hill	from11 to 50H	No	No
Hill	from11 to 50H	No	No
Hill	10H	No	No
Hill	10H	No	No
Hill	10H	No	No
Hill	10H	No	No
Hill	10H	No	No
Hill	10H	No	No
Hill	from11 to 50H	No	No
Hill	from11 to 50H	No	No
Hill	from11 to 50H	No	No
Hill	from11 to 50H	No	No
Hill	from11 to 50H	No	No
Hill	from11 to 50H	No	No
Hill	from11 to 50H	No	No
Plain	10H	No	No
Plain	10H	No	No
Plain	10H	No	No
Plain	10H	No	No
Plain	10H	No	No
Plain	10H	No	No
Plain	10H	No	No
Plain	10H	No	No
			No
			No
			No
			No
			No
			No
			No
			No
			No
			No
Hill	from11 to 50H	Yes	No
Hill	from11 to 50H	Yes	No
Hill	from11 to 50H	Yes	No
Hill	from11 to 50H	Yes	No
Hill	from11 to 50H	Yes	No
Hill	from11 to 50H	Yes	No
Hill	from11 to 50H	Yes	No
Mountain	from11 to 50H	Yes	No
Mountain	from11 to 50H	Yes	No

Mountain	from11 to 50H	Yes	No
Mountain	from11 to 50H	Yes	No
Mountain	from11 to 50H	Yes	No
Mountain	from11 to 50H	Yes	No
Mountain	from11 to 50H	Yes	No
Mountain	from11 to 50H	Yes	No
Mountain	from11 to 50H	Yes	No
Mountain	from11 to 50H	Yes	No
Mountain	from11 to 50H	Yes	No
Mountain	from11 to 50H	Yes	No
Mountain	from11 to 50H	Yes	No
Plain	from11 to 50H	Yes	No
Plain	from11 to 50H	Yes	No
Plain	from11 to 50H	Yes	No
Plain	from11 to 50H	Yes	No
Plain	from11 to 50H	Yes	No
Plain	from11 to 50H	Yes	No
Hill	from11 to 50H	Yes	No
Hill	from11 to 50H	Yes	No
Hill	from11 to 50H	Yes	No
Hill	from11 to 50H	Yes	No
Hill	from11 to 50H	Yes	No
Hill	from11 to 50H	Yes	No
Hill	from11 to 50H	Yes	No
Hill	from11 to 50H	Yes	No
Hill	from11 to 50H	Yes	No
Hill	from11 to 50H	Yes	No
Plain	10H	Yes	No
Plain	10H	Yes	No
Plain	10H	Yes	No
Plain	10H	Yes	No
Plain	10H	Yes	No
Plain	10H	Yes	No
Hill	from11 to 50H	Yes	No
Hill	from11 to 50H	Yes	No
Hill	from11 to 50H	Yes	No
Hill	from11 to 50H	Yes	No
Hill	from11 to 50H	Yes	No
Hill	from11 to 50H	Yes	No
Hill	from11 to 50H	Yes	No
Hill	from11 to 50H	Yes	No
Hill	from11 to 50H	Yes	No
Hill	from11 to 50H	Yes	No
Hill	from11 to 50H	Yes	No
Hill	from11 to 50H	Yes	No
Hill	from11 to 50H	Yes	No
Hill	from11 to 50H	Yes	No
Hill	from11 to 50H	Yes	No
Hill	from11 to 50H	Yes	No
Hill	from11 to 50H	Yes	No

Hill	from11 to 50H	Yes	No
Hill	from11 to 50H	Yes	No
Hill	from11 to 50H	Yes	No
Hill	from11 to 50H	Yes	No
Hill	from11 to 50H	Yes	No
Hill	from11 to 50H	Yes	No
Hill	from11 to 50H	Yes	No
Hill	from11 to 50H	Yes	No
Hill	from11 to 50H	Yes	No
Hill	from11 to 50H	Yes	No
Hill	from11 to 50H	Yes	No
Hill	from11 to 50H	Yes	No
Hill	from11 to 50H	Yes	No

Use of concentrate feed	Watering type	Water source	Presence of water source in pasture
Yes	Surface	Tap	No
Yes	Surface	Tap	No
Yes	Surface	Tap	No
Yes	Surface	Tap	No
Yes	Surface	Tap	No
Yes	Surface	Tap	No
Yes	Surface	Tap	No
Yes	Surface	Tap	No
Yes	Surface	Tap	No
Yes	Surface	Tap	No
Yes	Surface	Tap	No
Yes	Surface	Tap	No
Yes	Surface	Tap	Yes
Yes	Surface	Tap	Yes
Yes	Surface	Tap	Yes
Yes	Surface	Tap	Yes
Yes	Surface	Tap	Yes
Yes	Surface	Tap	Yes
Yes	Surface	Tap	Yes
Yes	Surface	Tap	Yes
Yes	Surface	Tap	Yes
Yes	Surface	Tap	Yes
Yes	Surface	Tap	Yes
Yes	Surface	Tap	Yes
Yes	Surface	Tap	Yes
Yes	Surface	Tap	No
Yes	Surface	Tap	No
Yes	Surface	Tap	No
Yes	Surface	Tap	No
Yes	Surface	Tap	No
Yes	Surface	Tap	No
Yes	Surface	Tap	No
Yes	Surface	Tap	
Yes	Surface	Tap	
Yes	Surface	Tap	
Yes	Surface	Tap	
Yes	Mixed	Tap+Well	Yes
Yes	Mixed	Tap+Well	Yes
Yes	Mixed	Tap+Well	Yes
Yes	Mixed	Tap+Well	Yes
Yes	Mixed	Tap+Well	Yes
Yes	Mixed	Tap+Well	Yes
Yes	Mixed	Tap+Well	Yes
Yes	Mixed	Tap+Well	Yes
Yes	Mixed	Tap+Well	Yes
No	Surface	Tap	No
No	Surface	Tap	No

No	Surface	Tap	No
No	Surface	Tap	No
No	Surface	Tap	No
No	Surface	Tap	No
No	Surface	Tap	No
No	Surface	Tap	No
No	Surface	Tap	No
No	Surface	Tap	No
Yes	Mixed	Tap+Well	No
Yes	Mixed	Tap+Well	No
Yes	Mixed	Tap+Well	No
Yes	Mixed	Tap+Well	No
Yes	Mixed	Tap+Well	No
Yes	Mixed	Tap+Well	No
Yes	Mixed	Tap+Well	No
Yes	Mixed	Tap+Well	No
Yes	Mixed	Tap+Well	No
Yes	Mixed	Tap+Well	No
Yes	Mixed	Tap+Well	No
No	Surface	Tap	No
No	Surface	Tap	No
No	Surface	Tap	No
No	Surface	Tap	No
No	Surface	Tap	No
Yes	Mixed	Tap+Well	No
Yes	Mixed	Tap+Well	No
Yes	Mixed	Tap+Well	No
Yes	Mixed	Tap+Well	No
Yes	Mixed	Tap+Well	No
Yes	Mixed	Tap+Well	No
Yes	Mixed	Tap+Well	No
Yes	Surface	Tap	No
Yes	Surface	Tap	No
Yes	Surface	Tap	No
Yes	Surface	Tap	No
Yes	Surface	Tap	No
Yes	Surface	Tap	No
Yes	Surface	Tap	No
Yes	Surface	Tap	No
No	Mixed	Tap+Well	Yes
No	Mixed	Tap+Well	Yes
No	Mixed	Tap+Well	Yes
No	Mixed	Tap+Well	Yes
No	Mixed	Tap+Well	Yes
No	Mixed	Tap+Well	Yes
No	Mixed	Tap+Well	Yes
No	Mixed	Tap+Well	Yes
Yes	Surface	Tap+Valley	Yes
Yes	Surface	Tap+Valley	Yes
Yes	Surface	Tap+Valley	Yes

No	Mixed	Tap+Valley	Yes
No	Mixed	Tap+Valley	Yes
No	Mixed	Tap+Valley	Yes
No	Mixed	Tap+Valley	Yes
No	Mixed	Tap+Valley	Yes
No	Mixed	Tap+Valley	Yes
No	Mixed	Tap+Valley	Yes
No	Mixed	Tap+Valley	Yes
Yes	Surface	Tap	No
Yes	Surface	Tap	No
Yes	Surface	Tap	No
Yes	Surface	Tap	No
Yes	Surface	Tap	No
Yes	Surface	Tap	Yes
Yes	Surface	Tap	Yes
Yes	Surface	Tap	Yes
Yes	Surface	Tap	Yes
Yes	Surface	Tap	Yes
No	Surface	Tap+Valley	No
No	Surface	Tap+Valley	No
No	Surface	Tap+Valley	No
No	Surface	Tap+Valley	No
No	Surface	Tap+Valley	No
Yes	Surface	Tap	
Yes	Surface	Tap	
Yes	Surface	Tap	
Yes	Surface	Tap	
Yes	Surface	Tap	
Yes	Surface	Tap	
No	Mixed	Tap+Valley	No
No	Mixed	Tap+Valley	No
No	Mixed	Tap+Valley	No
No	Mixed	Tap+Valley	No
No	Mixed	Tap+Valley	No
Yes	Mixed	Tap+Valley	No
Yes	Mixed	Tap+Valley	No
Yes	Mixed	Tap+Valley	No
Yes	Mixed	Tap+Valley	No
Yes	Mixed	Tap+Valley	No
Yes	Mixed	Tap+Valley	No
Yes	Surface	Tap	Yes
Yes	Surface	Tap	Yes
Yes	Surface	Tap	Yes
Yes	Surface	Tap	Yes
Yes	Surface	Tap	Yes
Yes	Surface	Tap	No
Yes	Surface	Tap	No
Yes	Surface	Tap	No
Yes	Surface	Tap	No
Yes	Surface	Tap	No

Yes	Surface	Tap	No
Yes	Surface	Tap	No
Yes	Mixed	Tap+Well	No
Yes	Mixed	Tap+Well	No
Yes	Mixed	Tap+Well	No
Yes	Mixed	Tap+Well	No
No	Surface	Tap	No
No	Surface	Tap	No
No	Surface	Tap	No
No	Surface	Tap	No
No	Surface	Tap	No
No	Surface	Tap	No
No	Surface	Tap	No
No	Surface	Tap	No
Yes	Mixed	Tap+Well	No
Yes	Mixed	Tap+Well	No
Yes	Mixed	Tap+Well	No
Yes	Mixed	Tap+Well	No
Yes	Mixed	Tap+Well	No
Yes	Mixed	Tap+Well	No
Yes	Mixed	Tap+Well	No
Yes	Mixed	Tap+Well	No
Yes	Mixed	Tap+Well	No
No	Surface	Tap	No
No	Surface	Tap	No
No	Surface	Tap	No
No	Surface	Tap	No
No	Surface	Tap	No
No	Surface	Tap	No
Yes	Mixed	Tap+Well	Yes
Yes	Mixed	Tap+Well	Yes
Yes	Mixed	Tap+Well	Yes
Yes	Mixed	Tap+Well	Yes
Yes	Mixed	Tap+Well	Yes
Yes	Mixed	Tap+Well	No
Yes	Mixed	Tap+Well	No
Yes	Mixed	Tap+Well	No
Yes	Mixed	Tap+Well	No
Yes	Mixed	Tap+Well	No
Yes	Mixed	Tap+Well	No
Yes	Surface	Tap	No
Yes	Surface	Tap	No
Yes	Surface	Tap	No
Yes	Surface	Tap	No
Yes	Surface	Tap	No
No	Surface	Tap	No
No	Surface	Tap	No
No	Surface	Tap	No
No	Surface	Tap	No
Yes	Mixed	Tap+Valley	Yes

Yes	Mixed	Tap+Valley	Yes
Yes	Mixed	Tap+Valley	Yes
Yes	Mixed	Tap+Valley	Yes
Yes	Mixed	Tap+Valley	Yes
Yes	Mixed	Tap+Valley	Yes
Yes	Mixed	Tap+Valley	Yes
Yes	Mixed	Tap+Valley	Yes
Yes	Mixed	Tap+Valley	Yes
No	Surface	Tap	Yes
No	Surface	Tap	Yes
No	Surface	Tap	Yes
No	Surface	Tap	Yes
No	Surface	Tap	Yes
No	Surface	Tap	No
No	Surface	Tap	No
No	Mixed	Tap+Valley	Yes
No	Mixed	Tap+Valley	Yes
No	Mixed	Tap+Valley	Yes
No	Mixed	Tap+Valley	Yes
No	Mixed	Tap+Valley	Yes
No	Mixed	Tap+Valley	Yes
Yes	Surface	Tap	Yes
Yes	Surface	Tap	Yes
Yes	Surface	Tap	Yes
Yes	Surface	Tap	Yes
Yes	Surface	Tap	Yes
Yes	Surface	Tap	Yes
Yes	Surface	Tap	Yes
No	Mixed	Tap+Well	Yes
No	Mixed	Tap+Well	Yes
No	Mixed	Tap+Well	Yes
No	Mixed	Tap+Well	Yes
No	Mixed	Tap+Well	Yes
No	Mixed	Tap+Well	Yes
No	Surface	Tap	No
No	Surface	Tap	No
No	Surface	Tap	No
No	Surface	Tap	No
No	Surface	Tap	No
No	Surface	Tap	No
Yes	Surface	Tap+Well	No
Yes	Surface	Tap+Well	No
Yes	Surface	Tap+Well	No
Yes	Surface	Tap+Well	No
Yes	Surface	Tap+Valley	No
Yes	Surface	Tap+Valley	No
Yes	Surface	Tap+Valley	No
Yes	Surface	Tap+Valley	No
Yes	Surface	Tap+Valley	No
Yes	Surface	Tap	

Yes	Surface	Tap	
Yes	Surface	Tap	
Yes	Surface	Tap	
Yes	Surface	Tap	
Yes	Surface	Tap	
Yes	Surface	Tap	
No	Mixed	Tap+Well	No
No	Mixed	Tap+Well	No
No	Mixed	Tap+Well	No
No	Mixed	Tap+Well	No
No	Mixed	Tap+Well	No
No	Mixed	Tap+Well	No
No	Mixed	Tap+Well	No
Yes	Surface	Tap	No
Yes	Surface	Tap	No
Yes	Surface	Tap	No
Yes	Surface	Tap	No
Yes	Surface	Tap	No
Yes	Surface	Tap	No
Yes	Surface	Tap	No
Yes	Surface	Tap	No
Yes	Surface	Tap	No
Yes	Surface	Tap	No
Yes	Surface	Tap	No
Yes	Surface	Tap	No
Yes	Surface	Tap	No
Yes	Surface	Tap	No
Yes	Surface	Tap	No
Yes	Surface	Tap	No
No	Mixed	Tap+Well	Yes
No	Mixed	Tap+Well	Yes
No	Mixed	Tap+Well	Yes
No	Mixed	Tap+Well	Yes
No	Mixed	Tap+Well	Yes
No	Mixed	Tap+Well	Yes
Yes	Mixed	Tap+Well	Yes
Yes	Mixed	Tap+Well	Yes
Yes	Mixed	Tap+Well	Yes
Yes	Mixed	Tap+Well	Yes
Yes	Mixed	Tap+Well	Yes
Yes	Mixed	Tap+Well	Yes
Yes	Mixed	Tap+Well	Yes
Yes	Mixed	Tap+Well	Yes
Yes	Mixed	Tap+Well	Yes
No	Surface	Tap	No
No	Surface	Tap	No
No	Surface	Tap	No
No	Surface	Tap	No
No	Surface	Tap	No
No	Surface	Tap	No

Yes	Surface	Tap+Valley	Yes
Yes	Surface	Tap+Valley	Yes
Yes	Surface	Tap+Valley	Yes
Yes	Surface	Tap+Valley	Yes
Yes	Surface	Tap+Valley	Yes
Yes	Surface	Tap+Valley	Yes
Yes	Surface	Tap+Valley	Yes
Yes	Surface	Tap+Valley	Yes
Yes	Surface	Tap+Valley	Yes
Yes	Surface	Tap+Valley	Yes
Yes	Surface	Tap+Valley	Yes
Yes	Surface	Tap+Valley	Yes
Yes	Surface	Tap+Valley	Yes
No	Surface	Tap	No
No	Surface	Tap	No
No	Surface	Tap	No
No	Surface	Tap	No
No	Surface	Tap	No
No	Surface	Tap+Valley	No
No	Surface	Tap+Valley	No
No	Surface	Tap+Valley	No
Yes	Mixed	Tap+Well	Yes
Yes	Mixed	Tap+Well	Yes
Yes	Mixed	Tap+Well	Yes
Yes	Mixed	Tap+Well	Yes
Yes	Mixed	Tap+Well	Yes
Yes	Mixed	Tap+Well	Yes
Yes	Surface	Tap	No
Yes	Surface	Tap	No
Yes	Surface	Tap	No
Yes	Surface	Tap	No
Yes	Surface	Tap	No
Yes	Surface	Tap	No
Yes	Surface	Tap	No
No	Mixed	Tap+Well	No
No	Mixed	Tap+Well	No
No	Mixed	Tap+Well	No
No	Mixed	Tap+Well	No
No	Mixed	Tap+Well	No
No	Mixed	Tap+Well	No
No	Mixed	Tap+Well	No
Yes	Surface	Tap+Valley	No
Yes	Surface	Tap+Valley	No
Yes	Surface	Tap+Valley	No
Yes	Surface	Tap+Valley	No
Yes	Surface	Tap	No
Yes	Surface	Tap	No
Yes	Surface	Tap	No
Yes	Surface	Tap	No
Yes	Mixed	Tap+Well	No

Yes	Mixed	Tap+Well	No
Yes	Mixed	Tap+Well	No
Yes	Mixed	Tap+Well	No
Yes	Mixed	Tap+Well	No
No	Surface	Tap	No
No	Surface	Tap	No
No	Surface	Tap	No
No	Surface	Tap	No
No	Surface	Tap	No
No	Surface	Tap	No
Yes	Surface	Tap	No
Yes	Surface	Tap	No
Yes	Surface	Tap	No
Yes	Surface	Tap	No
Yes	Surface	Tap	No
Yes	Surface	Tap	No
Yes	Surface	Tap	No
Yes	Surface	Tap	No
Yes	Surface	Tap	No
Yes	Surface	Tap	No
Yes	Surface	Tap	No
Yes	Surface	Tap	No
Yes	Surface	Tap	No
Yes	Mixed	Tap+Well	Yes
Yes	Mixed	Tap+Well	Yes
Yes	Mixed	Tap+Well	Yes
Yes	Mixed	Tap+Well	Yes
Yes	Mixed	Tap+Well	Yes
Yes	Mixed	Tap+Well	Yes
Yes	Mixed	Tap+Well	Yes
Yes	Mixed	Tap+Well	Yes
Yes	Surface	Tap	
Yes	Surface	Tap	
Yes	Surface	Tap	
Yes	Surface	Tap	
Yes	Surface	Tap	
Yes	Surface	Tap	
Yes	Surface	Tap	
Yes	Surface	Tap	
Yes	Surface	Tap	
Yes	Surface	Tap	
No	Surface	Tap	No
No	Surface	Tap	No
No	Surface	Tap	No
No	Surface	Tap	No
No	Surface	Tap	No
No	Surface	Tap	No
No	Surface	Tap	No
Yes	Mixed	Tap+Well	No
Yes	Mixed	Tap+Well	No

Yes	Mixed	Tap+Well	No
Yes	Mixed	Tap+Well	No
Yes	Mixed	Tap+Well	No
Yes	Mixed	Tap+Well	No
Yes	Mixed	Tap+Well	No
Yes	Mixed	Tap+Well	No
Yes	Mixed	Tap+Well	No
Yes	Mixed	Tap+Well	No
Yes	Mixed	Tap+Well	No
Yes	Mixed	Tap+Well	No
Yes	Mixed	Tap+Well	No
Yes	Mixed	Tap	No
Yes	Mixed	Tap	No
Yes	Mixed	Tap	No
No	Mixed	Tap	No
No	Mixed	Tap	No
No	Mixed	Tap	No
Yes	Surface	Tap	No
Yes	Surface	Tap	No
Yes	Surface	Tap	No
Yes	Surface	Tap	No
Yes	Surface	Tap	No
Yes	Surface	Tap	No
Yes	Surface	Tap	No
Yes	Surface	Tap	No
Yes	Surface	Tap	No
Yes	Surface	Tap	No
Yes	Surface	Tap	No
Yes	Surface	Tap	No
Yes	Surface	Tap	No
Yes	Surface	Tap	No
Yes	Surface	Tap	No
Yes	Surface	Tap	No
No	Surface	Tap+Well	No
No	Surface	Tap+Well	No
No	Surface	Tap+Well	No
No	Surface	Tap+Well	No
No	Surface	Tap+Well	No
No	Surface	Tap+Well	No
No	Surface	Tap+Well	No
No	Surface	Tap+Well	No
No	Surface	Tap+Well	No
No	Surface	Tap+Well	No
Yes	Surface	Tap	No
Yes	Surface	Tap	No
Yes	Surface	Tap	No
Yes	Surface	Tap	No
Yes	Surface	Tap	No
Yes	Surface	Tap	No
Yes	Surface	Tap	No

Yes	Surface	Tap	No
Yes	Surface	Tap	No
Yes	Surface	Tap	No
Yes	Surface	Tap	No
Yes	Surface	Tap	No
Yes	Surface	Tap	No
Yes	Surface	Tap	No
Yes	Surface	Tap	No
Yes	Surface	Tap	No
Yes	Surface	Tap	No
Yes	Surface	Tap	No
Yes	Surface	Tap	No
Yes	Surface	Tap	No

Location of the water trough	Species on the farm	Presence of other farm animals
Interior	goat	Yes
Interior	goat	Yes
Interior	goat	Yes
Interior	goat	Yes
Interior	goat	Yes
Interior	goat	Yes
Interior	goat	Yes
Interior	goat	Yes
Interior	goat	Yes
Interior	goat	Yes
Interior	goat	Yes
Interior	goat	Yes
Interior	goat+sheep	Yes
Interior	goat+sheep	Yes
Interior	goat+sheep	Yes
Interior	goat+sheep	Yes
Interior	goat+sheep	Yes
Interior	goat	Yes
Interior	goat	Yes
Interior	goat	Yes
Interior	goat	Yes
Interior	goat	Yes
Interior	goat	Yes
Interior	goat	Yes
Interior	goat	Yes
Exterior	goat+sheep	Yes
Exterior	goat+sheep	Yes
Exterior	goat+sheep	Yes
Exterior	goat+sheep	Yes
Exterior	goat+sheep	Yes
Exterior	goat+sheep	Yes
Exterior	goat+sheep	Yes
Interior	goat+sheep	Yes
Interior	goat+sheep	Yes
Interior	goat+sheep	Yes
Interior	goat+sheep	Yes
Interior	goat	Yes
Interior	goat	Yes
Interior	goat	Yes
Interior	goat	Yes
Interior	goat	Yes
Interior	goat	Yes
Interior	goat	Yes
Interior	goat	Yes
Interior	goat	Yes
Interior	goat+sheep	Yes
Interior	goat+sheep	Yes

Interior	goat+sheep	Yes
Interior	goat+sheep	Yes
Interior	goat+sheep	Yes
Interior	goat+sheep	Yes
Interior	goat+sheep	Yes
Interior	goat+sheep	Yes
Interior	goat+sheep	Yes
Interior	goat+sheep	Yes
Interior+Exterior	goat+sheep	Yes
Interior+Exterior	goat+sheep	Yes
Interior+Exterior	goat+sheep	Yes
Interior+Exterior	goat+sheep	Yes
Interior+Exterior	goat+sheep	Yes
Interior+Exterior	goat+sheep	Yes
Interior+Exterior	goat+sheep	Yes
Interior+Exterior	goat+sheep	Yes
Interior+Exterior	goat+sheep	Yes
Interior+Exterior	goat+sheep	Yes
Interior+Exterior	goat+sheep	Yes
Interior+Exterior	goat	Yes
Interior+Exterior	goat	Yes
Interior+Exterior	goat	Yes
Interior+Exterior	goat	Yes
Interior+Exterior	goat	Yes
Interior+Exterior	goat	Yes
Interior+Exterior	goat	Yes
Interior+Exterior	goat	Yes
Interior+Exterior	goat	Yes
Interior+Exterior	goat	Yes
Interior+Exterior	goat	Yes
Interior+Exterior	goat	Yes
Interior	goat	Yes
Interior	goat	Yes
Interior	goat	Yes
Interior	goat	Yes
Interior	goat	Yes
Interior	goat	Yes
Interior	goat	Yes
Interior	goat	Yes
Interior+Exterior	goat+sheep	Yes
Interior+Exterior	goat+sheep	Yes
Interior+Exterior	goat+sheep	Yes
Interior+Exterior	goat+sheep	Yes
Interior+Exterior	goat+sheep	Yes
Interior+Exterior	goat+sheep	Yes
Interior+Exterior	goat+sheep	Yes
Interior+Exterior	goat+sheep	Yes
Interior	goat	No
Interior	goat	No
Interior	goat	No

Interior+Exterior	goat+sheep	Yes
Interior+Exterior	goat+sheep	Yes
Interior+Exterior	goat+sheep	Yes
Interior+Exterior	goat+sheep	Yes
Interior+Exterior	goat+sheep	Yes
Interior+Exterior	goat+sheep	Yes
Interior+Exterior	goat+sheep	Yes
Interior+Exterior	goat+sheep	Yes
Interior	goat	Yes
Interior	goat	Yes
Interior	goat	Yes
Interior	goat	Yes
Interior	goat	Yes
Interior	goat+sheep	Yes
Interior	goat+sheep	Yes
Interior	goat+sheep	Yes
Interior	goat+sheep	Yes
Interior	goat+sheep	Yes
Interior	goat	Yes
Interior	goat	Yes
Interior	goat	Yes
Interior	goat	Yes
Interior	goat	Yes
Interior	goat	Yes
Interior	goat	Yes
Interior	goat	Yes
Interior	goat	Yes
Interior	goat	Yes
Interior	goat	Yes
Interior+Exterior	goat+sheep	Yes
Interior+Exterior	goat+sheep	Yes
Interior+Exterior	goat+sheep	Yes
Interior+Exterior	goat+sheep	Yes
Interior+Exterior	goat+sheep	Yes
Interior+Exterior	goat	Yes
Interior+Exterior	goat	Yes
Interior+Exterior	goat	Yes
Interior+Exterior	goat	Yes
Interior+Exterior	goat	Yes
Interior+Exterior	goat	Yes
Interior	goat+sheep	Yes
Interior	goat+sheep	Yes
Interior	goat+sheep	Yes
Interior	goat+sheep	Yes
Interior	goat+sheep	Yes
Interior	goat	Yes
Interior	goat	Yes
Interior	goat	Yes
Interior	goat	Yes
Interior	goat	Yes

Interior	goat	Yes
Interior	goat	Yes
Interior+Exterior	goat+sheep	Yes
Interior+Exterior	goat+sheep	Yes
Interior+Exterior	goat+sheep	Yes
Interior+Exterior	goat+sheep	Yes
Interior+Exterior	goat	Yes
Interior+Exterior	goat	Yes
Interior+Exterior	goat	Yes
Interior+Exterior	goat	Yes
Interior	goat	Yes
Interior	goat	Yes
Interior	goat	Yes
Interior	goat	Yes
Interior+Exterior	goat+sheep	Yes
Interior+Exterior	goat+sheep	Yes
Interior+Exterior	goat+sheep	Yes
Interior+Exterior	goat+sheep	Yes
Interior+Exterior	goat+sheep	Yes
Interior+Exterior	goat+sheep	Yes
Interior+Exterior	goat+sheep	Yes
Interior+Exterior	goat+sheep	Yes
Interior+Exterior	goat+sheep	Yes
Interior+Exterior	goat	Yes
Interior+Exterior	goat	Yes
Interior+Exterior	goat	Yes
Interior+Exterior	goat	Yes
Interior+Exterior	goat	Yes
Interior+Exterior	goat	Yes
Interior+Exterior	goat+sheep	Yes
Interior+Exterior	goat+sheep	Yes
Interior+Exterior	goat+sheep	Yes
Interior+Exterior	goat+sheep	Yes
Interior+Exterior	goat+sheep	Yes
Interior+Exterior	goat+sheep	Yes
Interior+Exterior	goat+sheep	Yes
Interior+Exterior	goat+sheep	Yes
Interior+Exterior	goat+sheep	Yes
Interior+Exterior	goat+sheep	Yes
Interior+Exterior	goat+sheep	Yes
Interior	goat	Yes
Interior	goat	Yes
Interior	goat	Yes
Interior	goat	Yes
Interior	goat	Yes
Interior	goat+sheep	Yes
Interior	goat+sheep	Yes
Interior	goat+sheep	Yes
Interior	goat+sheep	Yes
Interior+Exterior	goat+sheep	Yes

Interior+Exterior	goat+sheep	Yes
Interior+Exterior	goat+sheep	Yes
Interior+Exterior	goat+sheep	Yes
Interior+Exterior	goat+sheep	Yes
Interior+Exterior	goat+sheep	Yes
Interior+Exterior	goat+sheep	Yes
Interior+Exterior	goat+sheep	Yes
Interior+Exterior	goat+sheep	Yes
Interior	goat	Yes
Interior	goat	Yes
Interior	goat	Yes
Interior	goat	Yes
Interior	goat	Yes
Exterior	goat	Yes
Exterior	goat	Yes
Exterior	goat+sheep	Yes
Exterior	goat+sheep	Yes
Exterior	goat+sheep	Yes
Exterior	goat+sheep	Yes
Exterior	goat+sheep	Yes
Exterior	goat+sheep	Yes
Interior+Exterior	goat+sheep	Yes
Interior+Exterior	goat+sheep	Yes
Interior+Exterior	goat+sheep	Yes
Interior+Exterior	goat+sheep	Yes
Interior+Exterior	goat+sheep	Yes
Interior+Exterior	goat+sheep	Yes
Interior+Exterior	goat+sheep	Yes
Interior+Exterior	goat	Yes
Interior+Exterior	goat	Yes
Interior+Exterior	goat	Yes
Interior+Exterior	goat	Yes
Interior+Exterior	goat	Yes
Interior+Exterior	goat	Yes
Interior	goat+sheep	Yes
Interior	goat+sheep	Yes
Interior	goat+sheep	Yes
Interior	goat+sheep	Yes
Interior	goat+sheep	Yes
Interior	goat+sheep	Yes
Interior+Exterior	goat+sheep	Yes
Interior+Exterior	goat+sheep	Yes
Interior+Exterior	goat+sheep	Yes
Interior+Exterior	goat+sheep	Yes
Interior+Exterior	goat	Yes
Interior+Exterior	goat	Yes
Interior+Exterior	goat	Yes
Interior+Exterior	goat	Yes
Interior+Exterior	goat	Yes
Interior	goat	Yes

Interior	goat	Yes
Interior	goat	Yes
Interior	goat	Yes
Interior	goat	Yes
Interior	goat	Yes
Interior	goat	Yes
Interior+Exterior	goat+sheep	Yes
Interior+Exterior	goat+sheep	Yes
Interior+Exterior	goat+sheep	Yes
Interior+Exterior	goat+sheep	Yes
Interior+Exterior	goat+sheep	Yes
Interior+Exterior	goat+sheep	Yes
Interior+Exterior	goat+sheep	Yes
Interior	goat	Yes
Interior	goat	Yes
Interior	goat	Yes
Interior	goat	Yes
Interior	goat	Yes
Interior	goat	Yes
Interior	goat	Yes
Interior	goat	Yes
Interior	goat	Yes
Interior+Exterior	goat+sheep	Yes
Interior+Exterior	goat+sheep	Yes
Interior+Exterior	goat+sheep	Yes
Interior+Exterior	goat+sheep	Yes
Interior+Exterior	goat+sheep	Yes
Interior+Exterior	goat+sheep	Yes
Interior+Exterior	goat+sheep	Yes
Interior+Exterior	goat+sheep	Yes
Interior+Exterior	goat+sheep	Yes
Interior+Exterior	goat+sheep	Yes
Interior+Exterior	goat+sheep	No
Interior+Exterior	goat+sheep	No
Interior+Exterior	goat+sheep	No
Interior+Exterior	goat+sheep	Yes
Interior+Exterior	goat+sheep	Yes
Interior+Exterior	goat+sheep	Yes
Interior+Exterior	goat+sheep	Yes
Interior+Exterior	goat+sheep	Yes
Interior+Exterior	goat+sheep	Yes
Interior+Exterior	goat+sheep	Yes
Interior+Exterior	goat+sheep	Yes
Interior+Exterior	goat+sheep	Yes
Interior	goat+sheep	Yes
Interior	goat+sheep	Yes
Interior	goat+sheep	Yes
Interior	goat+sheep	Yes
Interior	goat+sheep	Yes
Interior	goat+sheep	Yes

Interior	goat	Yes
Interior	goat	Yes
Interior	goat	Yes
Interior	goat	Yes
Interior	goat	Yes
Interior	goat	Yes
Interior	goat+sheep	Yes
Interior	goat+sheep	Yes
Interior	goat+sheep	Yes
Interior	goat+sheep	Yes
Interior	goat+sheep	Yes
Interior	goat+sheep	Yes
Interior	goat+sheep	Yes
Interior	goat+sheep	Yes
Interior	goat+sheep	Yes
Interior	goat+sheep	Yes
Interior	goat+sheep	Yes
Interior	goat+sheep	Yes
Interior+Exterior	goat+sheep	Yes
Interior+Exterior	goat+sheep	Yes
Interior+Exterior	goat+sheep	Yes
Interior+Exterior	goat+sheep	Yes
Interior+Exterior	goat+sheep	Yes
Interior+Exterior	goat+sheep	Yes
Interior+Exterior	goat+sheep	Yes
Interior+Exterior	goat+sheep	Yes
Interior+Exterior	goat+sheep	Yes
Interior	goat+sheep	Yes
Interior	goat+sheep	Yes
Interior	goat+sheep	Yes
Interior	goat+sheep	Yes
Interior	goat+sheep	Yes
Interior	goat+sheep	Yes
Interior	goat+sheep	Yes
Interior	goat	Yes
Interior	goat	Yes
Interior	goat	Yes
Interior	goat	Yes
Interior	goat	Yes
Interior	goat	Yes
Interior	goat	Yes
Interior+Exterior	goat+sheep	Yes
Interior+Exterior	goat+sheep	Yes
Interior+Exterior	goat+sheep	Yes
Interior+Exterior	goat+sheep	Yes
Interior+Exterior	goat	Yes
Interior+Exterior	goat	Yes
Interior+Exterior	goat	Yes
Interior+Exterior	goat	Yes
Interior	goat+sheep	Yes

Interior	goat+sheep	Yes
Interior	goat+sheep	Yes
Interior	goat+sheep	Yes
Interior	goat+sheep	Yes
Interior+Exterior	goat+sheep	Yes
Interior+Exterior	goat+sheep	Yes
Interior+Exterior	goat+sheep	Yes
Interior+Exterior	goat+sheep	Yes
Interior+Exterior	goat+sheep	Yes
Interior+Exterior	goat+sheep	Yes
Interior	goat	Yes
Interior	goat	Yes
Interior	goat	Yes
Interior	goat	Yes
Interior	goat	Yes
Interior	goat	Yes
Interior	goat	Yes
Interior	goat	Yes
Interior	goat	Yes
Interior	goat	Yes
Interior	goat	Yes
Interior	goat	Yes
Interior	goat	Yes
Interior+Exterior	goat+sheep	Yes
Interior+Exterior	goat+sheep	Yes
Interior+Exterior	goat+sheep	Yes
Interior+Exterior	goat+sheep	Yes
Interior+Exterior	goat+sheep	Yes
Interior+Exterior	goat+sheep	Yes
Interior+Exterior	goat+sheep	Yes
Interior+Exterior	goat+sheep	Yes
Interior	goat+sheep	Yes
Interior	goat+sheep	Yes
Interior	goat+sheep	Yes
Interior	goat+sheep	Yes
Interior	goat+sheep	Yes
Exterior	goat+sheep	Yes
Exterior	goat+sheep	Yes
Exterior	goat+sheep	Yes
Exterior	goat+sheep	Yes
Exterior	goat+sheep	Yes
Interior+Exterior	goat+sheep	Yes
Interior+Exterior	goat+sheep	Yes
Interior+Exterior	goat+sheep	Yes
Interior+Exterior	goat+sheep	Yes
Interior+Exterior	goat+sheep	Yes
Interior+Exterior	goat+sheep	Yes
Interior+Exterior	goat+sheep	Yes
Interior	goat+sheep	Yes
Interior	goat+sheep	Yes

Interior	goat+sheep	Yes
Interior	goat+sheep	Yes
Interior	goat+sheep	Yes
Interior	goat+sheep	Yes
Interior	goat+sheep	Yes
Interior	goat+sheep	Yes
Interior	goat+sheep	Yes
Interior	goat+sheep	Yes
Interior	goat+sheep	Yes
Interior	goat+sheep	Yes
Interior	goat+sheep	Yes
Interior	goat+sheep	Yes
Interior	goat+sheep	Yes
Interior	goat+sheep	Yes
Interior	goat+sheep	Yes
Interior	goat+sheep	Yes
Interior	goat+sheep	Yes
Exterior	goat+sheep	Yes
Exterior	goat+sheep	Yes
Exterior	goat+sheep	Yes
Exterior	goat+sheep	Yes
Exterior	goat+sheep	Yes
Interior	goat+sheep	Yes
Interior	goat+sheep	Yes
Interior	goat+sheep	Yes
Interior	goat+sheep	Yes
Interior	goat+sheep	Yes
Interior	goat+sheep	Yes
Interior	goat+sheep	Yes
Interior	goat+sheep	Yes
Interior	goat+sheep	Yes
Interior	goat+sheep	Yes
Interior	goat+sheep	Yes
Interior+Exterior	goat+sheep	Yes
Interior+Exterior	goat+sheep	Yes
Interior+Exterior	goat+sheep	Yes
Interior+Exterior	goat+sheep	Yes
Interior+Exterior	goat+sheep	Yes
Interior+Exterior	goat+sheep	Yes
Interior	goat	Yes
Interior	goat	Yes
Interior	goat	Yes
Interior	goat	Yes
Interior+Exterior	goat+sheep	Yes
Interior+Exterior	goat+sheep	Yes
Interior+Exterior	goat+sheep	Yes
Interior+Exterior	goat+sheep	Yes
Interior+Exterior	goat+sheep	Yes
Interior+Exterior	goat+sheep	Yes
Interior	goat	Yes

Interior	goat	Yes
Interior	goat	Yes
Interior	goat	Yes
Interior	goat	Yes
Interior	goat	Yes
Interior	goat	Yes
Interior	goat	Yes
Interior	goat	Yes
Interior	goat	Yes
Interior	goat	Yes
Interior	goat	Yes
Interior	goat	Yes
Interior	goat	Yes

Presence of cattle	Presence of equids	Presence of poultry	Presence of rodents
No	No	No	Yes
No	No	No	Yes
No	No	No	Yes
No	No	No	Yes
No	No	No	Yes
No	No	No	Yes
No	No	No	Yes
No	No	No	Yes
No	No	No	Yes
No	No	No	Yes
No	No	No	Yes
No	No	No	Yes
No	No	No	No
No	No	No	No
No	No	No	No
No	No	No	No
No	No	No	No
No	No	Yes	No
No	No	Yes	No
No	No	Yes	No
No	No	Yes	No
No	No	Yes	No
No	No	Yes	No
No	No	Yes	No
No	No	Yes	No
No	No	Yes	No
No	No	Yes	No
No	No	Yes	No
No	No	Yes	No
No	No	Yes	No
No	No	Yes	No
No	No	Yes	No
No	No	No	No
No	No	No	No
No	No	No	No
No	No	No	No
Yes	No	No	Yes
Yes	No	No	Yes
Yes	No	No	Yes
Yes	No	No	Yes
Yes	No	No	Yes
Yes	No	No	Yes
Yes	No	No	Yes
Yes	No	No	Yes
Yes	No	No	Yes
No	No	No	Yes
No	No	No	Yes

No	No	No	Yes
No	No	No	Yes
No	No	No	Yes
No	No	No	Yes
No	No	No	Yes
No	No	No	Yes
No	No	No	Yes
No	No	No	Yes
Yes	Yes	Yes	Yes
Yes	Yes	Yes	Yes
Yes	Yes	Yes	Yes
Yes	Yes	Yes	Yes
Yes	Yes	Yes	Yes
Yes	Yes	Yes	Yes
Yes	Yes	Yes	Yes
Yes	Yes	Yes	Yes
Yes	Yes	Yes	Yes
Yes	Yes	Yes	Yes
Yes	Yes	Yes	Yes
No	No	Yes	No
No	No	Yes	No
No	No	Yes	No
No	No	Yes	No
No	No	Yes	No
No	No	Yes	Yes
No	No	Yes	Yes
No	No	Yes	Yes
No	No	Yes	Yes
No	No	Yes	Yes
No	No	Yes	Yes
No	No	Yes	Yes
Yes	No	Yes	No
Yes	No	Yes	No
Yes	No	Yes	No
Yes	No	Yes	No
Yes	No	Yes	No
Yes	No	Yes	No
Yes	No	Yes	No
Yes	No	Yes	No
Yes	No	Yes	Yes
Yes	No	Yes	Yes
Yes	No	Yes	Yes
Yes	No	Yes	Yes
Yes	No	Yes	Yes
Yes	No	Yes	Yes
Yes	No	Yes	Yes
Yes	No	Yes	Yes
No	No	No	No
No	No	No	No
No	No	No	No

Yes	No	Yes	No
Yes	No	Yes	No
Yes	No	Yes	No
Yes	No	Yes	No
Yes	No	Yes	No
Yes	No	Yes	No
Yes	No	Yes	No
Yes	No	Yes	No
No	No	No	Yes
No	No	No	Yes
No	No	No	Yes
No	No	No	Yes
No	No	No	Yes
No	No	Yes	No
No	No	Yes	No
No	No	Yes	No
No	No	Yes	No
No	No	Yes	No
Yes	No	No	No
Yes	No	No	No
Yes	No	No	No
Yes	No	No	No
Yes	No	No	No
No	No	No	Yes
No	No	No	Yes
No	No	No	Yes
No	No	No	Yes
No	No	No	Yes
No	No	No	Yes
Yes	No	Yes	No
Yes	No	Yes	No
Yes	No	Yes	No
Yes	No	Yes	No
Yes	No	Yes	No
No	No	No	No
No	No	No	No
No	No	No	No
No	No	No	No
No	No	No	No
No	No	No	No
No	No	No	No
No	No	No	No
No	No	No	No
No	No	No	No
No	No	No	No
Yes	No	No	Yes
Yes	No	No	Yes
Yes	No	No	Yes
Yes	No	No	Yes
Yes	No	No	Yes

Yes	No	No	Yes
Yes	No	No	Yes
No	No	Yes	No
No	No	Yes	No
No	No	Yes	No
No	No	Yes	No
Yes	No	No	No
Yes	No	No	No
Yes	No	No	No
Yes	No	No	No
No	No	No	No
No	No	No	No
No	No	No	No
No	No	No	No
No	No	No	No
No	No	No	No
No	No	No	No
No	No	No	No
No	No	No	No
No	No	No	No
No	No	No	No
No	No	No	No
No	No	No	No
No	No	Yes	No
No	No	Yes	No
No	No	Yes	No
No	No	Yes	No
No	No	Yes	No
No	No	Yes	No
Yes	No	Yes	No
Yes	No	Yes	No
Yes	No	Yes	No
Yes	No	Yes	No
Yes	No	Yes	No
No	No	Yes	Yes
No	No	Yes	Yes
No	No	Yes	Yes
No	No	Yes	Yes
No	No	Yes	Yes
No	No	Yes	Yes
No	No	No	Yes
No	No	No	Yes
No	No	No	Yes
No	No	No	Yes
No	No	No	Yes
Yes	No	Yes	No
Yes	No	Yes	No
Yes	No	Yes	No
Yes	No	Yes	No
No	Yes	Yes	No

No	Yes	Yes	No
No	Yes	Yes	No
No	Yes	Yes	No
No	Yes	Yes	No
No	Yes	Yes	No
No	Yes	Yes	No
No	Yes	Yes	No
No	Yes	Yes	No
No	No	No	No
No	No	No	No
No	No	No	No
No	No	No	No
No	No	No	No
No	No	No	No
No	No	No	No
Yes	Yes	Yes	No
Yes	Yes	Yes	No
Yes	Yes	Yes	No
Yes	Yes	Yes	No
Yes	Yes	Yes	No
Yes	Yes	No	No
No	No	No	Yes
No	No	No	Yes
No	No	No	Yes
No	No	No	Yes
No	No	No	Yes
No	No	No	Yes
No	No	No	Yes
Yes	No	No	Yes
Yes	No	No	Yes
Yes	No	No	Yes
Yes	No	No	Yes
Yes	No	No	Yes
Yes	No	No	Yes
No	No	Yes	No
No	No	Yes	No
No	No	Yes	No
No	No	Yes	No
No	No	Yes	No
No	No	Yes	No
No	No	Yes	No
No	No	Yes	No
No	No	Yes	No
No	No	Yes	No
No	Yes	Yes	No
No	Yes	Yes	No
No	Yes	Yes	No
No	Yes	Yes	No
No	Yes	Yes	No
Yes	No	No	Yes

Yes	No	No	Yes
Yes	No	No	Yes
Yes	No	No	Yes
Yes	No	No	Yes
Yes	No	No	Yes
Yes	No	No	Yes
Yes	Yes	No	No
Yes	Yes	No	No
Yes	Yes	No	No
Yes	Yes	No	No
Yes	Yes	No	No
Yes	Yes	No	No
Yes	Yes	No	No
No	No	No	No
No	No	No	No
No	No	No	No
No	No	No	No
No	No	No	No
No	No	No	No
No	No	No	No
No	No	No	No
No	No	No	No
Yes	No	Yes	No
Yes	No	Yes	No
Yes	No	Yes	No
Yes	No	Yes	No
Yes	No	Yes	No
Yes	No	Yes	No
Yes	No	Yes	No
No	No	Yes	No
No	No	Yes	No
No	No	Yes	No
No	No	Yes	No
No	No	Yes	No
No	No	Yes	No
Yes	No	No	No
Yes	No	No	No
Yes	No	No	No
Yes	No	No	No
Yes	No	No	No
Yes	No	No	No
Yes	No	No	No
Yes	No	No	No
Yes	No	No	No
No	No	No	Yes
No	No	No	Yes
No	No	No	Yes
No	No	No	Yes
No	No	No	Yes
No	No	No	Yes

No	No	Yes	No
No	No	Yes	No
No	No	Yes	No
No	No	Yes	No
No	No	Yes	No
No	No	Yes	No
Yes	No	No	No
Yes	No	No	No
Yes	No	No	No
Yes	No	No	No
Yes	No	No	No
Yes	No	No	No
Yes	No	No	No
No	No	No	No
No	No	No	No
No	No	No	No
No	No	No	No
No	No	No	No
No	No	No	No
No	No	No	No
No	No	No	No
Yes	No	Yes	No
Yes	No	Yes	No
Yes	No	Yes	No
Yes	No	Yes	No
Yes	No	Yes	No
Yes	No	Yes	No
No	No	Yes	No
No	No	Yes	No
No	No	Yes	No
No	No	Yes	No
No	No	Yes	No
No	No	Yes	No
No	No	Yes	No
No	No	Yes	No
No	No	Yes	No
No	No	Yes	No
No	No	Yes	No
No	No	Yes	No
No	No	Yes	No
No	No	Yes	No
No	No	No	No
No	No	No	No
No	No	No	No
No	No	No	No
No	No	No	No
No	No	No	No
No	No	No	No
No	No	No	No
No	No	Yes	No

No	No	Yes	No
No	No	Yes	No
No	No	Yes	No
No	No	Yes	No
No	No	Yes	No
No	No	Yes	No
No	No	Yes	No
No	No	Yes	No
No	No	Yes	No
No	No	Yes	No
No	No	No	Yes
No	No	No	Yes
No	No	No	Yes
No	No	No	Yes
No	No	No	Yes
No	No	No	Yes
No	No	No	Yes
No	No	No	Yes
No	No	No	Yes
No	No	No	Yes
No	No	No	Yes
No	No	No	Yes
No	No	No	Yes
No	No	Yes	No
No	No	Yes	No
No	No	Yes	No
No	No	Yes	No
No	No	Yes	No
No	No	Yes	No
No	No	Yes	No
No	No	Yes	No
Yes	No	No	No
Yes	No	No	No
Yes	No	No	No
Yes	No	No	No
Yes	No	No	No
Yes	No	No	No
Yes	No	No	No
Yes	No	No	No
Yes	No	No	No
Yes	No	No	No
Yes	No	Yes	No
Yes	No	Yes	No
Yes	No	Yes	No
Yes	No	Yes	No
Yes	No	Yes	No
Yes	No	Yes	No
Yes	No	Yes	No
Yes	No	No	No
Yes	No	No	No

Yes	No	No	No
Yes	No	No	No
Yes	No	No	No
Yes	No	No	No
Yes	No	No	No
Yes	No	No	No
Yes	No	No	No
Yes	No	No	No
Yes	No	No	No
Yes	No	No	No
Yes	No	No	No
Yes	No	Yes	No
Yes	No	Yes	No
Yes	No	Yes	No
Yes	No	Yes	No
Yes	No	Yes	No
Yes	No	Yes	No
No	No	Yes	No
No	No	Yes	No
No	No	Yes	No
No	No	Yes	No
No	No	Yes	No
Yes	No	Yes	Yes
Yes	No	Yes	Yes
Yes	No	Yes	Yes
Yes	No	Yes	Yes
Yes	No	Yes	Yes
Yes	No	No	No
Yes	No	No	No
Yes	No	No	No
Yes	No	No	No
Yes	No	No	No
Yes	No	No	No
No	No	No	No
No	No	No	No
No	No	No	No
No	No	No	No
No	No	No	No
No	No	No	No
No	No	Yes	No
No	No	Yes	No
No	No	Yes	No
No	No	Yes	No
Yes	No	No	No
Yes	No	No	No
Yes	No	No	No
Yes	No	No	No
Yes	No	No	No
Yes	No	No	No
No	No	No	No

No	No	No	No
No	No	No	No
No	No	No	No
No	No	No	No
No	No	No	No
No	No	No	No
No	No	No	No
No	No	No	No
No	No	No	No
No	No	No	No
No	No	No	No
No	No	No	No
No	No	No	No

Presence of dogs	Presence of cats	Number of cats	Access of cats to feed
Yes	Yes	(3-4)	No
Yes	Yes	(3-4)	No
Yes	Yes	(3-4)	No
Yes	Yes	(3-4)	No
Yes	Yes	(3-4)	No
Yes	Yes	(3-4)	No
Yes	Yes	(3-4)	No
Yes	Yes	(3-4)	No
Yes	Yes	(3-4)	No
Yes	Yes	(3-4)	No
Yes	Yes	(3-4)	No
Yes	Yes	(3-4)	No
Yes	Yes	(1-2)	Yes
Yes	Yes	(1-2)	Yes
Yes	Yes	(1-2)	Yes
Yes	Yes	(1-2)	Yes
Yes	Yes	(1-2)	Yes
Yes	No	0	No
Yes	No	0	No
Yes	No	0	No
Yes	No	0	No
Yes	No	0	No
Yes	No	0	No
Yes	No	0	No
Yes	No	0	No
Yes	Yes	(1-2)	Yes
Yes	Yes	(1-2)	Yes
Yes	Yes	(1-2)	Yes
Yes	Yes	(1-2)	Yes
Yes	Yes	(1-2)	Yes
Yes	Yes	(1-2)	Yes
Yes	Yes	(1-2)	Yes
Yes	Yes	(1-2)	Yes
Yes	Yes	(1-2)	Yes
Yes	Yes	(1-2)	Yes
Yes	Yes	(1-2)	Yes
Yes	Yes	(3-4)	Yes
Yes	Yes	(3-4)	Yes
Yes	Yes	(3-4)	Yes
Yes	Yes	(3-4)	Yes
Yes	Yes	(3-4)	Yes
Yes	Yes	(3-4)	Yes
Yes	Yes	(3-4)	Yes
Yes	Yes	(3-4)	Yes
Yes	Yes	(3-4)	Yes
No	Yes	(1-2)	Yes
No	Yes	(1-2)	Yes

No	Yes	(1-2)	Yes
No	Yes	(1-2)	Yes
No	Yes	(1-2)	Yes
No	Yes	(1-2)	Yes
No	Yes	(1-2)	Yes
No	Yes	(1-2)	Yes
No	Yes	(1-2)	Yes
No	Yes	(1-2)	Yes
Yes	No	0	No
Yes	No	0	No
Yes	No	0	No
Yes	No	0	No
Yes	No	0	No
Yes	No	0	No
Yes	No	0	No
Yes	No	0	No
Yes	No	0	No
Yes	No	0	No
Yes	No	0	No
Yes	No	0	No
Yes	No	0	No
Yes	No	0	No
Yes	No	0	No
Yes	No	0	No
No	Yes	(3-4)	Yes
No	Yes	(3-4)	Yes
No	Yes	(3-4)	Yes
No	Yes	(3-4)	Yes
No	Yes	(3-4)	Yes
No	Yes	(3-4)	Yes
No	Yes	(3-4)	Yes
No	Yes	(3-4)	No
No	Yes	(3-4)	No
No	Yes	(3-4)	No
No	Yes	(3-4)	No
No	Yes	(3-4)	No
No	Yes	(3-4)	No
No	Yes	(3-4)	No
No	Yes	(3-4)	No
Yes	Yes	(1-2)	No
Yes	Yes	(1-2)	No
Yes	Yes	(1-2)	No
Yes	Yes	(1-2)	No
Yes	Yes	(1-2)	No
Yes	Yes	(1-2)	No
Yes	Yes	(1-2)	No
Yes	Yes	(1-2)	No
No	No	0	No
No	No	0	No
No	No	0	No

Yes	Yes	(+5)	Yes
Yes	Yes	(+5)	Yes
Yes	Yes	(+5)	Yes
Yes	Yes	(+5)	Yes
Yes	Yes	(+5)	Yes
Yes	Yes	(+5)	Yes
Yes	Yes	(+5)	Yes
Yes	Yes	(+5)	Yes
No	Yes	(1-2)	No
No	Yes	(1-2)	No
No	Yes	(1-2)	No
No	Yes	(1-2)	No
No	Yes	(1-2)	No
Yes	No	0	No
Yes	No	0	No
Yes	No	0	No
Yes	No	0	No
Yes	No	0	No
Yes	Yes	(1-2)	No
Yes	Yes	(1-2)	No
Yes	Yes	(1-2)	No
Yes	Yes	(1-2)	No
Yes	Yes	(1-2)	No
Yes	Yes	(1-2)	Yes
Yes	Yes	(1-2)	Yes
Yes	Yes	(1-2)	Yes
Yes	Yes	(1-2)	Yes
Yes	Yes	(1-2)	Yes
Yes	Yes	(1-2)	Yes
Yes	Yes	(3-4)	No
Yes	Yes	(3-4)	No
Yes	Yes	(3-4)	No
Yes	Yes	(3-4)	No
Yes	Yes	(3-4)	No
Yes	Yes	(1-2)	Yes
Yes	Yes	(1-2)	Yes
Yes	Yes	(1-2)	Yes
Yes	Yes	(1-2)	Yes
Yes	Yes	(1-2)	Yes
Yes	Yes	(1-2)	Yes
Yes	No	0	No
Yes	No	0	No
Yes	No	0	No
Yes	No	0	No
Yes	No	0	No
Yes	Yes	(3-4)	Yes
Yes	Yes	(3-4)	Yes
Yes	Yes	(3-4)	Yes
Yes	Yes	(3-4)	Yes
Yes	Yes	(3-4)	Yes

Yes	Yes	(3-4)	Yes
Yes	Yes	(3-4)	Yes
Yes	No	0	No
Yes	No	0	No
Yes	No	0	No
Yes	No	0	No
Yes	Yes	(1-2)	No
Yes	Yes	(1-2)	No
Yes	Yes	(1-2)	No
Yes	Yes	(1-2)	No
Yes	Yes	(1-2)	No
Yes	Yes	(1-2)	No
Yes	Yes	(1-2)	No
Yes	Yes	(1-2)	No
Yes	Yes	(3-4)	No
Yes	Yes	(3-4)	No
Yes	Yes	(3-4)	No
Yes	Yes	(3-4)	No
Yes	Yes	(3-4)	No
Yes	Yes	(3-4)	No
Yes	Yes	(3-4)	No
Yes	Yes	(3-4)	No
Yes	Yes	(3-4)	No
No	No	0	No
No	No	0	No
No	No	0	No
No	No	0	No
No	No	0	No
No	No	0	No
Yes	Yes	(1-2)	Yes
Yes	Yes	(1-2)	Yes
Yes	Yes	(1-2)	Yes
Yes	Yes	(1-2)	Yes
Yes	Yes	(1-2)	Yes
Yes	Yes	(3-4)	No
Yes	Yes	(3-4)	No
Yes	Yes	(3-4)	No
Yes	Yes	(3-4)	No
Yes	Yes	(3-4)	No
Yes	Yes	(3-4)	No
Yes	Yes	(1-2)	No
Yes	Yes	(1-2)	No
Yes	Yes	(1-2)	No
Yes	Yes	(1-2)	No
Yes	Yes	(1-2)	No
Yes	Yes	0	No
Yes	Yes	0	No
Yes	Yes	0	No
Yes	Yes	0	No
Yes	Yes	(1-2)	No

Yes	Yes	(1-2)	No
Yes	Yes	(1-2)	No
Yes	Yes	(1-2)	No
Yes	Yes	(1-2)	No
Yes	Yes	(1-2)	No
Yes	Yes	(1-2)	No
Yes	Yes	(1-2)	No
Yes	Yes	(1-2)	No
Yes	No	0	No
Yes	No	0	No
Yes	No	0	No
Yes	No	0	No
Yes	No	0	No
Yes	No	0	No
Yes	No	0	No
Yes	Yes	(1-2)	No
Yes	Yes	(1-2)	No
Yes	Yes	(1-2)	No
Yes	Yes	(1-2)	No
Yes	Yes	(1-2)	No
Yes	Yes	(1-2)	No
Yes	Yes	(1-2)	Yes
Yes	Yes	(1-2)	Yes
Yes	Yes	(1-2)	Yes
Yes	Yes	(1-2)	Yes
Yes	Yes	(1-2)	Yes
Yes	Yes	(1-2)	Yes
Yes	Yes	(1-2)	Yes
Yes	Yes	(3-4)	No
Yes	Yes	(3-4)	No
Yes	Yes	(3-4)	No
Yes	Yes	(3-4)	No
Yes	Yes	(3-4)	No
No	Yes	(3-4)	No
No	No	0	No
No	No	0	No
No	No	0	No
No	No	0	No
No	No	0	No
No	No	0	No
No	Yes	(1-2)	Yes
No	Yes	(1-2)	Yes
No	Yes	(1-2)	Yes
No	Yes	(1-2)	Yes
Yes	No	0	No
Yes	No	0	No
Yes	No	0	No
Yes	No	0	No
Yes	No	0	No
Yes	Yes	(1-2)	Yes

Yes	Yes	(1-2)	Yes
Yes	Yes	(1-2)	Yes
Yes	Yes	(1-2)	Yes
Yes	Yes	(1-2)	Yes
Yes	Yes	(1-2)	Yes
Yes	Yes	(1-2)	Yes
Yes	No	0	No
Yes	No	0	No
Yes	No	0	No
Yes	No	0	No
Yes	No	0	No
Yes	No	0	No
Yes	No	0	No
Yes	Yes	(3-4)	Yes
Yes	Yes	(3-4)	Yes
Yes	Yes	(3-4)	Yes
Yes	Yes	(3-4)	Yes
Yes	Yes	(3-4)	Yes
Yes	Yes	(3-4)	Yes
Yes	Yes	(3-4)	Yes
Yes	Yes	(3-4)	Yes
Yes	Yes	(3-4)	Yes
Yes	No	0	No
Yes	No	0	No
Yes	No	0	No
Yes	No	0	No
Yes	No	0	No
Yes	No	0	No
Yes	No	0	No
No	No	0	No
No	No	0	No
No	No	0	No
No	No	0	No
No	No	0	No
No	No	0	No
Yes	Yes	(1-2)	No
Yes	Yes	(1-2)	No
Yes	Yes	(1-2)	No
Yes	Yes	(1-2)	No
Yes	Yes	(1-2)	No
Yes	Yes	(1-2)	No
Yes	Yes	(1-2)	No
Yes	Yes	(1-2)	No
Yes	Yes	(1-2)	No
Yes	Yes	(3-4)	No
Yes	Yes	(3-4)	No
Yes	Yes	(3-4)	No
Yes	Yes	(3-4)	No
Yes	Yes	(3-4)	No
Yes	Yes	(3-4)	No

Yes	Yes	(1-2)	Yes
Yes	Yes	(1-2)	Yes
Yes	Yes	(1-2)	Yes
Yes	Yes	(1-2)	Yes
Yes	Yes	(1-2)	Yes
Yes	Yes	(1-2)	Yes
No	Yes	(1-2)	No
No	Yes	(1-2)	No
No	Yes	(1-2)	No
No	Yes	(1-2)	No
No	Yes	(1-2)	No
No	Yes	(1-2)	No
No	Yes	(1-2)	No
No	Yes	(3-4)	Yes
No	Yes	(3-4)	Yes
No	Yes	(3-4)	Yes
No	Yes	(3-4)	Yes
No	Yes	(3-4)	Yes
No	Yes	(1-2)	No
No	Yes	(1-2)	No
No	Yes	(1-2)	No
Yes	Yes	(3-4)	Yes
Yes	Yes	(3-4)	Yes
Yes	Yes	(3-4)	Yes
Yes	Yes	(3-4)	Yes
Yes	Yes	(3-4)	Yes
Yes	Yes	(3-4)	Yes
Yes	Yes	(3-4)	Yes
Yes	Yes	(3-4)	Yes
Yes	Yes	(3-4)	Yes
Yes	Yes	(3-4)	Yes
Yes	Yes	(3-4)	Yes
Yes	Yes	(3-4)	Yes
Yes	Yes	(3-4)	Yes
Yes	Yes	(1-2)	No
Yes	Yes	(1-2)	No
Yes	Yes	(1-2)	No
Yes	Yes	(1-2)	No
Yes	Yes	(1-2)	No
Yes	Yes	(1-2)	No
Yes	Yes	(1-2)	No
Yes	Yes	(1-2)	No
Yes	Yes	(1-2)	No
Yes	Yes	(1-2)	No
Yes	Yes	(1-2)	No
Yes	Yes	(3-4)	Yes
Yes	Yes	(3-4)	Yes
Yes	Yes	(3-4)	Yes
Yes	Yes	(3-4)	Yes
Yes	No	0	No

Yes	No	0	No
Yes	No	0	No
Yes	No	0	No
Yes	No	0	No
Yes	Yes	(1-2)	No
Yes	Yes	(1-2)	No
Yes	Yes	(1-2)	No
Yes	Yes	(1-2)	No
Yes	Yes	(1-2)	No
Yes	Yes	(1-2)	No
Yes	Yes	(1-2)	No
Yes	Yes	(1-2)	No
Yes	Yes	(1-2)	No
Yes	Yes	(1-2)	No
Yes	Yes	(1-2)	No
Yes	Yes	(1-2)	No
Yes	Yes	(3-4)	No
Yes	Yes	(3-4)	No
Yes	Yes	(3-4)	No
Yes	Yes	(3-4)	No
Yes	Yes	(3-4)	No
Yes	Yes	(3-4)	No
Yes	Yes	(3-4)	No
Yes	Yes	(1-2)	No
Yes	Yes	(1-2)	No
Yes	Yes	(1-2)	No
Yes	Yes	(1-2)	No
Yes	Yes	(1-2)	No
Yes	Yes	(1-2)	No
Yes	Yes	(1-2)	No
Yes	Yes	(1-2)	No
Yes	Yes	(1-2)	No
Yes	Yes	(1-2)	No
Yes	Yes	(1-2)	No
Yes	Yes	(1-2)	No
Yes	Yes	(1-2)	No
Yes	Yes	(1-2)	No
Yes	Yes	(1-2)	No
Yes	Yes	(1-2)	No
Yes	Yes	(1-2)	No
Yes	Yes	(1-2)	No
Yes	Yes	(1-2)	No
Yes	Yes	(1-2)	No
Yes	Yes	(1-2)	No
Yes	Yes	(1-2)	No
Yes	Yes	(1-2)	No
Yes	Yes	(1-2)	No
Yes	Yes	(1-2)	No
Yes	Yes	(1-2)	No
Yes	Yes	(1-2)	No

Yes	Yes	(1-2)	No
Yes	Yes	(1-2)	No
Yes	Yes	(1-2)	No
Yes	Yes	(1-2)	No
Yes	Yes	(1-2)	No
Yes	Yes	(1-2)	No
Yes	Yes	(1-2)	No
Yes	Yes	(1-2)	No
Yes	Yes	(1-2)	No
Yes	Yes	(1-2)	No
Yes	Yes	(1-2)	No
Yes	Yes	(3-4)	No
Yes	Yes	(3-4)	No
Yes	Yes	(3-4)	No
Yes	Yes	(3-4)	No
Yes	Yes	(3-4)	No
Yes	Yes	(3-4)	No
Yes	Yes	(+5)	Yes
Yes	Yes	(+5)	Yes
Yes	Yes	(+5)	Yes
Yes	Yes	(+5)	Yes
Yes	Yes	(+5)	Yes
Yes	Yes	(3-4)	Yes
Yes	Yes	(3-4)	Yes
Yes	Yes	(3-4)	Yes
Yes	Yes	(3-4)	Yes
Yes	Yes	(3-4)	Yes
Yes	Yes	(1-2)	No
Yes	Yes	(1-2)	No
Yes	Yes	(1-2)	No
Yes	Yes	(1-2)	No
Yes	Yes	(1-2)	No
Yes	Yes	(1-2)	No
No	Yes	(1-2)	No
No	Yes	(1-2)	No
No	Yes	(1-2)	No
No	Yes	(1-2)	No
No	Yes	(1-2)	No
No	Yes	(1-2)	No
Yes	No	0	No
Yes	No	0	No
Yes	No	0	No
Yes	No	0	No
Yes	Yes	(3-4)	Yes
Yes	Yes	(3-4)	Yes
Yes	Yes	(3-4)	Yes
Yes	Yes	(3-4)	Yes
Yes	Yes	(3-4)	Yes
Yes	Yes	(3-4)	Yes
Yes	Yes	(1-2)	No

Yes	Yes	(1-2)	No
Yes	Yes	(1-2)	No
Yes	Yes	(1-2)	No
Yes	Yes	(1-2)	No
Yes	Yes	(1-2)	No
Yes	Yes	(1-2)	No
Yes	Yes	(1-2)	No
Yes	Yes	(1-2)	No
Yes	Yes	(1-2)	No
Yes	Yes	(1-2)	No
Yes	Yes	(1-2)	No
Yes	Yes	(1-2)	No
Yes	Yes	(1-2)	No

Access of cats to water	Cats for control of rodents	Control of cat’s pop	Cats consume plac
Yes	Yes	No	Yes
Yes	Yes	No	Yes
Yes	Yes	No	Yes
Yes	Yes	No	Yes
Yes	Yes	No	Yes
Yes	Yes	No	Yes
Yes	Yes	No	Yes
Yes	Yes	No	Yes
Yes	Yes	No	Yes
Yes	Yes	No	Yes
Yes	Yes	No	Yes
Yes	Yes	No	Yes
No	No	No	No
No	No	No	No
No	No	No	No
No	No	No	No
No	No	No	No
No	No		
No	No		
No	No		
No	No		
No	No		
No	No		
No	No		
No	No		
Yes	No	No	Yes
Yes	No	No	Yes
Yes	No	No	Yes
Yes	No	No	Yes
Yes	No	No	Yes
Yes	No	No	Yes
Yes	No	No	Yes
Yes	No	No	No
Yes	No	No	No
Yes	No	No	No
Yes	No	No	No
No	No	No	Yes
No	No	No	Yes
No	No	No	Yes
No	No	No	Yes
No	No	No	Yes
No	No	No	Yes
No	No	No	Yes
No	No	No	Yes
No	No	No	Yes
Yes	Yes	No	No
Yes	Yes	No	No

Yes	Yes	No	No
Yes	Yes	No	No
Yes	Yes	No	No
Yes	Yes	No	No
Yes	Yes	No	No
Yes	Yes	No	No
Yes	Yes	No	No
Yes	Yes	No	No
No	No		
No	No		
No	No		
No	No		
No	No		
No	No		
No	No		
No	No		
No	No		
No	No		
No	No		
No	No		
No	No		
No	No		
No	No		
No	No		
Yes	Yes	Yes	Yes
Yes	Yes	Yes	Yes
Yes	Yes	Yes	Yes
Yes	Yes	Yes	Yes
Yes	Yes	Yes	Yes
Yes	Yes	Yes	Yes
Yes	Yes	Yes	Yes
Yes	No	No	Yes
Yes	No	No	Yes
Yes	No	No	Yes
Yes	No	No	Yes
Yes	No	No	Yes
Yes	No	No	Yes
Yes	No	No	Yes
Yes	No	No	Yes
Yes	Yes	No	Yes
Yes	Yes	No	Yes
Yes	Yes	No	Yes
Yes	Yes	No	Yes
Yes	Yes	No	Yes
Yes	Yes	No	Yes
Yes	Yes	No	Yes
Yes	Yes	No	Yes
No	No		
No	No		
No	No		

Yes	Yes	No	Yes
Yes	Yes	No	Yes
Yes	Yes	No	Yes
Yes	Yes	No	Yes
Yes	Yes	No	Yes
Yes	Yes	No	Yes
Yes	Yes	No	Yes
Yes	Yes	No	Yes
Yes	Yes	Yes	Yes
Yes	Yes	Yes	Yes
Yes	Yes	Yes	Yes
Yes	Yes	Yes	Yes
Yes	Yes	Yes	Yes
No	No		
No	No		
No	No		
No	No		
No	No		
Yes	No	No	No
Yes	No	No	No
Yes	No	No	No
Yes	No	No	No
Yes	No	No	No
Yes	Yes	No	No
Yes	Yes	No	No
Yes	Yes	No	No
Yes	Yes	No	No
Yes	Yes	No	No
Yes	Yes	No	No
Yes	No	No	Yes
Yes	No	No	Yes
Yes	No	No	Yes
Yes	No	No	Yes
Yes	No	No	Yes
No	No	No	Yes
No	No	No	Yes
No	No	No	Yes
No	No	No	Yes
No	No	No	Yes
No	No	No	Yes
No	No		
No	No		
No	No		
No	No		
No	No		
No	No	No	Yes
No	No	No	Yes
No	No	No	Yes
No	No	No	Yes
No	No	No	Yes

No	No	No	Yes
No	No	No	Yes
No	No		
No	No		
No	No		
No	No		
Yes	No	No	Yes
Yes	No	No	Yes
Yes	No	No	Yes
Yes	No	No	Yes
No	No	No	No
No	No	No	No
No	No	No	No
No	No	No	No
Yes	No	No	Yes
Yes	No	No	Yes
Yes	No	No	Yes
Yes	No	No	Yes
Yes	No	No	Yes
Yes	No	No	Yes
Yes	No	No	Yes
Yes	No	No	Yes
Yes	No	No	Yes
No	No		
No	No		
No	No		
No	No		
No	No		
No	No		
Yes	No	No	No
Yes	No	No	No
Yes	No	No	No
Yes	No	No	No
Yes	No	No	No
Yes	Yes	No	Yes
Yes	Yes	No	Yes
Yes	Yes	No	Yes
Yes	Yes	No	Yes
Yes	Yes	No	Yes
Yes	Yes	No	Yes
No	Yes	No	Yes
No	Yes	No	Yes
No	Yes	No	Yes
No	Yes	No	Yes
No	Yes	No	Yes
No	No		
No	No		
No	No		
No	No		
Yes	No	No	Yes

Yes	No	No	Yes
Yes	No	No	Yes
Yes	No	No	Yes
Yes	No	No	Yes
Yes	No	No	Yes
Yes	No	No	Yes
Yes	No	No	Yes
Yes	No	No	Yes
No	No		
No	No		
No	No		
No	No		
No	No		
No	No		
No	No		
Yes	No	No	Yes
Yes	No	No	Yes
Yes	No	No	Yes
Yes	No	No	Yes
Yes	No	No	Yes
Yes	No	No	Yes
No	Yes	No	No
No	Yes	No	No
No	Yes	No	No
No	Yes	No	No
No	Yes	No	No
No	Yes	No	No
No	Yes	No	No
Yes	Yes	Yes	Yes
Yes	Yes	Yes	Yes
Yes	Yes	Yes	Yes
Yes	Yes	Yes	Yes
Yes	Yes	Yes	Yes
Yes	Yes	Yes	Yes
No	No		
No	No		
No	No		
No	No		
No	No		
No	No		
No	No	No	No
No	No	No	No
No	No	No	No
No	No	No	No
No	No		
No	No		
No	No		
No	No		
No	No		
No	Yes	No	Yes

No	Yes	No	Yes
No	Yes	No	Yes
No	Yes	No	Yes
No	Yes	No	Yes
No	Yes	No	Yes
No	Yes	No	Yes
No	No		
No	No		
No	No		
No	No		
No	No		
No	No		
No	No		
Yes	No	No	Yes
Yes	No	No	Yes
Yes	No	No	Yes
Yes	No	No	Yes
Yes	No	No	Yes
Yes	No	No	Yes
Yes	No	No	Yes
Yes	No	No	Yes
Yes	No	No	Yes
No	No		
No	No		
No	No		
No	No		
No	No		
No	No		
No	No		
No	No		
No	No		
No	No		
No	No		
No	No		
No	No		
Yes	Yes	No	Yes
Yes	Yes	No	Yes
Yes	Yes	No	Yes
Yes	Yes	No	Yes
Yes	Yes	No	Yes
Yes	Yes	No	Yes
Yes	Yes	No	Yes
Yes	Yes	No	Yes
Yes	Yes	No	Yes
Yes	No	No	Yes
Yes	No	No	Yes
Yes	No	No	Yes
Yes	No	No	Yes
Yes	No	No	Yes
Yes	No	No	Yes

Yes	No	No	No
Yes	No	No	No
Yes	No	No	No
Yes	No	No	No
Yes	No	No	No
Yes	No	No	No
Yes	No	No	Yes
Yes	No	No	Yes
Yes	No	No	Yes
Yes	No	No	Yes
Yes	No	No	Yes
Yes	No	No	Yes
Yes	No	No	Yes
No	Yes	No	No
No	Yes	No	No
No	Yes	No	No
No	Yes	No	No
No	Yes	No	No
Yes	No	No	No
Yes	No	No	No
Yes	No	No	No
Yes	No	No	Yes
Yes	No	No	Yes
Yes	No	No	Yes
Yes	No	No	Yes
Yes	No	No	Yes
Yes	No	No	Yes
No	No	No	No
No	No	No	No
No	No	No	No
No	No	No	No
No	No	No	No
No	No	No	No
No	No	No	No
Yes	No	No	Yes
Yes	No	No	Yes
Yes	No	No	Yes
Yes	No	No	Yes
Yes	No	No	Yes
Yes	No	No	Yes
Yes	No	No	Yes
Yes	No	No	No
Yes	No	No	No
Yes	No	No	No
Yes	No	No	No
Yes	No	No	Yes
Yes	No	No	Yes
Yes	No	No	Yes
Yes	No	No	Yes
No	No		

No	No		
No	No		
No	No		
No	No		
Yes	No	No	Yes
Yes	No	No	Yes
Yes	No	No	Yes
Yes	No	No	Yes
Yes	No	No	Yes
Yes	No	No	Yes
Yes	No	No	No
Yes	Yes	No	No
Yes	Yes	No	No
Yes	Yes	No	No
Yes	Yes	No	No
Yes	Yes	No	No
Yes	Yes	No	Yes
Yes	Yes	No	Yes
Yes	Yes	No	Yes
Yes	Yes	No	Yes
Yes	Yes	No	Yes
Yes	Yes	No	Yes
Yes	Yes	No	Yes
Yes	No	No	No
Yes	No	No	No
Yes	No	No	No
Yes	No	No	No
Yes	No	No	No
Yes	No	No	No
Yes	No	No	No
Yes	No	No	No
Yes	No	No	No
Yes	No	No	No
Yes	No	No	No
Yes	No	No	No
Yes	No	No	No
Yes	No	No	No
Yes	No	No	No
Yes	No	No	No
Yes	No	No	No
Yes	No	No	No
Yes	No	No	Yes
Yes	No	No	Yes
Yes	No	No	Yes
Yes	No	No	Yes
Yes	No	No	Yes
Yes	No	No	Yes
Yes	No	No	Yes
No	No	No	Yes
No	No	No	Yes

No	No	No	Yes
No	No	No	Yes
No	No	No	Yes
No	No	No	Yes
No	No	No	Yes
No	No	No	Yes
No	No	No	Yes
No	No	No	Yes
No	No	No	Yes
No	No	No	Yes
No	No	No	Yes
Yes	No	No	No
Yes	No	No	No
Yes	No	No	No
Yes	No	No	No
Yes	No	No	No
Yes	No	No	No
Yes	No	No	No
Yes	No	No	No
Yes	No	No	No
Yes	No	No	No
Yes	No	No	No
Yes	Yes	No	No
Yes	Yes	No	No
Yes	Yes	No	No
Yes	Yes	No	No
Yes	Yes	No	No
No	No	No	No
No	No	No	No
No	No	No	No
No	No	No	No
No	No	No	No
No	No	No	No
Yes	No	No	No
Yes	No	No	No
Yes	No	No	No
Yes	No	No	No
Yes	No	No	No
Yes	No	No	No
No	No		
No	No		
No	No		
No	No		
Yes	No	No	Yes
Yes	No	No	Yes
Yes	No	No	Yes
Yes	No	No	Yes
Yes	No	No	Yes
Yes	No	No	Yes
Yes	No	No	No

Yes	No	No	No
Yes	No	No	No
Yes	No	No	No
Yes	No	No	No
Yes	No	No	No
Yes	No	No	No
Yes	No	No	No
No	No	No	No
No	No	No	No
No	No	No	No
No	No	No	No
No	No	No	No
No	No	No	No

Birth of cats in the farm	Presence of wild cats	Hygiene	Slaughtering in the farm
Yes	No	Good	No
Yes	No	Good	No
Yes	No	Good	No
Yes	No	Good	No
Yes	No	Good	No
Yes	No	Good	No
Yes	No	Good	No
Yes	No	Good	No
Yes	No	Good	No
Yes	No	Good	No
Yes	No	Good	No
Yes	No	Good	No
No	No	Good	No
No	No	Good	No
No	No	Good	No
No	No	Good	No
No	No	Good	No
No	No	Good	No
No	No	Good	No
No	No	Good	No
No	No	Good	No
No	No	Good	No
No	No	Good	No
No	No	Good	No
No	No	Good	No
No	No	Good	No
No	No	Good	No
No	No	Good	No
No	No	Good	No
No	No	Good	No
No	No	Good	No
No	No	Good	No
No	No	Good	No
No	No	Good	No
No	No	Good	No
No	No	Good	No
Yes	No	Good	No
Yes	No	Good	No
Yes	No	Good	No
Yes	No	Good	No
Yes	No	Good	No
Yes	No	Good	No
Yes	No	Good	No
Yes	No	Good	No
Yes	No	Good	No
Yes	No	Bad	No
Yes	No	Bad	No

Yes	No	Bad	No
Yes	No	Bad	No
Yes	No	Bad	No
Yes	No	Bad	No
Yes	No	Bad	No
Yes	No	Bad	No
Yes	No	Bad	No
Yes	No	Bad	No
No	No	Good	No
No	No	Good	No
No	No	Good	No
No	No	Good	No
No	No	Good	No
No	No	Good	No
No	No	Good	No
No	No	Good	No
No	No	Good	No
No	No	Good	No
No	No	Good	No
No	Yes	Good	No
No	Yes	Good	No
No	Yes	Good	No
No	Yes	Good	No
No	Yes	Good	No
Yes	Yes	Bad	Yes
Yes	Yes	Bad	Yes
Yes	Yes	Bad	Yes
Yes	Yes	Bad	Yes
Yes	Yes	Bad	Yes
Yes	Yes	Bad	Yes
Yes	Yes	Bad	Yes
No	No	Good	No
No	No	Good	No
No	No	Good	No
No	No	Good	No
No	No	Good	No
No	No	Good	No
No	No	Good	No
No	No	Good	No
No	No	Bad	No
No	No	Bad	No
No	No	Bad	No
No	No	Bad	No
No	No	Bad	No
No	No	Bad	No
No	No	Bad	No
No	No	Bad	No
No	No	Good	No
No	No	Good	No
No	No	Good	No

No	No	Good	No
No	No	Good	No
No	No	Good	No
No	No	Good	No
No	No	Good	No
No	No	Good	No
No	No	Good	No
No	No	Good	No
Yes	No	Good	No
Yes	No	Good	No
Yes	No	Good	No
Yes	No	Good	No
Yes	No	Good	No
No	Yes	Good	No
No	Yes	Good	No
No	Yes	Good	No
No	Yes	Good	No
No	Yes	Good	No
No	No	Bad	No
No	No	Bad	No
No	No	Bad	No
No	No	Bad	No
No	No	Bad	No
Yes	No	Good	No
Yes	No	Good	No
Yes	No	Good	No
Yes	No	Good	No
Yes	No	Good	No
Yes	No	Good	No
No	No	Bad	Yes
No	No	Bad	Yes
No	No	Bad	Yes
No	No	Bad	Yes
No	No	Bad	Yes
No	No	Bad	No
No	No	Bad	No
No	No	Bad	No
No	No	Bad	No
No	No	Bad	No
No	No	Bad	No
No	No	Good	No
No	No	Good	No
No	No	Good	No
No	No	Good	No
No	No	Good	No
No	No	Good	No
No	No	Good	No
No	No	Good	No
No	No	Good	No
No	No	Good	No

No	No	Good	No
No	No	Good	No
No	No	Bad	No
No	No	Bad	No
No	No	Bad	No
No	No	Bad	No
No	No	Good	No
No	No	Good	No
No	No	Good	No
No	No	Good	No
No	No	Bad	No
No	No	Bad	No
No	No	Bad	No
No	No	Bad	No
No	No	Good	No
No	No	Good	No
No	No	Good	No
No	No	Good	No
No	No	Good	No
No	No	Good	No
No	No	Good	No
No	No	Good	No
No	No	Good	No
No	Yes	Good	No
No	Yes	Good	No
No	Yes	Good	No
No	Yes	Good	No
No	Yes	Good	No
No	Yes	Good	No
No	No	Good	No
No	No	Good	No
No	No	Good	No
No	No	Good	No
No	No	Good	No
Yes	No	Good	No
Yes	No	Good	No
Yes	No	Good	No
Yes	No	Good	No
Yes	No	Good	No
Yes	No	Good	No
No	No	Good	No
No	No	Good	No
No	No	Good	No
No	No	Good	No
No	No	Good	No
No	No	Bad	No
No	No	Bad	No
No	No	Bad	No
No	No	Bad	No
No	No	Good	No

No	No	Good	No
No	No	Good	No
No	No	Good	No
No	No	Good	No
No	No	Good	No
No	No	Good	No
No	No	Good	No
No	No	Good	No
No	Yes	Good	No
No	Yes	Good	No
No	Yes	Good	No
No	Yes	Good	No
No	Yes	Good	No
No	Yes	Good	No
No	Yes	Good	No
No	No	Bad	No
No	No	Bad	No
No	No	Bad	No
No	No	Bad	No
No	No	Bad	No
No	No	Bad	No
No	No	Bad	No
No	No	Bad	No
No	No	Bad	No
No	No	Bad	No
No	No	Bad	No
No	No	Bad	No
No	No	Bad	No
No	No	Good	Yes
No	No	Good	Yes
No	No	Good	Yes
No	No	Good	Yes
No	No	Good	Yes
No	No	Good	Yes
No	No	Bad	Yes
No	No	Bad	Yes
No	No	Bad	Yes
No	No	Bad	Yes
No	No	Bad	Yes
No	No	Bad	Yes
Yes	No	Bad	No
Yes	No	Bad	No
Yes	No	Bad	No
Yes	No	Bad	No
No	No	Good	No
No	No	Good	No
No	No	Good	No
No	No	Good	No
No	No	Good	No
No	No	Good	No

No	No	Good	No
No	No	Good	No
No	No	Good	No
No	No	Good	No
No	No	Good	No
No	No	Good	No
No	No	Good	No
No	No	Good	No
No	No	Good	No
No	No	Good	No
No	No	Good	No
No	No	Good	No
No	No	Good	No
No	Yes	Medium	No
No	Yes	Medium	No
No	Yes	Medium	No
No	Yes	Medium	No
No	Yes	Medium	No
No	Yes	Medium	No
No	Yes	Medium	No
No	Yes	Medium	No
No	Yes	Medium	No
No	No	Good	No
No	No	Good	No
No	No	Good	No
No	No	Good	No
No	No	Good	No
No	No	Good	No
No	No	Good	No
No	No	Bad	No
No	No	Bad	No
No	No	Bad	No
No	No	Bad	No
No	No	Bad	No
No	No	Bad	No
Yes	No	Good	No
Yes	No	Good	No
Yes	No	Good	No
Yes	No	Good	No
Yes	No	Good	No
Yes	No	Good	No
Yes	No	Good	No
Yes	No	Good	No
Yes	No	Good	No
Yes	No	Good	No
Yes	No	Good	No
Yes	No	Good	No
Yes	No	Good	No
Yes	No	Good	No
Yes	No	Good	No

No	No	Good	No
No	No	Good	No
No	No	Good	No
No	No	Good	No
No	No	Good	No
No	No	Good	No
No	No	Good	No
No	No	Good	No
No	No	Good	No
No	No	Good	No
No	No	Good	No
No	No	Good	No
No	No	Good	No
No	No	Bad	No
No	No	Bad	No
No	No	Bad	No
No	No	Bad	No
No	No	Bad	No
No	No	Good	No
No	No	Good	No
No	No	Good	No
No	Yes	Bad	Yes
No	Yes	Bad	Yes
No	Yes	Bad	Yes
No	Yes	Bad	Yes
No	Yes	Bad	Yes
No	Yes	Bad	Yes
Yes	No	Good	No
Yes	No	Good	No
Yes	No	Good	No
Yes	No	Good	No
Yes	No	Good	No
Yes	No	Good	No
Yes	No	Good	No
Yes	No	Good	No
Yes	No	Good	No
Yes	No	Good	No
Yes	No	Good	No
Yes	No	Good	No
Yes	No	Good	No
Yes	No	Good	No
No	No	Bad	No
No	No	Bad	No
No	No	Bad	No
No	No	Bad	No
No	No	Good	No
No	No	Good	No
No	No	Good	No
No	No	Good	No
No	Yes	Bad	No

No	Yes	Bad	No
No	Yes	Bad	No
No	Yes	Bad	No
No	Yes	Bad	No
No	No	Good	No
No	No	Good	No
No	No	Good	No
No	No	Good	No
No	No	Good	No
No	No	Good	No
No	No	Good	No
No	No	Good	No
No	No	Good	No
No	No	Good	No
No	No	Good	No
No	No	Good	No
Yes	No	Good	No
Yes	No	Good	No
Yes	No	Good	No
Yes	No	Good	No
Yes	No	Good	No
Yes	No	Good	No
Yes	No	Good	No
No	No	Good	No
No	No	Good	No
No	No	Good	No
No	No	Good	No
No	No	Good	No
No	No	Good	No
No	No	Good	No
No	No	Good	No
Yes	No	Good	No
Yes	No	Good	No
Yes	No	Good	No
Yes	No	Good	No
Yes	No	Good	No
Yes	No	Good	No
Yes	No	Good	No
Yes	No	Good	No
Yes	No	Good	No
Yes	No	Good	No
No	No	Good	No
No	No	Good	No
No	No	Good	No
No	No	Good	No
No	No	Good	No
No	No	Good	No
No	No	Good	No
No	No	Medium	No
No	No	Medium	No

No	No	Medium	No
No	No	Medium	No
No	No	Medium	No
No	No	Medium	No
No	No	Medium	No
No	No	Medium	No
No	No	Medium	No
No	No	Medium	No
No	No	Medium	No
No	No	Medium	No
No	No	Medium	No
No	No	Good	No
No	No	Good	No
No	No	Good	No
No	No	Good	No
No	No	Good	No
No	No	Good	No
Yes	No	Medium	No
Yes	No	Medium	No
Yes	No	Medium	No
Yes	No	Medium	No
Yes	No	Medium	No
No	No	Medium	No
No	No	Medium	No
No	No	Medium	No
No	No	Medium	No
No	No	Medium	No
No	No	Medium	No
No	No	Medium	No
No	No	Medium	No
No	No	Medium	No
No	No	Medium	No
No	No	Medium	No
Yes	No	Medium	No
Yes	No	Medium	No
Yes	No	Medium	No
Yes	No	Medium	No
Yes	No	Medium	No
Yes	No	Medium	No
No	Yes	Medium	No
No	Yes	Medium	No
No	Yes	Medium	No
No	Yes	Medium	No
Yes	No	Medium	No
Yes	No	Medium	No
Yes	No	Medium	No
Yes	No	Medium	No
Yes	No	Medium	No
Yes	No	Medium	No
No	No	Medium	No

No	No	Medium	No
No	No	Medium	No
No	No	Medium	No
No	No	Medium	No
No	No	Medium	No
No	No	Medium	No
No	No	Medium	No
No	No	Good	No
No	No	Good	No
No	No	Good	No
No	No	Good	No
No	No	Good	No
No	No	Good	No

Quarantine	Special place for parturition	Exchange of breeder	Use of anthelminthic
Yes	Yes	No	No
Yes	Yes	No	No
Yes	Yes	No	No
Yes	Yes	No	No
Yes	Yes	No	No
Yes	Yes	No	No
Yes	Yes	No	No
Yes	Yes	No	No
Yes	Yes	No	No
Yes	Yes	No	No
Yes	Yes	No	No
Yes	Yes	No	No
Yes	No	No	Yes
Yes	No	No	Yes
Yes	No	No	Yes
Yes	No	No	Yes
Yes	No	No	Yes
Yes	Yes	No	Yes
Yes	Yes	No	Yes
Yes	Yes	No	Yes
Yes	Yes	No	Yes
Yes	Yes	No	Yes
Yes	Yes	No	Yes
Yes	Yes	No	Yes
Yes	Yes	No	Yes
No	No	Yes	Yes
No	No	Yes	Yes
No	No	Yes	Yes
No	No	Yes	Yes
No	No	Yes	Yes
No	No	Yes	Yes
No	No	Yes	Yes
No	No	No	Yes
No	No	No	Yes
No	No	No	Yes
No	No	No	Yes
No	No	No	No
No	No	No	No
No	No	No	No
No	No	No	No
No	No	No	No
No	No	No	No
No	No	No	No
No	No	No	No
No	No	No	No
No	No	Yes	Yes
No	No	Yes	Yes

No	No	Yes	Yes
No	No	Yes	Yes
No	No	Yes	Yes
No	No	Yes	Yes
No	No	Yes	Yes
No	No	Yes	Yes
No	No	Yes	Yes
No	No	Yes	Yes
No	No	Yes	Yes
No	No	Yes	Yes
No	No	Yes	Yes
No	No	Yes	Yes
No	No	Yes	Yes
No	No	Yes	Yes
No	No	Yes	Yes
No	No	Yes	Yes
No	No	Yes	Yes
No	No	Yes	Yes
No	No	Yes	Yes
Yes	Yes	No	No
Yes	Yes	No	No
Yes	Yes	No	No
Yes	Yes	No	No
Yes	Yes	No	No
No	No	No	Yes
No	No	No	Yes
No	No	No	Yes
No	No	No	Yes
No	No	No	Yes
No	No	No	Yes
No	No	No	Yes
No	No	No	Yes
No	No	No	Yes
No	No	No	Yes
No	No	No	Yes
No	No	No	Yes
No	No	No	Yes
No	No	No	Yes
No	No	No	Yes
No	No	No	No
No	No	No	No
No	No	No	No
No	No	No	No
No	No	No	No
No	No	No	No
No	No	No	No
No	No	No	No
No	No	Yes	Yes
No	No	Yes	Yes
No	No	Yes	Yes

No	No	Yes	Yes
No	No	Yes	Yes
No	No	Yes	Yes
No	No	Yes	Yes
No	No	Yes	Yes
No	No	Yes	Yes
No	No	Yes	Yes
No	No	Yes	Yes
Yes	Yes	Yes	Yes
Yes	Yes	Yes	Yes
Yes	Yes	Yes	Yes
Yes	Yes	Yes	Yes
Yes	Yes	Yes	Yes
Yes	No	No	Yes
Yes	No	No	Yes
Yes	No	No	Yes
Yes	No	No	Yes
Yes	No	No	Yes
No	No	No	Yes
No	No	No	Yes
No	No	No	Yes
No	No	No	Yes
No	No	No	Yes
No	Yes	No	Yes
No	Yes	No	Yes
No	Yes	No	Yes
No	Yes	No	Yes
No	Yes	No	Yes
No	Yes	No	Yes
No	No	No	Yes
No	No	No	Yes
No	No	No	Yes
No	No	No	Yes
No	No	No	Yes
No	No	No	Yes
No	No	No	Yes
No	No	No	Yes
No	No	No	Yes
No	No	No	Yes
No	No	No	Yes
No	No	No	Yes
No	No	No	Yes
No	No	No	Yes
No	No	No	Yes
No	No	No	Yes
No	No	Yes	Yes
No	No	Yes	Yes
No	No	Yes	Yes
No	No	Yes	Yes
No	No	Yes	Yes

No	No	Yes	Yes
No	No	Yes	Yes
No	No	Yes	No
No	No	Yes	No
No	No	Yes	No
No	No	Yes	No
No	No	Yes	Yes
No	No	Yes	Yes
No	No	Yes	Yes
No	No	Yes	Yes
No	No	No	Yes
No	No	No	Yes
No	No	No	Yes
No	No	No	Yes
No	No	No	Yes
No	No	No	Yes
No	No	No	Yes
No	No	No	Yes
No	No	No	Yes
No	No	No	Yes
No	No	No	Yes
No	No	No	Yes
No	No	No	Yes
No	No	Yes	Yes
No	No	Yes	Yes
No	No	Yes	Yes
No	No	Yes	Yes
No	No	Yes	Yes
No	No	Yes	Yes
Yes	No	No	No
Yes	No	No	No
Yes	No	No	No
Yes	No	No	No
Yes	No	No	No
No	No	No	Yes
No	No	No	Yes
No	No	No	Yes
No	No	No	Yes
No	No	No	Yes
No	No	No	Yes
No	No	Yes	Yes
No	No	Yes	Yes
No	No	Yes	Yes
No	No	Yes	Yes
No	No	Yes	Yes
No	No	No	No
No	No	No	No
No	No	No	No
No	No	No	No
No	No	No	Yes

No	No	No	Yes
No	No	No	Yes
No	No	No	Yes
No	No	No	Yes
No	No	No	Yes
No	No	No	Yes
No	No	No	Yes
No	No	No	Yes
Yes	No	No	Yes
Yes	Yes	No	Yes
Yes	Yes	No	Yes
Yes	Yes	No	Yes
Yes	Yes	No	Yes
Yes	No	No	Yes
Yes	No	No	Yes
No	No	Yes	No
No	No	Yes	No
No	No	Yes	No
No	No	Yes	No
No	No	Yes	No
No	No	No	No
No	No	No	Yes
No	No	No	Yes
No	No	No	Yes
No	No	No	Yes
No	No	No	Yes
No	No	No	Yes
No	No	No	Yes
No	No	Yes	No
No	No	Yes	No
No	No	Yes	No
No	No	Yes	No
No	No	Yes	No
No	No	Yes	No
No	No	Yes	Yes
No	No	Yes	Yes
No	No	Yes	Yes
No	No	Yes	Yes
No	No	Yes	Yes
No	No	Yes	Yes
No	No	No	No
No	No	No	No
No	No	No	No
No	No	No	No
No	No	Yes	No
No	No	Yes	No
No	No	Yes	No
No	No	Yes	No
No	No	Yes	No
No	Yes	Yes	Yes

No	Yes	Yes	Yes
No	Yes	Yes	Yes
No	Yes	Yes	Yes
No	Yes	Yes	Yes
No	Yes	Yes	Yes
No	Yes	Yes	Yes
Yes	No	No	No
Yes	No	No	No
Yes	No	No	No
Yes	No	No	No
Yes	No	No	No
Yes	No	No	No
Yes	No	No	No
No	No	Yes	Yes
No	No	Yes	Yes
No	No	Yes	Yes
No	No	Yes	Yes
No	No	Yes	Yes
No	No	Yes	Yes
No	No	Yes	Yes
No	No	Yes	Yes
No	No	Yes	Yes
No	No	Yes	Yes
No	No	Yes	Yes
No	No	Yes	Yes
No	No	Yes	Yes
No	No	Yes	Yes
No	No	Yes	Yes
No	No	Yes	Yes
No	No	No	Yes
No	No	No	Yes
No	No	No	Yes
No	No	No	Yes
No	No	No	Yes
No	No	No	Yes
No	No	Yes	Yes
No	No	Yes	Yes
No	No	Yes	Yes
No	No	Yes	Yes
No	No	Yes	Yes
No	No	Yes	Yes
No	No	Yes	Yes
No	No	Yes	Yes
No	No	Yes	Yes
No	No	No	Yes
No	No	No	Yes
No	No	No	Yes
No	No	No	Yes
No	No	No	Yes
No	No	No	Yes

No	No	No	No
No	No	No	No
No	No	No	No
No	No	No	No
No	No	No	No
No	No	No	No
No	No	Yes	Yes
No	No	Yes	Yes
No	No	Yes	Yes
No	No	Yes	Yes
No	No	Yes	Yes
No	No	Yes	Yes
No	No	Yes	Yes
No	Yes	No	Yes
No	Yes	No	Yes
No	Yes	No	Yes
No	Yes	No	Yes
No	Yes	No	Yes
No	No	No	Yes
No	No	No	Yes
No	No	No	Yes
No	No	No	Yes
No	No	No	Yes
No	No	No	Yes
No	No	No	Yes
No	No	No	Yes
No	No	No	Yes
No	No	Yes	No
No	No	Yes	No
No	No	Yes	No
No	No	Yes	No
No	No	Yes	No
No	No	Yes	No
No	No	Yes	No
No	No	Yes	No
No	No	Yes	No
No	No	Yes	No
No	No	Yes	No
No	No	Yes	No
No	No	Yes	No
No	No	Yes	No
No	No	No	No
No	No	No	No
No	No	No	No
No	No	No	No
No	No	Yes	Yes
No	No	Yes	Yes
No	No	Yes	Yes
No	No	Yes	Yes
No	No	No	Yes

No	No	No	Yes
No	No	No	Yes
No	No	No	Yes
No	No	No	Yes
No	No	No	Yes
No	No	No	Yes
No	No	No	Yes
No	No	No	Yes
No	No	No	Yes
No	No	No	Yes
No	No	No	Yes
No	No	No	Yes
No	No	No	Yes
No	No	No	Yes
No	No	No	Yes
No	No	No	Yes
Yes	Yes	No	No
Yes	Yes	No	No
Yes	Yes	No	No
Yes	Yes	No	No
Yes	Yes	No	No
Yes	Yes	No	No
Yes	Yes	No	No
Yes	No	Yes	Yes
Yes	No	Yes	Yes
Yes	No	Yes	Yes
Yes	No	Yes	Yes
Yes	No	Yes	Yes
Yes	No	Yes	Yes
Yes	No	Yes	Yes
Yes	No	Yes	Yes
No	No	No	Yes
No	No	No	Yes
No	No	No	Yes
No	No	No	Yes
No	No	No	Yes
No	No	No	Yes
No	No	No	Yes
No	No	No	Yes
No	No	No	Yes
No	No	No	Yes
No	No	No	Yes
No	No	No	Yes
No	No	No	Yes
No	No	No	Yes
No	No	No	Yes
No	No	No	Yes
No	No	No	Yes
No	No	Yes	No
No	No	Yes	No

No	No	Yes	No
No	No	Yes	No
No	No	Yes	No
No	No	Yes	No
No	No	Yes	No
No	No	Yes	No
No	No	Yes	No
No	No	Yes	No
No	No	Yes	No
No	No	Yes	No
No	No	Yes	No
Yes	No	Yes	No
Yes	No	Yes	No
Yes	No	Yes	No
Yes	No	Yes	No
Yes	No	Yes	No
Yes	No	Yes	No
Yes	Yes	Yes	Yes
Yes	Yes	Yes	Yes
Yes	Yes	Yes	Yes
Yes	Yes	Yes	Yes
Yes	Yes	Yes	Yes
No	Yes	No	Yes
No	Yes	No	Yes
No	Yes	No	Yes
No	Yes	No	Yes
No	Yes	No	Yes
No	Yes	No	No
No	Yes	No	No
No	Yes	No	No
No	Yes	No	No
No	Yes	No	No
No	Yes	No	No
No	No	No	Yes
No	No	No	Yes
No	No	No	Yes
No	No	No	Yes
No	No	No	Yes
No	No	No	Yes
No	No	Yes	No
No	No	Yes	No
No	No	Yes	No
No	No	Yes	No
Yes	No	No	No
Yes	No	No	No
Yes	No	No	No
Yes	No	No	No
Yes	No	No	No
Yes	No	No	No
No	No	No	No

No	No	No	No
No	No	No	No
No	No	No	No
No	No	No	No
No	No	No	No
No	No	No	No
No	No	No	No
Yes	Yes	No	Yes
Yes	Yes	No	Yes
Yes	Yes	No	Yes
Yes	Yes	No	Yes
Yes	Yes	No	Yes
Yes	Yes	No	Yes

Deworming	Use of Antimicrobials	Vaccination of goats agains	Reproductive disorders
Yes	Yes	Yes	Yes
Yes	Yes	Yes	Yes
Yes	Yes	Yes	Yes
Yes	Yes	Yes	Yes
Yes	Yes	Yes	Yes
Yes	Yes	Yes	Yes
Yes	Yes	Yes	Yes
Yes	Yes	Yes	Yes
Yes	Yes	Yes	Yes
Yes	Yes	Yes	Yes
Yes	Yes	Yes	Yes
Yes	Yes	Yes	Yes
Yes	No	Yes	Yes
Yes	No	Yes	Yes
Yes	No	Yes	Yes
Yes	No	Yes	Yes
Yes	No	Yes	Yes
Yes	Yes	Yes	Yes
Yes	Yes	Yes	Yes
Yes	Yes	Yes	Yes
Yes	Yes	Yes	Yes
Yes	Yes	Yes	Yes
Yes	Yes	Yes	Yes
Yes	Yes	Yes	Yes
Yes	Yes	Yes	Yes
Yes	Yes	Yes	Yes
Yes	Yes	Yes	Yes
Yes	Yes	Yes	Yes
Yes	Yes	Yes	Yes
Yes	Yes	Yes	Yes
Yes	Yes	Yes	Yes
No	Yes	Yes	Yes
No	Yes	Yes	Yes
No	Yes	Yes	Yes
No	Yes	Yes	Yes
Yes	Yes	Yes	Yes
Yes	Yes	Yes	Yes
Yes	Yes	Yes	Yes
Yes	Yes	Yes	Yes
Yes	Yes	Yes	Yes
Yes	Yes	Yes	Yes
Yes	Yes	Yes	Yes
Yes	Yes	Yes	Yes
Yes	Yes	Yes	Yes
No	Yes	No	Yes
No	Yes	No	Yes

No	Yes	No	Yes
No	Yes	No	Yes
No	Yes	No	Yes
No	Yes	No	Yes
No	Yes	No	Yes
No	Yes	No	Yes
No	Yes	No	Yes
No	Yes	No	Yes
No	Yes	No	Yes
No	Yes	No	Yes
No	Yes	No	Yes
No	Yes	No	Yes
No	Yes	No	Yes
No	Yes	No	Yes
No	Yes	No	Yes
No	Yes	No	Yes
No	Yes	No	Yes
No	Yes	No	Yes
No	Yes	No	Yes
Yes	No	No	Yes
Yes	No	No	Yes
Yes	No	No	Yes
Yes	No	No	Yes
Yes	No	No	Yes
Yes	No	Yes	Yes
Yes	No	Yes	Yes
Yes	No	Yes	Yes
Yes	No	Yes	Yes
Yes	No	Yes	Yes
Yes	No	Yes	Yes
Yes	No	Yes	Yes
No	Yes	No	Yes
No	Yes	No	Yes
No	Yes	No	Yes
No	Yes	No	Yes
No	Yes	No	Yes
No	Yes	No	Yes
No	Yes	No	Yes
No	Yes	No	Yes
Yes	Yes	No	Yes
Yes	Yes	No	Yes
Yes	Yes	No	Yes
Yes	Yes	No	Yes
Yes	Yes	No	Yes
Yes	Yes	No	Yes
Yes	Yes	No	Yes
Yes	Yes	No	Yes
No	No	No	Yes
No	No	No	Yes
No	No	No	Yes

Yes	No	No	Yes
Yes	No	No	Yes
Yes	No	No	Yes
Yes	No	No	Yes
Yes	No	No	Yes
Yes	No	No	Yes
Yes	No	No	Yes
Yes	No	No	Yes
No	Yes	Yes	Yes
No	Yes	Yes	Yes
No	Yes	Yes	Yes
No	Yes	Yes	Yes
No	Yes	Yes	Yes
No	No	No	No
No	No	No	No
No	No	No	No
No	No	No	No
No	No	No	No
No	Yes	Yes	Yes
No	Yes	Yes	Yes
No	Yes	Yes	Yes
No	Yes	Yes	Yes
No	Yes	Yes	Yes
No	No	Yes	Yes
No	No	Yes	Yes
No	No	Yes	Yes
No	No	Yes	Yes
No	No	Yes	Yes
No	No	Yes	Yes
Yes	No	No	Yes
Yes	No	No	Yes
Yes	No	No	Yes
Yes	No	No	Yes
Yes	No	No	Yes
No	No	Yes	Yes
No	No	Yes	Yes
No	No	Yes	Yes
No	No	Yes	Yes
No	No	Yes	Yes
No	No	Yes	Yes
No	No	Yes	No
No	No	Yes	No
No	No	Yes	No
No	No	Yes	No
No	No	Yes	No
No	No	Yes	Yes
No	No	Yes	Yes
No	No	Yes	Yes
No	No	Yes	Yes
No	No	Yes	Yes

No	No	Yes	Yes
No	No	Yes	Yes
Yes	Yes	Yes	Yes
Yes	Yes	Yes	Yes
Yes	Yes	Yes	Yes
Yes	Yes	Yes	Yes
No	No	Yes	Yes
No	No	Yes	Yes
No	No	Yes	Yes
No	No	Yes	Yes
No	No	No	No
No	No	No	No
No	No	No	No
No	No	No	No
No	Yes	Yes	Yes
No	Yes	Yes	Yes
No	Yes	Yes	Yes
No	Yes	Yes	Yes
No	Yes	Yes	Yes
No	Yes	Yes	Yes
No	Yes	Yes	Yes
No	Yes	Yes	Yes
No	Yes	Yes	Yes
Yes	No	Yes	Yes
Yes	No	Yes	Yes
Yes	No	Yes	Yes
Yes	No	Yes	Yes
Yes	No	Yes	Yes
Yes	No	Yes	Yes
Yes	Yes	No	Yes
Yes	Yes	No	Yes
Yes	Yes	No	Yes
Yes	Yes	No	Yes
Yes	Yes	No	Yes
Yes	No	No	Yes
Yes	No	No	Yes
Yes	No	No	Yes
Yes	No	No	Yes
Yes	No	No	Yes
Yes	No	No	Yes
No	No	Yes	Yes
No	No	Yes	Yes
No	No	Yes	Yes
No	No	Yes	Yes
No	No	Yes	Yes
Yes	No	Yes	Yes
Yes	No	Yes	Yes
Yes	No	Yes	Yes
Yes	No	Yes	Yes
No	No	Yes	Yes

No	No	Yes	Yes
No	No	Yes	Yes
No	No	Yes	Yes
No	No	Yes	Yes
No	No	Yes	Yes
No	No	Yes	Yes
No	No	Yes	Yes
No	No	Yes	Yes
No	No	Yes	Yes
No	No	Yes	Yes
No	No	Yes	Yes
No	No	Yes	Yes
No	No	Yes	Yes
No	No	Yes	Yes
No	No	Yes	Yes
Yes	No	No	Yes
Yes	No	No	Yes
Yes	No	No	Yes
Yes	No	No	Yes
Yes	No	No	Yes
No	No	No	Yes
No	No	No	Yes
No	No	No	Yes
No	No	No	Yes
No	No	No	Yes
No	No	No	Yes
No	No	No	Yes
No	No	No	Yes
Yes	No	No	Yes
Yes	No	No	Yes
Yes	No	No	Yes
Yes	No	No	Yes
Yes	No	No	Yes
Yes	No	No	Yes
No	No	Yes	Yes
No	No	Yes	Yes
No	No	Yes	Yes
No	No	Yes	Yes
No	No	Yes	Yes
No	No	Yes	Yes
Yes	Yes	No	Yes
Yes	Yes	No	Yes
Yes	Yes	No	Yes
Yes	Yes	No	Yes
Yes	No	No	Yes
Yes	No	No	Yes
Yes	No	No	Yes
Yes	No	No	Yes
Yes	No	No	Yes
No	Yes	Yes	Yes

No	Yes	Yes	Yes
No	Yes	Yes	Yes
No	Yes	Yes	Yes
No	Yes	Yes	Yes
No	Yes	Yes	Yes
No	Yes	Yes	Yes
Yes	No	Yes	Yes
Yes	No	Yes	Yes
Yes	No	Yes	Yes
Yes	No	Yes	Yes
Yes	No	Yes	Yes
Yes	No	Yes	Yes
Yes	No	Yes	Yes
Yes	No	No	Yes
Yes	No	No	Yes
Yes	No	No	Yes
Yes	No	No	Yes
Yes	No	No	Yes
Yes	No	No	Yes
Yes	No	No	Yes
Yes	No	No	Yes
Yes	No	No	Yes
Yes	No	No	Yes
Yes	No	No	Yes
Yes	No	No	Yes
Yes	No	No	Yes
Yes	No	No	Yes
Yes	No	No	Yes
Yes	No	No	Yes
Yes	Yes	No	Yes
Yes	Yes	No	Yes
Yes	Yes	No	Yes
Yes	Yes	No	Yes
Yes	Yes	No	Yes
Yes	Yes	No	Yes
No	No	Yes	Yes
No	No	Yes	Yes
No	No	Yes	Yes
No	No	Yes	Yes
No	No	Yes	Yes
No	No	Yes	Yes
No	No	Yes	Yes
No	No	Yes	Yes
No	No	Yes	Yes
No	No	No	Yes
No	No	No	Yes
No	No	No	Yes
No	No	No	Yes
No	No	No	Yes
No	No	No	Yes

Yes	Yes	No	Yes
Yes	Yes	No	Yes
Yes	Yes	No	Yes
Yes	Yes	No	Yes
Yes	Yes	No	Yes
Yes	Yes	No	Yes
No	No	Yes	Yes
No	No	Yes	Yes
No	No	Yes	Yes
No	No	Yes	Yes
No	No	Yes	Yes
No	No	Yes	Yes
No	No	Yes	Yes
No	No	Yes	Yes
No	No	Yes	Yes
No	No	Yes	Yes
No	No	Yes	Yes
No	No	Yes	Yes
No	No	Yes	Yes
No	No	Yes	Yes
No	No	Yes	Yes
Yes	No	No	Yes
Yes	No	No	Yes
Yes	No	No	Yes
Yes	No	No	Yes
Yes	No	No	No
Yes	No	No	No
Yes	No	Yes	Yes
Yes	No	Yes	Yes
Yes	No	Yes	Yes
Yes	No	Yes	Yes
Yes	No	Yes	Yes
Yes	No	Yes	Yes
Yes	No	Yes	Yes
Yes	No	Yes	Yes
Yes	No	Yes	Yes
Yes	No	Yes	Yes
Yes	No	Yes	Yes
Yes	No	Yes	Yes
Yes	No	Yes	Yes
Yes	No	Yes	Yes
Yes	No	Yes	Yes
Yes	No	Yes	Yes
Yes	No	Yes	Yes
Yes	No	Yes	Yes
Yes	No	No	Yes
Yes	No	No	Yes
Yes	No	No	Yes
Yes	No	No	Yes
No	No	Yes	Yes

No	No	Yes	Yes
No	No	Yes	Yes
No	No	Yes	Yes
No	No	Yes	Yes
No	No	Yes	Yes
No	No	Yes	Yes
No	No	Yes	Yes
No	No	Yes	Yes
No	No	Yes	Yes
No	No	Yes	Yes
No	No	Yes	Yes
No	No	Yes	Yes
No	No	Yes	Yes
No	No	Yes	Yes
No	No	Yes	Yes
No	No	Yes	Yes
Yes	Yes	Yes	Yes
Yes	Yes	Yes	Yes
Yes	Yes	Yes	Yes
Yes	Yes	Yes	Yes
Yes	Yes	Yes	Yes
Yes	Yes	Yes	Yes
Yes	Yes	Yes	Yes
No	No	Yes	Yes
No	No	Yes	Yes
No	No	Yes	Yes
No	No	Yes	Yes
No	No	Yes	Yes
No	No	Yes	Yes
No	No	Yes	Yes
No	No	Yes	Yes
No	No	Yes	No
No	No	Yes	No
No	No	Yes	No
No	No	Yes	No
No	No	Yes	No
No	No	Yes	No
No	No	Yes	No
No	No	Yes	No
No	No	Yes	No
No	No	Yes	No
Yes	No	No	Yes
Yes	No	No	Yes
Yes	No	No	Yes
Yes	No	No	Yes
Yes	No	No	Yes
Yes	No	No	Yes
Yes	No	No	Yes
Yes	No	Yes	Yes
Yes	No	Yes	Yes

Yes	No	Yes	Yes
Yes	No	Yes	Yes
Yes	No	Yes	Yes
Yes	No	Yes	Yes
Yes	No	Yes	Yes
Yes	No	Yes	Yes
Yes	No	Yes	Yes
Yes	No	Yes	Yes
Yes	No	Yes	Yes
Yes	No	Yes	Yes
Yes	No	Yes	Yes
Yes	Yes	No	Yes
Yes	Yes	No	Yes
Yes	Yes	No	Yes
Yes	Yes	No	Yes
Yes	Yes	No	Yes
Yes	Yes	No	Yes
Yes	No	Yes	Yes
Yes	No	Yes	Yes
Yes	No	Yes	Yes
Yes	No	Yes	Yes
Yes	No	Yes	Yes
Yes	Yes	No	Yes
Yes	Yes	No	Yes
Yes	Yes	No	Yes
Yes	Yes	No	Yes
Yes	Yes	No	Yes
Yes	Yes	Yes	Yes
Yes	Yes	Yes	Yes
Yes	Yes	Yes	Yes
Yes	Yes	Yes	Yes
Yes	Yes	Yes	Yes
Yes	Yes	Yes	Yes
Yes	Yes	No	Yes
Yes	Yes	No	Yes
Yes	Yes	No	Yes
Yes	Yes	No	Yes
Yes	Yes	No	Yes
Yes	Yes	No	Yes
Yes	No	No	Yes
Yes	No	No	Yes
Yes	No	No	Yes
Yes	No	No	Yes
Yes	No	Yes	Yes
Yes	No	Yes	Yes
Yes	No	Yes	Yes
Yes	No	Yes	Yes
Yes	No	Yes	Yes
Yes	No	Yes	Yes
Yes	Yes	No	Yes

Yes	Yes	No	Yes
Yes	Yes	No	Yes
Yes	Yes	No	Yes
Yes	Yes	No	Yes
Yes	Yes	No	Yes
Yes	Yes	No	Yes
Yes	Yes	No	Yes
Yes	Yes	Yes	Yes
Yes	Yes	Yes	Yes
Yes	Yes	Yes	Yes
Yes	Yes	Yes	Yes
Yes	Yes	Yes	Yes
Yes	Yes	Yes	Yes

History of abortion	% abortion	Proportion of abortions
0	21.73%	21-50%
A	21.73%	21-50%
0	21.73%	21-50%
NA	21.73%	21-50%
A	21.73%	21-50%
0	21.73%	21-50%
NA	21.73%	21-50%
0	21.73%	21-50%
0	21.73%	21-50%
A	21.73%	21-50%
0	21.73%	21-50%
NA	21.73%	21-50%
A	16.66%	1-20%
0	16.66%	1-20%
NA	16.66%	1-20%
A	16.66%	1-20%
NA	16.66%	1-20%
0	27.27%	21-50%
0	27.27%	21-50%
A	27.27%	21-50%
A	27.27%	21-50%
A	27.27%	21-50%
NA	27.27%	21-50%
A	27.27%	21-50%
A	27.27%	21-50%
A	16.66%	1-20%
NA	16.66%	1-20%
0	16.66%	1-20%
A	16.66%	1-20%
NA	16.66%	1-20%
A	16.66%	1-20%
NA	16.66%	1-20%
0	20%	1-20%
NA	20%	1-20%
NA	20%	1-20%
A	20%	1-20%
0	20.83%	1-20%
NA	20.83%	1-20%
A	20.83%	1-20%
A	20.83%	1-20%
0	20.83%	1-20%
NA	20.83%	1-20%
A	20.83%	1-20%
A	20.83%	1-20%
NA	20.83%	1-20%
0	31.81%	21-50%
A	31.81%	21-50%

A	31.81%	21-50%
A	31.81%	21-50%
0	31.81%	21-50%
0	31.81%	21-50%
0	31.81%	21-50%
NA	31.81%	21-50%
A	31.81%	21-50%
A	31.81%	21-50%
A	19.04%	1-20%
0	19.04%	1-20%
A	19.04%	1-20%
A	19.04%	1-20%
0	19.04%	1-20%
0	19.04%	1-20%
NA	19.04%	1-20%
0	19.04%	1-20%
NA	19.04%	1-20%
A	19.04%	1-20%
A	19.04%	1-20%
0	50%	21-50%
A	50%	21-50%
A	50%	21-50%
0	50%	21-50%
0	50%	21-50%
A	15%	1-20%
NA	15%	1-20%
0	15%	1-20%
NA	15%	1-20%
0	15%	1-20%
A	15%	1-20%
NA	15%	1-20%
A	14.28%	1-20%
NA	14.28%	1-20%
NA	14.28%	1-20%
NA	14.28%	1-20%
A	14.28%	1-20%
A	14.28%	1-20%
0	14.28%	1-20%
0	14.28%	1-20%
0	25%	21-50%
0	25%	21-50%
A	25%	21-50%
NA	25%	21-50%
0	25%	21-50%
0	25%	21-50%
NA	25%	21-50%
A	25%	21-50%
NA	0%	0%
NA	0%	0%
0	0%	0%

0	33.33%	21-50%
NA	33.33%	21-50%
A	33.33%	21-50%
A	33.33%	21-50%
0	33.33%	21-50%
0	33.33%	21-50%
A	33.33%	21-50%
NA	33.33%	21-50%
NA	12.50%	1-20%
A	12.50%	1-20%
0	12.50%	1-20%
0	12.50%	1-20%
A	12.50%	1-20%
NA	0%	0%
NA	0%	0%
0	0%	0%
0	0%	0%
NA	0%	0%
0	14.28%	1-20%
A	14.28%	1-20%
0	14.28%	1-20%
NA	14.28%	1-20%
A	14.28%	1-20%
A	25%	21-50%
NA	25%	21-50%
0	25%	21-50%
A	25%	21-50%
A	25%	21-50%
NA	25%	21-50%
A	20%	1-20%
A	20%	1-20%
0	20%	1-20%
0	20%	1-20%
0	20%	1-20%
0	12.50%	1-20%
0	12.50%	1-20%
0	12.50%	1-20%
A	12.50%	1-20%
A	12.50%	1-20%
NA	12.50%	1-20%
NA	0%	0%
NA	0%	0%
NA	0%	0%
NA	0%	0%
0	0%	0%
0	14.28%	1-20%
0	14.28%	1-20%
A	14.28%	1-20%
A	14.28%	1-20%
0	14.28%	1-20%

A	14.28%	1-20%
NA	14.28%	1-20%
NA	33.33%	21-50%
A	33.33%	21-50%
0	33.33%	21-50%
0	33.33%	21-50%
A	16.66%	1-20%
NA	16.66%	1-20%
NA	16.66%	1-20%
0	16.66%	1-20%
NA	0%	0%
NA	0%	0%
NA	0%	0%
0	0%	0%
0	15.78%	1-20%
0	15.78%	1-20%
A	15.78%	1-20%
NA	15.78%	1-20%
NA	15.78%	1-20%
0	15.78%	1-20%
0	15.78%	1-20%
NA	15.78%	1-20%
NA	15.78%	1-20%
NA	0%	0%
0	0%	0%
NA	0%	0%
NA	0%	0%
0	0%	0%
NA	0%	0%
A	16.66%	1-20%
NA	16.66%	1-20%
0	16.66%	1-20%
0	16.66%	1-20%
NA	16.66%	1-20%
NA	17.64%	1-20%
NA	17.64%	1-20%
A	17.64%	1-20%
0	17.64%	1-20%
NA	17.64%	1-20%
0	17.64%	1-20%
NA	42.85%	21-50%
A	42.85%	21-50%
A	42.85%	21-50%
A	42.85%	21-50%
0	42.85%	21-50%
0	10%	1-20%
NA	10%	1-20%
A	10%	1-20%
0	10%	1-20%
A	16.66%	1-20%

0	16.66%	1-20%
NA	16.66%	1-20%
0	16.66%	1-20%
0	16.66%	1-20%
NA	16.66%	1-20%
0	16.66%	1-20%
0	16.66%	1-20%
A	16.66%	1-20%
NA	16%	1-20%
NA	16%	1-20%
NA	16%	1-20%
0	16%	1-20%
0	16%	1-20%
A	16%	1-20%
NA	16%	1-20%
NA	20%	1-20%
NA	20%	1-20%
0	20%	1-20%
0	20%	1-20%
A	20%	1-20%
0	20%	1-20%
NA	25%	21-50%
NA	25%	21-50%
A	25%	21-50%
0	25%	21-50%
NA	25%	21-50%
0	25%	21-50%
A	25%	21-50%
NA	17.64%	1-20%
NA	17.64%	1-20%
NA	17.64%	1-20%
0	17.64%	1-20%
NA	17.64%	1-20%
A	17.64%	1-20%
A	20%	1-20%
NA	20%	1-20%
0	20%	1-20%
NA	20%	1-20%
NA	20%	1-20%
0	20%	1-20%
NA	20%	1-20%
0	20%	1-20%
0	20%	1-20%
NA	20%	1-20%
0	20%	1-20%
A	20%	1-20%
NA	20%	1-20%
A	20%	1-20%
NA	20%	1-20%
NA	13.63%	1-20%

A	13.63%	1-20%
A	13.63%	1-20%
0	13.63%	1-20%
0	13.63%	1-20%
NA	13.63%	1-20%
NA	13.63%	1-20%
NA	20%	1-20%
0	20%	1-20%
0	20%	1-20%
A	20%	1-20%
NA	20%	1-20%
0	20%	1-20%
NA	20%	1-20%
NA	22.72%	21-50%
NA	22.72%	21-50%
0	22.72%	21-50%
A	22.72%	21-50%
NA	22.72%	21-50%
NA	22.72%	21-50%
0	22.72%	21-50%
0	22.72%	21-50%
A	22.72%	21-50%
0	16.66%	1-20%
NA	16.66%	1-20%
0	16.66%	1-20%
NA	16.66%	1-20%
A	16.66%	1-20%
NA	16.66%	1-20%
0	16.66%	1-20%
0	20%	1-20%
NA	20%	1-20%
NA	20%	1-20%
0	20%	1-20%
A	20%	1-20%
NA	20%	1-20%
A	22.22%	21-50%
NA	22.22%	21-50%
0	22.22%	21-50%
0	22.22%	21-50%
NA	22.22%	21-50%
NA	22.22%	21-50%
A	22.22%	21-50%
0	22.22%	21-50%
NA	22.22%	21-50%
NA	16.66%	1-20%
0	16.66%	1-20%
NA	16.66%	1-20%
A	16.66%	1-20%
0	16.66%	1-20%
0	16.66%	1-20%

A	28.57%	21-50%
A	28.57%	21-50%
0	28.57%	21-50%
NA	28.57%	21-50%
A	28.57%	21-50%
NA	28.57%	21-50%
A	42.85%	21-50%
NA	42.85%	21-50%
0	42.85%	21-50%
A	42.85%	21-50%
NA	42.85%	21-50%
0	42.85%	21-50%
A	42.85%	21-50%
A	20%	1-20%
A	20%	1-20%
NA	20%	1-20%
NA	20%	1-20%
0	20%	1-20%
NA	12.50%	1-20%
A	12.50%	1-20%
0	12.50%	1-20%
NA	14.28%	1-20%
A	14.28%	1-20%
0	14.28%	1-20%
A	14.28%	1-20%
NA	14.28%	1-20%
NA	14.28%	1-20%
A	20%	1-20%
NA	20%	1-20%
NA	20%	1-20%
A	20%	1-20%
NA	20%	1-20%
NA	20%	1-20%
0	20%	1-20%
NA	12.50%	1-20%
NA	12.50%	1-20%
0	12.50%	1-20%
A	12.50%	1-20%
NA	12.50%	1-20%
0	12.50%	1-20%
0	12.50%	1-20%
A	37.50%	21-50%
A	37.50%	21-50%
NA	37.50%	21-50%
A	37.50%	21-50%
NA	14.28%	1-20%
NA	14.28%	1-20%
A	14.28%	1-20%
0	14.28%	1-20%
NA	33.33%	21-50%

NA	33.33%	21-50%
A	33.33%	21-50%
0	33.33%	21-50%
0	33.33%	21-50%
A	9.09%	1-20%
NA	9.09%	1-20%
NA	9.09%	1-20%
A	9.09%	1-20%
NA	9.09%	1-20%
0	9.09%	1-20%
NA	0%	0%
NA	0%	0%
NA	0%	0%
0	0%	0%
0	0%	0%
NA	0%	0%
A	22.22%	21-50%
NA	22.22%	21-50%
0	22.22%	21-50%
0	22.22%	21-50%
A	22.22%	21-50%
0	22.22%	21-50%
NA	22.22%	21-50%
NA	23.80%	1-20%
0	23.80%	1-20%
NA	23.80%	1-20%
0	23.80%	1-20%
A	23.80%	1-20%
0	23.80%	1-20%
NA	23.80%	1-20%
A	23.80%	1-20%
0		
0		
0		
0		
0		
0		
0		
0		
0		
0		
A	11.76%	1-20%
NA	11.76%	1-20%
NA	11.76%	1-20%
0	11.76%	1-20%
NA	11.76%	1-20%
NA	11.76%	1-20%
0	11.76%	1-20%
NA	18.75%	1-20%
0	18.75%	1-20%

NA	18.75%	1-20%
NA	18.75%	1-20%
NA	18.75%	1-20%
NA	18.75%	1-20%
0	18.75%	1-20%
0	18.75%	1-20%
A	18.75%	1-20%
A	18.75%	1-20%
0	18.75%	1-20%
A	18.75%	1-20%
NA	18.75%	1-20%
NA	14.28%	1-20%
A	14.28%	1-20%
NA	14.28%	1-20%
0	14.28%	1-20%
0	14.28%	1-20%
0	14.28%	1-20%
NA	0%	0%
NA	0%	0%
0	0%	0%
0	0%	0%
0	0%	0%
NA	42.85%	21-50%
A	42.85%	21-50%
NA	42.85%	21-50%
A	42.85%	21-50%
NA	42.85%	21-50%
NA	0%	0%
0	0%	0%
NA	0%	0%
NA	0%	0%
NA	0%	0%
NA	0%	0%
NA	15%	1-20%
0	15%	1-20%
A	15%	1-20%
NA	15%	1-20%
A	15%	1-20%
0	15%	1-20%
0	0%	0%
NA	0%	0%
NA	0%	0%
0	0%	0%
0	16.66%	1-20%
NA	16.66%	1-20%
0	16.66%	1-20%
A	16.66%	1-20%
0	16.66%	1-20%
NA	16.66%	1-20%
NA	29%	21-50%

NA	29%	21-50%
0	29%	21-50%
NA	29%	21-50%
NA	29%	21-50%
A	29%	21-50%
A	29%	21-50%
NA	29%	21-50%
A	8.69%	1-20%
0	8.69%	1-20%
0	8.69%	1-20%
NA	8.69%	1-20%
NA	8.69%	1-20%
NA	8.69%	1-20%

Number of abortions in the last year	Elimination of aborted products	Stillbirth
(1-5)	Yes	Yes
(1-5)	Yes	Yes
(1-5)	Yes	Yes
(1-5)	Yes	Yes
(1-5)	Yes	Yes
(1-5)	Yes	Yes
(1-5)	Yes	Yes
(1-5)	Yes	Yes
(1-5)	Yes	Yes
(1-5)	Yes	Yes
(1-5)	Yes	Yes
(1-5)	Yes	Yes
(1-5)	No	No
(1-5)	No	No
(1-5)	No	No
(1-5)	No	No
(1-5)	No	No
(1-5)	Yes	No
(1-5)	Yes	No
(1-5)	Yes	No
(1-5)	Yes	No
(1-5)	Yes	No
(1-5)	Yes	No
(1-5)	Yes	No
(1-5)	Yes	No
(1-5)	No	Yes
(1-5)	No	Yes
(1-5)	No	Yes
(1-5)	No	Yes
(1-5)	No	Yes
(1-5)	No	Yes
(1-5)	No	Yes
(1-5)	No	No
(1-5)	No	No
(1-5)	No	No
(1-5)	No	No
(6-10)	Yes	Yes
(6-10)	Yes	Yes
(6-10)	Yes	Yes
(6-10)	Yes	Yes
(6-10)	Yes	Yes
(6-10)	Yes	Yes
(6-10)	Yes	Yes
(6-10)	Yes	Yes
(6-10)	Yes	Yes
(6-10)	No	Yes
(6-10)	No	Yes

(6-10)	No	Yes
(6-10)	No	Yes
(6-10)	No	Yes
(6-10)	No	Yes
(6-10)	No	Yes
(6-10)	No	Yes
(6-10)	No	Yes
(6-10)	No	Yes
(1-5)	No	Yes
(1-5)	No	Yes
(1-5)	No	Yes
(1-5)	No	Yes
(1-5)	No	Yes
(1-5)	No	Yes
(1-5)	No	Yes
(1-5)	No	Yes
(1-5)	No	Yes
(1-5)	No	Yes
(1-5)	No	Yes
(1-5)	Yes	Yes
(1-5)	Yes	Yes
(1-5)	Yes	Yes
(1-5)	Yes	Yes
(1-5)	Yes	Yes
(1-5)	No	No
(1-5)	No	No
(1-5)	No	No
(1-5)	No	No
(1-5)	No	No
(1-5)	No	No
(1-5)	No	No
(1-5)	Yes	Yes
(1-5)	Yes	Yes
(1-5)	Yes	Yes
(1-5)	Yes	Yes
(1-5)	Yes	Yes
(1-5)	Yes	Yes
(1-5)	Yes	Yes
(1-5)	Yes	Yes
(1-5)	No	Yes
(1-5)	No	Yes
(1-5)	No	Yes
(1-5)	No	Yes
(1-5)	No	Yes
(1-5)	No	Yes
(1-5)	No	Yes
(1-5)	No	Yes
0	Yes	Yes
0	Yes	Yes
0	Yes	Yes

(6-10)	No	No
(6-10)	No	No
(6-10)	No	No
(6-10)	No	No
(6-10)	No	No
(6-10)	No	No
(6-10)	No	No
(6-10)	No	No
(1-5)	No	Yes
(1-5)	No	Yes
(1-5)	No	Yes
(1-5)	No	Yes
(1-5)	No	Yes
0	No	No
0	No	No
0	No	No
0	No	No
0	No	No
(1-5)	No	Yes
(1-5)	No	Yes
(1-5)	No	Yes
(1-5)	No	Yes
(1-5)	No	Yes
(1-5)	Yes	Yes
(1-5)	Yes	Yes
(1-5)	Yes	Yes
(1-5)	Yes	Yes
(1-5)	Yes	Yes
(1-5)	Yes	Yes
(1-5)	No	No
(1-5)	No	No
(1-5)	No	No
(1-5)	No	No
(1-5)	No	No
(1-5)	No	No
(1-5)	No	No
(1-5)	No	No
(1-5)	No	No
(1-5)	No	No
(1-5)	No	No
0	No	No
0	No	No
0	No	No
0	No	No
0	No	No
(1-5)	No	Yes
(1-5)	No	Yes
(1-5)	No	Yes
(1-5)	No	Yes
(1-5)	No	Yes

(1-5)	No	Yes
(1-5)	No	Yes
(1-5)	No	No
(1-5)	No	No
(1-5)	No	No
(1-5)	No	No
(1-5)	No	Yes
(1-5)	No	Yes
(1-5)	No	Yes
(1-5)	No	Yes
0		
0		
0		
0		
(1-5)	No	Yes
(1-5)	No	Yes
(1-5)	No	Yes
(1-5)	No	Yes
(1-5)	No	Yes
(1-5)	No	Yes
(1-5)	No	Yes
(1-5)	No	Yes
(1-5)	No	Yes
0		Yes
0		Yes
0		Yes
0		Yes
0		Yes
0		Yes
(1-5)	No	Yes
(1-5)	No	Yes
(1-5)	No	Yes
(1-5)	No	Yes
(1-5)	No	Yes
(1-5)	No	Yes
(1-5)	No	Yes
(1-5)	No	Yes
(1-5)	No	Yes
(1-5)	No	Yes
(1-5)	No	Yes
(1-5)	Yes	Yes
(1-5)	Yes	Yes
(1-5)	Yes	Yes
(1-5)	Yes	Yes
(1-5)	Yes	Yes
(1-5)	No	Yes
(1-5)	No	Yes
(1-5)	No	Yes
(1-5)	No	Yes
(1-5)	No	No

(1-5)	No	No
(1-5)	No	No
(1-5)	No	No
(1-5)	No	No
(1-5)	No	No
(1-5)	No	No
(1-5)	No	No
(1-5)	No	No
(1-5)	No	Yes
(1-5)	No	Yes
(1-5)	No	Yes
(1-5)	No	Yes
(1-5)	No	Yes
(1-5)	No	Yes
(1-5)	No	Yes
(1-5)	No	Yes
(1-5)	No	Yes
(1-5)	No	Yes
(1-5)	No	Yes
(1-5)	No	Yes
(1-5)	No	No
(1-5)	No	Yes
(1-5)	No	Yes
(1-5)	No	Yes
(1-5)	No	Yes
(1-5)	No	Yes
(1-5)	No	Yes
(1-5)	No	Yes
(1-5)	No	No
(1-5)	No	No
(1-5)	No	No
(1-5)	No	No
(1-5)	No	No
(1-5)	No	No
(1-5)	No	Yes
(1-5)	No	Yes
(1-5)	No	Yes
(1-5)	No	Yes
(1-5)	No	Yes
(1-5)	No	Yes
(1-5)	No	Yes
(1-5)	No	Yes
(1-5)	No	Yes
(1-5)	No	Yes
(1-5)	No	No
(1-5)	No	No
(1-5)	No	No
(1-5)	No	No
(1-5)	No	No
(1-5)	No	Yes

(1-5)	No	Yes
(1-5)	No	Yes
(1-5)	No	Yes
(1-5)	No	Yes
(1-5)	No	Yes
(1-5)	No	Yes
(1-5)	Yes	Yes
(1-5)	Yes	Yes
(1-5)	Yes	Yes
(1-5)	Yes	Yes
(1-5)	Yes	Yes
(1-5)	Yes	Yes
(1-5)	Yes	Yes
(1-5)	No	Yes
(1-5)	No	Yes
(1-5)	No	Yes
(1-5)	No	Yes
(1-5)	No	Yes
(1-5)	No	Yes
(1-5)	No	Yes
(1-5)	No	Yes
(1-5)	No	Yes
(1-5)	Yes	Yes
(1-5)	Yes	Yes
(1-5)	Yes	Yes
(1-5)	Yes	Yes
(1-5)	Yes	Yes
(1-5)	Yes	Yes
(1-5)	Yes	Yes
(1-5)	No	Yes
(1-5)	No	Yes
(1-5)	No	Yes
(1-5)	No	Yes
(1-5)	No	Yes
(1-5)	No	Yes
(1-5)	No	Yes
(1-5)	No	Yes
(1-5)	No	Yes
(1-5)	No	Yes
(1-5)	No	Yes
(1-5)	No	Yes
(1-5)	No	Yes
(1-5)	No	Yes
(1-5)	No	Yes
(1-5)	No	Yes
(1-5)	No	Yes
(1-5)	No	Yes
(1-5)	No	Yes
(1-5)	No	Yes
(1-5)	No	Yes

(1-5)	No	Yes
(1-5)	No	Yes
(1-5)	No	Yes
(1-5)	No	Yes
(1-5)	No	Yes
(1-5)	No	Yes
(1-5)	No	Yes
(1-5)	No	Yes
(1-5)	No	Yes
(1-5)	No	Yes
(1-5)	No	Yes
(1-5)	No	Yes
(1-5)	No	Yes
(1-5)	No	No
(1-5)	No	No
(1-5)	No	No
(1-5)	No	No
(1-5)	No	No
(1-5)	No	Yes
(1-5)	No	Yes
(1-5)	No	Yes
(1-5)	No	No
(1-5)	No	No
(1-5)	No	No
(1-5)	No	No
(1-5)	No	No
(1-5)	No	No
(1-5)	Yes	No
(1-5)	Yes	No
(1-5)	Yes	No
(1-5)	Yes	No
(1-5)	Yes	No
(1-5)	Yes	No
(1-5)	Yes	No
(1-5)	No	Yes
(1-5)	No	Yes
(1-5)	No	Yes
(1-5)	No	Yes
(1-5)	No	Yes
(1-5)	No	Yes
(1-5)	No	Yes
(6-10)	No	No
(6-10)	No	No
(6-10)	No	No
(6-10)	No	No
(1-5)	No	Yes
(1-5)	No	Yes
(1-5)	No	Yes
(1-5)	No	Yes
(1-5)	No	Yes

(1-5)	No	Yes
(1-5)	No	Yes
(1-5)	No	Yes
(1-5)	No	Yes
(1-5)	No	No
(1-5)	No	No
(1-5)	No	No
(1-5)	No	No
(1-5)	No	No
(1-5)	No	No
0		Yes
0		Yes
0		Yes
0		Yes
0		Yes
0		Yes
(1-5)	No	Yes
(1-5)	No	Yes
(1-5)	No	Yes
(1-5)	No	Yes
(1-5)	No	Yes
(1-5)	No	Yes
(1-5)	No	Yes
(1-5)	No	Yes
(1-5)	No	Yes
(1-5)	No	Yes
(1-5)	No	Yes
(1-5)	No	Yes
(1-5)	No	Yes
(1-5)	No	Yes
(1-5)	No	Yes
(1-5)	No	Yes
(1-5)	No	Yes
(1-5)	No	Yes
(1-5)	No	Yes
(1-5)	No	Yes
(1-5)	No	Yes
(1-5)	No	Yes
(1-5)	No	Yes
(1-5)	No	Yes

(1-5)	No	Yes
(1-5)	No	Yes
(1-5)	No	Yes
(1-5)	No	Yes
(1-5)	No	Yes
(1-5)	No	Yes
(1-5)	No	Yes
(1-5)	No	Yes
(1-5)	No	Yes
(1-5)	No	Yes
(1-5)	No	Yes
(1-5)	Yes	No
(1-5)	Yes	No
(1-5)	Yes	No
(1-5)	Yes	No
(1-5)	Yes	No
(1-5)	Yes	No
0		Yes
0		Yes
0		Yes
0		Yes
0		Yes
(1-5)	Yes	No
(1-5)	Yes	No
(1-5)	Yes	No
(1-5)	Yes	No
(1-5)	Yes	No
0		Yes
0		Yes
0		Yes
0		Yes
0		Yes
0		Yes
(1-5)	Yes	Yes
(1-5)	Yes	Yes
(1-5)	Yes	Yes
(1-5)	Yes	Yes
(1-5)	Yes	Yes
(1-5)	Yes	Yes
0		No
0		No
0		No
0		No
(1-5)	Yes	Yes
(1-5)	Yes	Yes
(1-5)	Yes	Yes
(1-5)	Yes	Yes
(1-5)	Yes	Yes
(1-5)	Yes	Yes
(1-5)	No	Yes

(1-5)	No	Yes
(1-5)	No	Yes
(1-5)	No	Yes
(1-5)	No	Yes
(1-5)	No	Yes
(1-5)	No	Yes
(1-5)	No	Yes
(1-5)	Yes	Yes
(1-5)	Yes	Yes
(1-5)	Yes	Yes
(1-5)	Yes	Yes
(1-5)	Yes	Yes
(1-5)	Yes	Yes

New born death	Province	Region	Environment	Season	Climate
Yes	Mila	Plateau	Peri-urban	Spring	Sub-humid
Yes	Mila	Plateau	Peri-urban	Spring	Sub-humid
Yes	Mila	Plateau	Peri-urban	Spring	Sub-humid
Yes	Mila	Plateau	Peri-urban	Spring	Sub-humid
Yes	Mila	Plateau	Peri-urban	Spring	Sub-humid
Yes	Mila	Plateau	Peri-urban	Spring	Sub-humid
Yes	Mila	Plateau	Peri-urban	Spring	Sub-humid
Yes	Mila	Plateau	Peri-urban	Spring	Sub-humid
Yes	Mila	Plateau	Peri-urban	Spring	Sub-humid
Yes	Mila	Plateau	Peri-urban	Spring	Sub-humid
Yes	Mila	Plateau	Peri-urban	Spring	Sub-humid
Yes	Mila	Plateau	Peri-urban	Spring	Sub-humid
No	Mila	Mountainous	Rural	Winter	Humid
No	Mila	Mountainous	Rural	Winter	Humid
No	Mila	Mountainous	Rural	Winter	Humid
No	Mila	Mountainous	Rural	Winter	Humid
No	Mila	Mountainous	Rural	Winter	Humid
Yes	Mila	Mountainous	Rural	Summer	Humid
Yes	Mila	Mountainous	Rural	Summer	Humid
Yes	Mila	Mountainous	Rural	Summer	Humid
Yes	Mila	Mountainous	Rural	Summer	Humid
Yes	Mila	Mountainous	Rural	Summer	Humid
Yes	Mila	Mountainous	Rural	Summer	Humid
Yes	Mila	Mountainous	Rural	Summer	Humid
Yes	Mila	Mountainous	Rural	Summer	Humid
Yes	Mila	Mountainous	Rural	Summer	Humid
Yes	Mila	Mountainous	Rural	Summer	Humid
Yes	Mila	Mountainous	Rural	Summer	Humid
Yes	Mila	Mountainous	Rural	Summer	Humid
Yes	Mila	Mountainous	Rural	Summer	Humid
Yes	Mila	Mountainous	Rural	Summer	Humid
Yes	Mila	Mountainous	Rural	Summer	Humid
No	Mila	Plateau	Rural	Summer	Humid
No	Mila	Plateau	Rural	Summer	Humid
No	Mila	Plateau	Rural	Summer	Humid
No	Mila	Plateau	Rural	Summer	Humid
No	Mila	Plateau	Rural	Winter	Humid
No	Mila	Plateau	Rural	Winter	Humid
No	Mila	Plateau	Rural	Winter	Humid
No	Mila	Plateau	Rural	Winter	Humid
No	Mila	Plateau	Rural	Winter	Humid
No	Mila	Plateau	Rural	Winter	Humid
No	Mila	Plateau	Rural	Winter	Humid
No	Mila	Plateau	Rural	Winter	Humid
No	Mila	Plateau	Rural	Winter	Humid
Yes	Mila	Plateau	Rural	Spring	Humid
Yes	Mila	Plateau	Rural	Spring	Humid

Yes	Mila	Plateau	Rural	Spring	Humid
Yes	Mila	Plateau	Rural	Spring	Humid
Yes	Mila	Plateau	Rural	Spring	Humid
Yes	Mila	Plateau	Rural	Spring	Humid
Yes	Mila	Plateau	Rural	Spring	Humid
Yes	Mila	Plateau	Rural	Spring	Humid
Yes	Mila	Plateau	Rural	Spring	Humid
Yes	Mila	Plateau	Rural	Spring	Humid
No	Mila	Mountainous	Rural	Spring	Sub-humid
No	Mila	Mountainous	Rural	Spring	Sub-humid
No	Mila	Mountainous	Rural	Spring	Sub-humid
No	Mila	Mountainous	Rural	Spring	Sub-humid
No	Mila	Mountainous	Rural	Spring	Sub-humid
No	Mila	Mountainous	Rural	Spring	Sub-humid
No	Mila	Mountainous	Rural	Spring	Sub-humid
No	Mila	Mountainous	Rural	Spring	Sub-humid
No	Mila	Mountainous	Rural	Spring	Sub-humid
No	Mila	Mountainous	Rural	Spring	Sub-humid
No	Mila	Mountainous	Rural	Spring	Sub-humid
No	Mila	Plateau	Peri-urban	Summer	Sub-humid
No	Mila	Plateau	Peri-urban	Summer	Sub-humid
No	Mila	Plateau	Peri-urban	Summer	Sub-humid
No	Mila	Plateau	Peri-urban	Summer	Sub-humid
No	Mila	Plateau	Peri-urban	Summer	Sub-humid
No	Mila	Plateau	Rural	Winter	Humid
No	Mila	Plateau	Rural	Winter	Humid
No	Mila	Plateau	Rural	Winter	Humid
No	Mila	Plateau	Rural	Winter	Humid
No	Mila	Plateau	Rural	Winter	Humid
No	Mila	Plateau	Rural	Winter	Humid
No	Mila	Plateau	Rural	Winter	Humid
Yes	Mila	Plateau	Rural	Spring	Sub-humid
Yes	Mila	Plateau	Rural	Spring	Sub-humid
Yes	Mila	Plateau	Rural	Spring	Sub-humid
Yes	Mila	Plateau	Rural	Spring	Sub-humid
Yes	Mila	Plateau	Rural	Spring	Sub-humid
Yes	Mila	Plateau	Rural	Spring	Sub-humid
Yes	Mila	Plateau	Rural	Spring	Sub-humid
Yes	Mila	Plateau	Rural	Spring	Sub-humid
No	Mila	Mountainous	Rural	Winter	Humid
No	Mila	Mountainous	Rural	Winter	Humid
No	Mila	Mountainous	Rural	Winter	Humid
No	Mila	Mountainous	Rural	Winter	Humid
No	Mila	Mountainous	Rural	Winter	Humid
No	Mila	Mountainous	Rural	Winter	Humid
No	Mila	Mountainous	Rural	Winter	Humid
No	Mila	Mountainous	Rural	Winter	Humid
No	Mila	Plateau	Peri-urban	Spring	Sub-humid
No	Mila	Plateau	Peri-urban	Spring	Sub-humid
No	Mila	Plateau	Peri-urban	Spring	Sub-humid

Yes	Mila	Plateau	Rural	Spring	Sub-humid
Yes	Mila	Plateau	Rural	Spring	Sub-humid
Yes	Mila	Plateau	Rural	Spring	Sub-humid
Yes	Mila	Plateau	Rural	Spring	Sub-humid
Yes	Mila	Plateau	Rural	Spring	Sub-humid
Yes	Mila	Plateau	Rural	Spring	Sub-humid
Yes	Mila	Plateau	Rural	Spring	Sub-humid
Yes	Mila	Plateau	Rural	Spring	Sub-humid
No	Mila	Plateau	Peri-urban	Winter	Sub-humid
No	Mila	Plateau	Peri-urban	Winter	Sub-humid
No	Mila	Plateau	Peri-urban	Winter	Sub-humid
No	Mila	Plateau	Peri-urban	Winter	Sub-humid
No	Mila	Plateau	Peri-urban	Winter	Sub-humid
No	Mila	Plateau	Rural	Winter	Humid
No	Mila	Plateau	Rural	Winter	Humid
No	Mila	Plateau	Rural	Winter	Humid
No	Mila	Plateau	Rural	Winter	Humid
No	Mila	Plateau	Rural	Winter	Humid
No	Mila	Plateau	Peri-urban	Spring	Humid
No	Mila	Plateau	Peri-urban	Spring	Humid
No	Mila	Plateau	Peri-urban	Spring	Humid
No	Mila	Plateau	Peri-urban	Spring	Humid
No	Mila	Plateau	Peri-urban	Spring	Humid
No	Mila	Plateau	Rural	Spring	Sub-humid
No	Mila	Plateau	Rural	Spring	Sub-humid
No	Mila	Plateau	Rural	Spring	Sub-humid
No	Mila	Plateau	Rural	Spring	Sub-humid
No	Mila	Plateau	Rural	Spring	Sub-humid
No	Mila	Plateau	Rural	Spring	Sub-humid
No	Mila	Mountainous	Rural	Winter	Humid
No	Mila	Mountainous	Rural	Winter	Humid
No	Mila	Mountainous	Rural	Winter	Humid
No	Mila	Mountainous	Rural	Winter	Humid
No	Mila	Mountainous	Rural	Winter	Humid
No	Mila	Plateau	Rural	Spring	Sub-humid
No	Mila	Plateau	Rural	Spring	Sub-humid
No	Mila	Plateau	Rural	Spring	Sub-humid
No	Mila	Plateau	Rural	Spring	Sub-humid
No	Mila	Plateau	Rural	Spring	Sub-humid
No	Mila	Plateau	Rural	Spring	Sub-humid
No	Constantin	Mountainous	Rural	Summer	Sub-humid
No	Constantin	Mountainous	Rural	Summer	Sub-humid
No	Constantin	Mountainous	Rural	Summer	Sub-humid
No	Constantin	Mountainous	Rural	Summer	Sub-humid
No	Constantin	Mountainous	Rural	Summer	Sub-humid
No	Constantin	Plateau	Rural	Spring	Humid
No	Constantin	Plateau	Rural	Spring	Humid
No	Constantin	Plateau	Rural	Spring	Humid
No	Constantin	Plateau	Rural	Spring	Humid
No	Constantin	Plateau	Rural	Spring	Humid

No	Constantin	Plateau	Rural	Spring	Humid
No	Constantin	Plateau	Rural	Spring	Humid
Yes	Constantin	Mountainous	Rural	Winter	Sub-humid
Yes	Constantin	Mountainous	Rural	Winter	Sub-humid
Yes	Constantin	Mountainous	Rural	Winter	Sub-humid
Yes	Constantin	Mountainous	Rural	Winter	Sub-humid
No	Mila	Plateau	Rural	Spring	Humid
No	Mila	Plateau	Rural	Spring	Humid
No	Mila	Plateau	Rural	Spring	Humid
No	Mila	Plateau	Rural	Spring	Humid
	Constantin	Mountainous	Peri-urban	Summer	Sub-humid
	Constantin	Mountainous	Peri-urban	Summer	Sub-humid
	Constantin	Mountainous	Peri-urban	Summer	Sub-humid
	Constantin	Mountainous	Peri-urban	Summer	Sub-humid
No	Constantin	Plateau	Rural	Spring	Sub-humid
No	Constantin	Plateau	Rural	Spring	Sub-humid
No	Constantin	Plateau	Rural	Spring	Sub-humid
No	Constantin	Plateau	Rural	Spring	Sub-humid
No	Constantin	Plateau	Rural	Spring	Sub-humid
No	Constantin	Plateau	Rural	Spring	Sub-humid
No	Constantin	Plateau	Rural	Spring	Sub-humid
No	Constantin	Plateau	Rural	Spring	Sub-humid
No	Constantin	Plateau	Rural	Spring	Sub-humid
No	Constantin	Plateau	Rural	Spring	Humid
No	Constantin	Plateau	Rural	Spring	Humid
No	Constantin	Plateau	Rural	Spring	Humid
No	Constantin	Plateau	Rural	Spring	Humid
No	Constantin	Plateau	Rural	Spring	Humid
No	Constantin	Plateau	Rural	Spring	Humid
No	Constantin	Plateau	Rural	Winter	Humid
No	Constantin	Plateau	Rural	Winter	Humid
No	Constantin	Plateau	Rural	Winter	Humid
No	Constantin	Plateau	Rural	Winter	Humid
No	Constantin	Plateau	Rural	Winter	Humid
No	Constantin	Mountainous	Rural	Winter	Sub-humid
No	Constantin	Mountainous	Rural	Winter	Sub-humid
No	Constantin	Mountainous	Rural	Winter	Sub-humid
No	Constantin	Mountainous	Rural	Winter	Sub-humid
Yes	Constantin	Mountainous	Rural	Winter	Sub-humid
Yes	Constantin	Mountainous	Rural	Winter	Sub-humid
No	Mila	Plateau	Peri-urban	Spring	Humid
No	Mila	Plateau	Peri-urban	Spring	Humid
No	Mila	Plateau	Peri-urban	Spring	Humid
No	Mila	Plateau	Peri-urban	Spring	Humid
No	Mila	Plateau	Peri-urban	Spring	Humid
No	Constantin	Plateau	Rural	Winter	Sub-humid
No	Constantin	Plateau	Rural	Winter	Sub-humid
No	Constantin	Plateau	Rural	Winter	Sub-humid
No	Constantin	Plateau	Rural	Winter	Sub-humid
Yes	Constantin	Plateau	Peri-urban	Autumn	Humid

Yes	Constantin	Plateau	Peri-urban	Autumn	Humid
Yes	Constantin	Plateau	Peri-urban	Autumn	Humid
Yes	Constantin	Plateau	Peri-urban	Autumn	Humid
Yes	Constantin	Plateau	Peri-urban	Autumn	Humid
Yes	Constantin	Plateau	Peri-urban	Autumn	Humid
Yes	Constantin	Plateau	Peri-urban	Autumn	Humid
Yes	Constantin	Plateau	Peri-urban	Autumn	Humid
Yes	Constantin	Plateau	Peri-urban	Autumn	Humid
Yes	Constantin	Plateau	Rural	Summer	Humid
Yes	Constantin	Plateau	Rural	Summer	Humid
Yes	Constantin	Plateau	Rural	Summer	Humid
Yes	Constantin	Plateau	Rural	Summer	Humid
Yes	Constantin	Plateau	Rural	Summer	Humid
Yes	Constantin	Plateau	Rural	Summer	Humid
Yes	Constantin	Plateau	Rural	Summer	Humid
No	Constantin	Plateau	Rural	Spring	Sub-humid
No	Constantin	Plateau	Rural	Spring	Sub-humid
No	Constantin	Plateau	Rural	Spring	Sub-humid
No	Constantin	Plateau	Rural	Spring	Sub-humid
No	Constantin	Plateau	Rural	Spring	Sub-humid
No	Constantin	Plateau	Rural	Spring	Sub-humid
No	Mila	Plateau	Rural	Summer	Humid
No	Mila	Plateau	Rural	Summer	Humid
No	Mila	Plateau	Rural	Summer	Humid
No	Mila	Plateau	Rural	Summer	Humid
No	Mila	Plateau	Rural	Summer	Humid
No	Mila	Plateau	Rural	Summer	Humid
No	Mila	Plateau	Rural	Summer	Humid
Yes	Mila	Mountainous	Rural	Winter	Semi-arid
Yes	Mila	Mountainous	Rural	Winter	Semi-arid
Yes	Mila	Mountainous	Rural	Winter	Semi-arid
Yes	Mila	Mountainous	Rural	Winter	Semi-arid
Yes	Mila	Mountainous	Rural	Winter	Semi-arid
Yes	Mila	Mountainous	Rural	Winter	Semi-arid
Yes	Constantin	Plateau	Peri-urban	Spring	Sub-humid
Yes	Constantin	Plateau	Peri-urban	Spring	Sub-humid
Yes	Constantin	Plateau	Peri-urban	Spring	Sub-humid
Yes	Constantin	Plateau	Peri-urban	Spring	Sub-humid
Yes	Constantin	Plateau	Peri-urban	Spring	Sub-humid
Yes	Constantin	Plateau	Peri-urban	Spring	Sub-humid
No	Constantin	Plateau	Rural	Autumn	Humid
No	Constantin	Plateau	Rural	Autumn	Humid
No	Constantin	Plateau	Rural	Autumn	Humid
No	Constantin	Plateau	Rural	Autumn	Humid
No	Constantin	Mountainous	Rural	Spring	Sub-humid
No	Constantin	Mountainous	Rural	Spring	Sub-humid
No	Constantin	Mountainous	Rural	Spring	Sub-humid
No	Constantin	Mountainous	Rural	Spring	Sub-humid
No	Constantin	Mountainous	Rural	Spring	Sub-humid
No	Mila	Mountainous	Rural	Summer	Humid

No	Mila	Mountainous	Rural	Summer	Humid
No	Mila	Mountainous	Rural	Summer	Humid
No	Mila	Mountainous	Rural	Summer	Humid
No	Mila	Mountainous	Rural	Summer	Humid
No	Mila	Mountainous	Rural	Summer	Humid
No	Mila	Mountainous	Rural	Summer	Humid
No	Mila	Plateau	Peri-urban	Summer	Sub-humid
No	Mila	Plateau	Peri-urban	Summer	Sub-humid
No	Mila	Plateau	Peri-urban	Summer	Sub-humid
No	Mila	Plateau	Peri-urban	Summer	Sub-humid
No	Mila	Plateau	Peri-urban	Summer	Sub-humid
No	Mila	Plateau	Peri-urban	Summer	Sub-humid
No	Mila	Plateau	Peri-urban	Summer	Sub-humid
No	Mila	Plateau	Rural	Autumn	Sub-humid
No	Mila	Plateau	Rural	Autumn	Sub-humid
No	Mila	Plateau	Rural	Autumn	Sub-humid
No	Mila	Plateau	Rural	Autumn	Sub-humid
No	Mila	Plateau	Rural	Autumn	Sub-humid
No	Mila	Plateau	Rural	Autumn	Sub-humid
No	Mila	Plateau	Rural	Autumn	Sub-humid
No	Mila	Plateau	Rural	Autumn	Sub-humid
No	Mila	Plateau	Rural	Autumn	Sub-humid
Yes	Mila	Plateau	Rural	Autumn	Sub-humid
Yes	Mila	Plateau	Rural	Autumn	Sub-humid
No	Mila	Plateau	Rural	Autumn	Sub-humid
No	Mila	Plateau	Rural	Autumn	Sub-humid
No	Mila	Plateau	Rural	Autumn	Sub-humid
No	Mila	Plateau	Rural	Autumn	Sub-humid
No	Mila	Plateau	Rural	Autumn	Sub-humid
Yes	Constantin	Mountainous	Rural	Winter	Sub-humid
Yes	Constantin	Mountainous	Rural	Winter	Sub-humid
Yes	Constantin	Mountainous	Rural	Winter	Sub-humid
Yes	Constantin	Mountainous	Rural	Winter	Sub-humid
Yes	Constantin	Mountainous	Rural	Winter	Sub-humid
Yes	Constantin	Mountainous	Rural	Winter	Sub-humid
No	Constantin	Plateau	Rural	Spring	Sub-humid
No	Constantin	Plateau	Rural	Spring	Sub-humid
No	Constantin	Plateau	Rural	Spring	Sub-humid
No	Constantin	Plateau	Rural	Spring	Sub-humid
No	Constantin	Plateau	Rural	Spring	Sub-humid
No	Constantin	Plateau	Rural	Spring	Sub-humid
No	Constantin	Plateau	Rural	Spring	Sub-humid
No	Constantin	Plateau	Rural	Spring	Sub-humid
No	Constantin	Plateau	Rural	Spring	Sub-humid
No	Mila	Mountainous	Rural	Autumn	Humid
No	Mila	Mountainous	Rural	Autumn	Humid
No	Mila	Mountainous	Rural	Autumn	Humid
No	Mila	Mountainous	Rural	Autumn	Humid
No	Mila	Mountainous	Rural	Autumn	Humid
No	Mila	Mountainous	Rural	Autumn	Humid

No	Mila	Mountainous	Rural	Spring	Humid
No	Mila	Mountainous	Rural	Spring	Humid
No	Mila	Mountainous	Rural	Spring	Humid
No	Mila	Mountainous	Rural	Spring	Humid
No	Mila	Mountainous	Rural	Spring	Humid
No	Mila	Mountainous	Rural	Spring	Humid
Yes	Constantin	Plateau	Rural	Autumn	Sub-humid
Yes	Constantin	Plateau	Rural	Autumn	Sub-humid
Yes	Constantin	Plateau	Rural	Autumn	Sub-humid
Yes	Constantin	Plateau	Rural	Autumn	Sub-humid
Yes	Constantin	Plateau	Rural	Autumn	Sub-humid
Yes	Constantin	Plateau	Rural	Autumn	Sub-humid
Yes	Constantin	Plateau	Rural	Autumn	Sub-humid
Yes	Constantin	Plateau	Rural	Winter	Humid
Yes	Constantin	Plateau	Rural	Winter	Humid
Yes	Constantin	Plateau	Rural	Winter	Humid
Yes	Constantin	Plateau	Rural	Winter	Humid
Yes	Constantin	Plateau	Rural	Winter	Humid
No	Mila	Mountainous	Rural	Spring	Humid
No	Mila	Mountainous	Rural	Spring	Humid
No	Mila	Mountainous	Rural	Spring	Humid
Yes	Constantin	Mountainous	Rural	Winter	Sub-humid
Yes	Constantin	Mountainous	Rural	Winter	Sub-humid
Yes	Constantin	Mountainous	Rural	Winter	Sub-humid
Yes	Constantin	Mountainous	Rural	Winter	Sub-humid
Yes	Constantin	Mountainous	Rural	Winter	Sub-humid
Yes	Constantin	Mountainous	Rural	Winter	Sub-humid
Yes	Constantin	Plateau	Rural	Spring	Humid
Yes	Constantin	Plateau	Rural	Spring	Humid
Yes	Constantin	Plateau	Rural	Spring	Humid
Yes	Constantin	Plateau	Rural	Spring	Humid
Yes	Constantin	Plateau	Rural	Spring	Humid
Yes	Constantin	Plateau	Rural	Spring	Humid
Yes	Constantin	Plateau	Rural	Spring	Humid
Yes	Constantin	Plateau	Peri-urban	Spring	Sub-humid
Yes	Constantin	Plateau	Peri-urban	Spring	Sub-humid
Yes	Constantin	Plateau	Peri-urban	Spring	Sub-humid
Yes	Constantin	Plateau	Peri-urban	Spring	Sub-humid
Yes	Constantin	Plateau	Peri-urban	Spring	Sub-humid
Yes	Constantin	Plateau	Peri-urban	Spring	Sub-humid
Yes	Constantin	Plateau	Peri-urban	Spring	Sub-humid
No	Mila	Plateau	Rural	Winter	Humid
No	Mila	Plateau	Rural	Winter	Humid
No	Mila	Plateau	Rural	Winter	Humid
No	Mila	Plateau	Rural	Winter	Humid
Yes	Mila	Mountainous	Rural	Spring	Sub-humid
Yes	Mila	Mountainous	Rural	Spring	Sub-humid
Yes	Mila	Mountainous	Rural	Spring	Sub-humid
Yes	Mila	Mountainous	Rural	Spring	Sub-humid
No	Constantin	Mountainous	Rural	Summer	Sub-humid

No	Constantin	Mountainous	Rural	Summer	Sub-humid
No	Constantin	Mountainous	Rural	Summer	Sub-humid
No	Constantin	Mountainous	Rural	Summer	Sub-humid
No	Constantin	Mountainous	Rural	Summer	Sub-humid
Yes	Mila	Mountainous	Rural	Spring	Humid
Yes	Mila	Mountainous	Rural	Spring	Humid
Yes	Mila	Mountainous	Rural	Spring	Humid
Yes	Mila	Mountainous	Rural	Spring	Humid
Yes	Mila	Mountainous	Rural	Spring	Humid
Yes	Mila	Mountainous	Rural	Spring	Humid
Yes	Guelma	Mountainous	Peri-urban	Autumn	Sub-humid
Yes	Guelma	Mountainous	Peri-urban	Autumn	Sub-humid
Yes	Guelma	Mountainous	Peri-urban	Autumn	Sub-humid
Yes	Guelma	Mountainous	Peri-urban	Autumn	Sub-humid
Yes	Guelma	Mountainous	Peri-urban	Autumn	Sub-humid
Yes	Guelma	Mountainous	Peri-urban	Autumn	Sub-humid
No	Guelma	Mountainous	Peri-urban	Spring	Humid
No	Guelma	Mountainous	Peri-urban	Spring	Humid
No	Guelma	Mountainous	Peri-urban	Spring	Humid
No	Guelma	Mountainous	Peri-urban	Spring	Humid
No	Guelma	Mountainous	Peri-urban	Spring	Humid
No	Guelma	Mountainous	Peri-urban	Spring	Humid
No	Guelma	Mountainous	Peri-urban	Spring	Humid
No	Guelma	Plateau	Rural	Winter	Sub-humid
No	Guelma	Plateau	Rural	Winter	Sub-humid
No	Guelma	Plateau	Rural	Winter	Sub-humid
No	Guelma	Plateau	Rural	Winter	Sub-humid
No	Guelma	Plateau	Rural	Winter	Sub-humid
No	Guelma	Plateau	Rural	Winter	Sub-humid
No	Guelma	Plateau	Rural	Winter	Sub-humid
No	Guelma	Plateau	Rural	Winter	Sub-humid
	El-Taref	Coastal	Rural	Summer	Humid
	El-Taref	Coastal	Rural	Summer	Humid
	El-Taref	Coastal	Rural	Summer	Humid
	El-Taref	Coastal	Rural	Summer	Humid
	El-Taref	Coastal	Rural	Summer	Humid
	El-Taref	Coastal	Rural	Summer	Humid
	El-Taref	Coastal	Rural	Summer	Humid
	El-Taref	Coastal	Rural	Summer	Humid
	El-Taref	Coastal	Rural	Summer	Humid
	El-Taref	Coastal	Rural	Summer	Humid
Yes	Guelma	Plateau	Rural	Autumn	Semi-arid
Yes	Guelma	Plateau	Rural	Autumn	Semi-arid
Yes	Guelma	Plateau	Rural	Autumn	Semi-arid
Yes	Guelma	Plateau	Rural	Autumn	Semi-arid
Yes	Guelma	Plateau	Rural	Autumn	Semi-arid
Yes	Guelma	Plateau	Rural	Autumn	Semi-arid
Yes	Guelma	Plateau	Rural	Autumn	Semi-arid
No	Guelma	Mountainous	Rural	Spring	Sub-humid
No	Guelma	Mountainous	Rural	Spring	Sub-humid

No	Guelma	Mountainous	Rural	Spring	Sub-humid
No	Guelma	Mountainous	Rural	Spring	Sub-humid
No	Guelma	Mountainous	Rural	Spring	Sub-humid
No	Guelma	Mountainous	Rural	Spring	Sub-humid
No	Guelma	Mountainous	Rural	Spring	Sub-humid
No	Guelma	Mountainous	Rural	Spring	Sub-humid
No	Guelma	Mountainous	Rural	Spring	Sub-humid
No	Guelma	Mountainous	Rural	Spring	Sub-humid
No	Guelma	Mountainous	Rural	Spring	Sub-humid
No	Guelma	Mountainous	Rural	Spring	Sub-humid
No	Guelma	Mountainous	Rural	Spring	Sub-humid
Yes	Mila	Mountainous	Rural	Summer	Humid
Yes	Mila	Mountainous	Rural	Summer	Humid
Yes	Mila	Mountainous	Rural	Summer	Humid
Yes	Mila	Mountainous	Rural	Summer	Humid
Yes	Mila	Mountainous	Rural	Summer	Humid
Yes	Mila	Mountainous	Rural	Summer	Humid
No	Mila	Mountainous	Rural	Summer	Humid
No	Mila	Mountainous	Rural	Summer	Humid
No	Mila	Mountainous	Rural	Summer	Humid
No	Mila	Mountainous	Rural	Summer	Humid
No	Mila	Mountainous	Rural	Summer	Humid
Yes	Mila	Plateau	Rural	Spring	Humid
Yes	Mila	Plateau	Rural	Spring	Humid
Yes	Mila	Plateau	Rural	Spring	Humid
Yes	Mila	Plateau	Rural	Spring	Humid
Yes	Mila	Plateau	Rural	Spring	Humid
No	Mila	Mountainous	Rural	Spring	Humid
No	Mila	Mountainous	Rural	Spring	Humid
No	Mila	Mountainous	Rural	Spring	Humid
No	Mila	Mountainous	Rural	Spring	Humid
No	Mila	Mountainous	Rural	Spring	Humid
No	Mila	Mountainous	Rural	Spring	Humid
No	Mila	Mountainous	Rural	Summer	Humid
No	Mila	Mountainous	Rural	Summer	Humid
No	Mila	Mountainous	Rural	Summer	Humid
No	Mila	Mountainous	Rural	Summer	Humid
No	Mila	Mountainous	Rural	Summer	Humid
No	Mila	Mountainous	Rural	Summer	Humid
No	Mila	Plateau	Rural	Summer	Humid
No	Mila	Plateau	Rural	Summer	Humid
No	Mila	Plateau	Rural	Summer	Humid
No	Mila	Plateau	Rural	Summer	Humid
No	Mila	Mountainous	Rural	Spring	Humid
No	Mila	Mountainous	Rural	Spring	Humid
No	Mila	Mountainous	Rural	Spring	Humid
No	Mila	Mountainous	Rural	Spring	Humid
No	Mila	Mountainous	Rural	Spring	Humid
No	Mila	Mountainous	Rural	Spring	Humid
No	Mila	Mountainous	Rural	Winter	Humid

No	Mila	Mountainous	Rural	Winter	Humid
No	Mila	Mountainous	Rural	Winter	Humid
No	Mila	Mountainous	Rural	Winter	Humid
No	Mila	Mountainous	Rural	Winter	Humid
No	Mila	Mountainous	Rural	Winter	Humid
No	Mila	Mountainous	Rural	Winter	Humid
No	Mila	Mountainous	Rural	Winter	Humid
Yes	Mila	Mountainous	Rural	Spring	Humid
Yes	Mila	Mountainous	Rural	Spring	Humid
Yes	Mila	Mountainous	Rural	Spring	Humid
Yes	Mila	Mountainous	Rural	Spring	Humid
Yes	Mila	Mountainous	Rural	Spring	Humid
Yes	Mila	Mountainous	Rural	Spring	Humid

Month of samp	Year of sam	ELISA Resu	N° sample
March	2020	0	1
March	2020	0	2
March	2020	0	3
March	2020	0	4
March	2020	0	5
March	2020	1	6
March	2020	1	7
March	2020	1	8
March	2020	1	9
March	2020	1	10
March	2020	1	11
March	2020	1	12
January	2020	1	13
January	2020	1	14
January	2020	1	15
January	2020	1	16
January	2020	1	17
June	2020	1	18
June	2020	1	19
June	2020	1	20
June	2020	1	21
June	2020	1	22
June	2020	1	23
June	2020	1	24
June	2020	1	25
june	2020	1	26
June	2020	1	27
June	2020	1	28
June	2020	1	29
June	2020	1	30
June	2020	0	31
June	2020	1	32
June	2020	1	33
June	2020	1	34
June	2020	1	35
June	2020	1	36
January	2020	1	37
January	2020	1	38
January	2020	1	39
January	2020	1	40
January	2020	0	41
January	2020	0	42
January	2020	0	43
January	2020	1	44
January	2020	1	45
March	2020	1	46
March	2020	1	47

March	2020	1	48
March	2020	0	49
March	2020	0	50
March	2020	0	51
March	2020	0	52
March	2020	0	53
March	2020	1	54
March	2020	1	55
April	2020	0	56
April	2020	0	57
April	2020	0	58
April	2020	0	59
April	2020	0	60
April	2020	1	61
April	2020	1	62
April	2020	0	63
April	2020	0	64
April	2020	1	65
April	2020	0	66
Juley	2020	0	67
Juley	2020	0	68
Juley	2020	0	69
Juley	2020	0	70
Juley	2020	1	71
January	2020	1	72
January	2020	1	73
January	2020	1	74
January	2020	1	75
January	2020	0	76
January	2020	1	77
January	2020	0	78
April	2020	0	79
April	2020	1	80
April	2020	1	81
April	2020	1	82
April	2020	1	83
April	2020	1	84
April	2020	1	85
April	2020	1	86
January	2021	1	87
January	2021	1	88
January	2021	1	89
January	2021	1	90
January	2021	0	91
January	2021	0	92
January	2021	0	93
January	2021	0	94
March	2021	0	95
March	2021	1	96
March	2021	1	97

March	2021	0	98
March	2021	1	99
March	2021	1	100
March	2021	0	101
March	2021	1	102
March	2021	0	103
March	2021	0	104
March	2021	0	105
January	2021	0	106
January	2021	1	107
January	2021	1	108
January	2021	0	109
January	2021	0	110
February	2021	1	111
February	2021	0	112
February	2021	0	113
February	2021	0	114
February	2021	0	115
March	2021	0	116
March	2021	0	117
March	2021	0	118
March	2021	0	119
March	2021	0	120
April	2021	1	121
April	2021	0	122
April	2021	0	123
April	2021	1	124
April	2021	1	125
April	2021	1	126
February	2021	1	127
February	2021	0	128
February	2021	1	129
February	2021	1	130
February	2021	0	131
April	2021	0	132
April	2021	0	133
April	2021	0	134
April	2021	1	135
April	2021	1	136
April	2021	0	137
June	2021	1	140
June	2021	0	141
June	2021	0	142
June	2021	1	143
June	2021	0	144
May	2021	0	145
May	2021	1	146
May	2021	1	147
May	2021	0	148
May	2021	0	149

May	2021	0	150
May	2021	1	151
January	2021	0	153
January	2021	0	154
January	2021	1	155
January	2021	0	156
April	2021	0	157
April	2021	1	158
April	2021	0	159
April	2021	1	160
June	2021	0	161
June	2021	0	162
June	2021	0	163
June	2021	1	164
May	2021	0	165
May	2021	0	166
May	2021	1	167
May	2021	0	168
May	2021	0	169
May	2021	1	170
May	2021	0	171
May	2021	0	172
May	2021	1	173
May	2021	1	174
May	2021	1	175
May	2021	1	176
May	2021	1	177
May	2021	1	178
May	2021	1	179
January	2021	0	180
January	2021	1	181
January	2021	0	182
January	2021	1	183
January	2021	0	184
February	2021	0	185
February	2021	0	186
February	2021	0	187
February	2021	1	188
February	2021	0	189
February	2021	0	190
March	2021	1	191
March	2021	0	192
March	2021	1	193
March	2021	1	194
March	2021	1	195
January	2022	1	196
January	2022	1	197
January	2022	1	198
January	2022	1	199
September	2022	1	200

September	2022	1	201
September	2022	1	202
September	2022	1	203
September	2022	1	204
September	2022	1	205
September	2022	1	206
September	2022	1	207
September	2022	1	208
June	2022	0	209
June	2022	1	210
June	2022	1	211
June	2022	1	212
June	2022	1	213
June	2022	1	214
June	2022	0	215
April	2022	0	216
April	2022	0	217
April	2022	0	218
April	2022	0	219
April	2022	1	220
April	2022	1	221
June	2022	1	222
June	2022	1	223
June	2022	0	224
June	2022	1	225
June	2022	1	226
June	2022	1	227
June	2022	1	228
February	2022	0	229
February	2022	1	230
February	2022	1	231
February	2022	1	232
February	2022	1	233
February	2022	0	234
March	2022	1	235
March	2022	0	236
March	2022	0	237
March	2022	0	238
March	2022	0	239
March	2022	1	240
September	2022	1	241
September	2022	1	242
September	2022	1	243
September	2022	1	244
March	2022	1	245
March	2022	0	246
March	2022	1	247
March	2022	0	248
March	2022	1	249
June	2022	0	250

June	2022	1	251
June	2022	0	252
June	2022	1	253
June	2022	1	254
June	2022	1	255
June	2022	1	256
June	2022	1	257
June	2022	1	258
June	2022	0	259
June	2022	0	260
June	2022	1	261
June	2022	1	262
June	2022	1	263
September	2022	1	264
September	2022	1	265
September	2022	0	266
September	2022	1	267
September	2022	0	268
September	2022	1	269
September	2022	1	270
September	2022	1	271
September	2022	1	272
October	2022	1	273
October	2022	0	274
October	2022	1	275
October	2022	1	276
October	2022	1	277
October	2022	0	278
October	2022	0	279
February	2022	1	280
February	2022	0	281
February	2022	1	282
February	2022	0	283
February	2022	1	284
February	2022	0	285
March	2022	1	286
March	2022	0	287
March	2022	1	288
March	2022	0	289
March	2022	1	290
March	2022	1	291
March	2022	1	292
March	2022	1	293
March	2022	1	294
September	2022	0	295
September	2022	1	296
September	2022	0	297
September	2022	0	298
September	2022	1	299
September	2022	0	300

April	2022	1	301
April	2022	1	302
April	2022	0	303
April	2022	1	304
April	2022	1	305
April	2022	1	306
October	2022	1	307
October	2022	1	308
October	2022	1	309
October	2022	1	310
October	2022	1	311
October	2022	1	312
October	2022	1	313
January	2022	1	314
January	2022	1	315
January	2022	1	316
January	2022	1	317
January	2022	0	318
March	2022	0	319
March	2022	1	320
March	2022	0	321
February	2022	0	322
February	2022	1	323
February	2022	1	324
February	2022	0	325
February	2022	0	326
February	2022	0	327
April	2022	0	328
April	2022	0	329
April	2022	0	330
April	2022	1	331
April	2022	0	332
April	2022	0	333
April	2022	0	334
April	2022	0	335
April	2022	0	336
April	2022	0	337
April	2022	1	338
April	2022	1	339
April	2022	0	340
April	2022	0	341
February	2022	0	342
February	2022	0	343
February	2022	0	344
February	2022	0	345
March	2022	0	346
March	2022	1	347
March	2022	0	348
March	2022	1	349
Juley	2022	0	350

Juley	2022	1	351
Juley	2022	0	352
Juley	2022	0	353
Juley	2022	0	354
May	2022	1	355
May	2022	1	356
May	2022	0	357
May	2022	0	358
May	2022	0	359
May	2022	1	360
October	2021	1	361
October	2021	0	362
October	2021	1	363
October	2021	1	364
October	2021	1	365
October	2021	0	366
March	2021	1	367
March	2021	0	368
March	2021	0	369
March	2021	1	370
March	2021	1	371
March	2021	1	372
March	2021	0	373
January	2021	1	374
January	2021	0	375
January	2021	0	376
January	2021	1	377
January	2021	1	378
January	2021	0	379
January	2021	0	380
January	2021	1	381
Juley	2021	1	382
Juley	2021	1	383
Juley	2021	1	384
Juley	2021	1	385
Juley	2021	1	386
Juley	2021	0	387
Juley	2021	0	388
Juley	2021	1	389
Juley	2021	0	390
Juley	2021	1	391
October	2021	0	392
October	2021	0	393
October	2021	1	394
October	2021	1	395
October	2021	0	396
October	2021	1	397
October	2021	1	398
April	2021	0	399
April	2021	1	400

April	2021	0	401
April	2021	1	402
April	2021	1	403
April	2021	1	404
April	2021	0	405
April	2021	1	406
April	2021	1	407
April	2021	0	408
April	2021	1	409
April	2021	0	410
April	2021	0	411
Juley	2022	1	570
Juley	2022	0	571
Juley	2022	0	572
Juley	2022	0	573
Juley	2022	0	574
Juley	2022	0	575
Juley	2022	0	576
Juley	2022	0	577
Juley	2022	0	578
Juley	2022	0	579
Juley	2022	0	580
March	2022	0	581
March	2022	0	582
March	2022	1	583
March	2022	1	584
March	2022	1	585
April	2022	1	586
April	2022	0	587
April	2022	0	588
April	2022	1	589
April	2022	0	590
April	2022	0	591
Juley	2022	0	592
Juley	2022	1	593
Juley	2022	1	594
Juley	2022	0	595
Juley	2022	0	596
Juley	2022	0	597
Juley	2022	0	598
Juley	2022	0	599
Juley	2022	0	600
Juley	2022	0	601
April	2022	0	602
April	2022	0	603
April	2022	0	604
April	2022	1	605
April	2022	0	606
April	2022	0	607
February	2023	0	608

February	2023	0	609
February	2023	0	610
February	2023	0	611
February	2023	0	612
February	2023	0	613
February	2023	1	614
February	2023	0	615
March	2023	0	616
March	2023	0	617
March	2023	0	618
March	2023	1	619
March	2023	0	620
March	2023	1	621
